# The SuperCam Instrument Suite on the NASA Mars 2020 Rover: Body Unit and Combined System Tests

**DOI:** 10.1007/s11214-020-00777-5

**Published:** 2020-12-21

**Authors:** Roger C. Wiens, Sylvestre Maurice, Scott H. Robinson, Anthony E. Nelson, Philippe Cais, Pernelle Bernardi, Raymond T. Newell, Sam Clegg, Shiv K. Sharma, Steven Storms, Jonathan Deming, Darrel Beckman, Ann M. Ollila, Olivier Gasnault, Ryan B. Anderson, Yves André, S. Michael Angel, Gorka Arana, Elizabeth Auden, Pierre Beck, Joseph Becker, Karim Benzerara, Sylvain Bernard, Olivier Beyssac, Louis Borges, Bruno Bousquet, Kerry Boyd, Michael Caffrey, Jeffrey Carlson, Kepa Castro, Jorden Celis, Baptiste Chide, Kevin Clark, Edward Cloutis, Elizabeth C. Cordoba, Agnes Cousin, Magdalena Dale, Lauren Deflores, Dorothea Delapp, Muriel Deleuze, Matthew Dirmyer, Christophe Donny, Gilles Dromart, M. George Duran, Miles Egan, Joan Ervin, Cecile Fabre, Amaury Fau, Woodward Fischer, Olivier Forni, Thierry Fouchet, Reuben Fresquez, Jens Frydenvang, Denine Gasway, Ivair Gontijo, John Grotzinger, Xavier Jacob, Sophie Jacquinod, Jeffrey R. Johnson, Roberta A. Klisiewicz, James Lake, Nina Lanza, Javier Laserna, Jeremie Lasue, Stéphane Le Mouélic, Carey Legett, Richard Leveille, Eric Lewin, Guillermo Lopez-Reyes, Ralph Lorenz, Eric Lorigny, Steven P. Love, Briana Lucero, Juan Manuel Madariaga, Morten Madsen, Soren Madsen, Nicolas Mangold, Jose Antonio Manrique, J. P. Martinez, Jesus Martinez-Frias, Kevin P. McCabe, Timothy H. McConnochie, Justin M. McGlown, Scott M. McLennan, Noureddine Melikechi, Pierre-Yves Meslin, John M. Michel, David Mimoun, Anupam Misra, Gilles Montagnac, Franck Montmessin, Valerie Mousset, Naomi Murdoch, Horton Newsom, Logan A. Ott, Zachary R. Ousnamer, Laurent Pares, Yann Parot, Rafal Pawluczyk, C. Glen Peterson, Paolo Pilleri, Patrick Pinet, Gabriel Pont, Francois Poulet, Cheryl Provost, Benjamin Quertier, Heather Quinn, William Rapin, Jean-Michel Reess, Amy H. Regan, Adriana L. Reyes-Newell, Philip J. Romano, Clement Royer, Fernando Rull, Benigno Sandoval, Joseph H. Sarrao, Violaine Sautter, Marcel J. Schoppers, Susanne Schröder, Daniel Seitz, Terra Shepherd, Pablo Sobron, Bruno Dubois, Vishnu Sridhar, Michael J. Toplis, Imanol Torre-Fdez, Ian A. Trettel, Mark Underwood, Andres Valdez, Jacob Valdez, Dawn Venhaus, Peter Willis

**Affiliations:** 1 Los Alamos National Laboratory, Los Alamos, NM USA; 2 Institut de Recherche en Astrophysique et Planetologie (IRAP), Université de Toulouse, UPS, CNRS, Toulouse, France; 3 Laboratoire d’astrophysique de Bordeaux, Univ. Bordeaux, CNRS, Bordeaux, France; 4 Laboratoire d’Etudes Spatiales et d’Instrumentation en Astrophysique, Observatoire de Paris, Meudon, France; 5 University of Hawaii, Manoa, HI USA; 6 U.S. Geological Survey Astrogeology Science Center, Flagstaff, AZ USA; 7 Centre National d’Etudes Spatiales, Toulouse, France; 8 University of South Carolina, Columbia, SC USA; 9 University of Basque Country, UPV/EHU, Bilbao, Spain; 10 Institut de Planétologie et d’Astrophysique de Grenoble, Université Grenoble Alpes, Grenoble, France; 11 Institut de Minéralogie, Physique des Matériaux et Cosmochimie, CNRS, Museum National d’Histoire Naturelle, Sorbonne Université, Paris, France; 12 Centre Lasers Intenses et Applications, University of Bordeaux, Bordeaux, France; 13 Jet Propulsion Laboratory/Caltech, Pasadena, CA USA; 14 Institut Supérieur de l’Aéronautique et de l’Espace (ISAE), Toulouse, France; 15 University of Winnipeg, Winnipeg, Canada; 16 Univ Lyon, ENSL, Univ Lyon 1, CNRS, LGL-TPE, 69364 Lyon, France; 17 GeoRessources, Université de Lorraine, Nancy, France; 18 California Institute of Technology, Pasadena, CA USA; 19 University of Copenhagen, Copenhagen, Denmark; 20 Institut de mécanique des fluides de Toulouse (CNRS, INP, Univ. Toulouse), Toulouse, France; 21 Johns Hopkins University Applied Physics Laboratory, Laurel, MD USA; 22 Universidad de Malaga, Malaga, Spain; 23 Laboratoire de Planétologie et Géodynamique, Université de Nantes, Université d’Angers, CNRS UMR 6112, Nantes, France; 24 McGill University, Montreal, Canada; 25 University of Valladolid, UVA, Valladolid, Spain; 26 Agencia Estatal Consejo Superior de Investigaciones Cientificas, Madrid, Spain; 27 University of Maryland, College Park, MD USA; 28 State University of New York, Stony Brook, NY USA; 29 University of Massachusetts, Lowell, MA USA; 30 Laboratoire Atmosphères, Milieux, Observations Spatiales, Paris, France; 31 University of New Mexico, Albuquerque, NM USA; 32 FiberTech Optica, Kitchener, ON Canada; 33 Institut d’Astrophysique Spatiale (IAS), Orsay, France; 34 Deutsches Zentrum für Luft- und Raumfahrt (DLR), Institute of Optical Sensor Systems, Berlin, Germany; 35 SETI Institute, Mountain View, CA USA; 36 Université de Toulouse; UPS-OMP, Toulouse, France

**Keywords:** Perseverance rover, LIBS, Raman spectroscopy, Infrared spectroscopy, Microphone on Mars, SuperCam, Jezero crater, Mars

## Abstract

The SuperCam instrument suite provides the Mars 2020 rover, Perseverance, with a number of versatile remote-sensing techniques that can be used at long distance as well as within the robotic-arm workspace. These include laser-induced breakdown spectroscopy (LIBS), remote time-resolved Raman and luminescence spectroscopies, and visible and infrared (VISIR; separately referred to as VIS and IR) reflectance spectroscopy. A remote micro-imager (RMI) provides high-resolution color context imaging, and a microphone can be used as a stand-alone tool for environmental studies or to determine physical properties of rocks and soils from shock waves of laser-produced plasmas. SuperCam is built in three parts: The mast unit (MU), consisting of the laser, telescope, RMI, IR spectrometer, and associated electronics, is described in a companion paper. The on-board calibration targets are described in another companion paper. Here we describe SuperCam’s body unit (BU) and testing of the integrated instrument.

The BU, mounted inside the rover body, receives light from the MU via a 5.8 m optical fiber. The light is split into three wavelength bands by a demultiplexer, and is routed via fiber bundles to three optical spectrometers, two of which (UV and violet; 245–340 and 385–465 nm) are crossed Czerny-Turner reflection spectrometers, nearly identical to their counterparts on ChemCam. The third is a high-efficiency transmission spectrometer containing an optical intensifier capable of gating exposures to 100 ns or longer, with variable delay times relative to the laser pulse. This spectrometer covers 535–853 nm ($105\text{--}7070~\text{cm}^{-1}$ Raman shift relative to the 532 nm green laser beam) with $12~\text{cm}^{-1}$ full-width at half-maximum peak resolution in the Raman fingerprint region. The BU electronics boards interface with the rover and control the instrument, returning data to the rover. Thermal systems maintain a warm temperature during cruise to Mars to avoid contamination on the optics, and cool the detectors during operations on Mars.

Results obtained with the integrated instrument demonstrate its capabilities for LIBS, for which a library of 332 standards was developed. Examples of Raman and VISIR spectroscopy are shown, demonstrating clear mineral identification with both techniques. Luminescence spectra demonstrate the utility of having both spectral and temporal dimensions. Finally, RMI and microphone tests on the rover demonstrate the capabilities of these subsystems as well.

## Introduction

NASA’s Mars rovers have used various remote-sensing instruments over the last two and a half decades. The Sojourner rover was outfitted for remote sensing with only imagers (Golombek et al. 1999). The Mars Exploration Rovers (MER) were equipped with Miniature Thermal Emission Spectrometers (Mini-TES; Christensen et al. 2003) in addition to stereo multispectral imagers (Pancam; Bell et al. 2003). Mini-TES provided the first compositional remote sensing from a Mars rover beyond imaging filter wheels. However, the surface of Mars is covered by dust, which limits the ability of passive remote-sensing devices to make observations of the mineralogy or chemistry of the underlying rocks. The Chemistry and Camera (ChemCam) instrument on the Curiosity rover overcomes this challenge by using a laser to ablate the dust and additionally enabling remote depth profiles to several hundred μm to understand the surface conditions of the rocks (Maurice et al. 2012; Wiens et al. 2012). ChemCam uses laser-induced breakdown spectroscopy (LIBS) to obtain semi-quantitative elemental abundances from rasters of small observation points 350–550 μm in diameter (Maurice et al. 2012). While ChemCam is limited mostly to chemical compositions rather than mineralogy, its ability to detect and quantify hydrogen is important for understanding the hydration state of the soils and for identifying some hydrated minerals (Schröder et al. 2015; Rapin et al. 2016, 2018, 2019; Thomas et al. 2020). ChemCam’s chemistry is complemented by visible-range (“VIS”) reflectance spectroscopy to $\sim850~\text{nm}$ that allowed Johnson et al. (2015, 2017) to constrain the mineralogy of iron-bearing materials (e.g., hematite, olivine, and ferric sulfates). However, this passive spectral range is not diagnostic for phyllosilicates and carbonates, which are important for understanding the history of Mars’ hydration, climate, and habitability. Because of Curiosity’s relative lack of remote mineral-identification capabilities, the Mars 2020 Science Definition Team mandated that the next NASA rover should possess the ability to observe mineral compositions by remote sensing (Mustard et al. 2013).

The SuperCam instrument is a response to this requirement for remote mineralogy while preserving the ability to remove dust prior to making observations of nearby targets, and providing the same or better chemistry and high-resolution imaging as ChemCam. This new instrument resulted from a happy collision of ideas from previous mission proposals. It was recognized years ago that laser-induced breakdown spectroscopy (LIBS) and remote Raman spectroscopy both required a laser, a telescope, and an optical spectrometer (e.g., Wiens et al. 2005), and members of the SuperCam team sought to make that a reality over the years. The first attempt was for the ExoMars rover (Courreges-Lacoste et al. 2007), but the LIBS was descoped early in the ExoMars development (Rull et al. 2017). A remote Raman-LIBS combination much closer in design to SuperCam was developed during the Venus Surface and Atmosphere Geochemical Explorer (SAGE) mission which only proceeded through Phase-A development (Clegg et al. 2014).

It was well understood that the Martian surface would benefit very strongly from a combination of remote Raman and visible-to-infrared (VISIR) reflectance spectroscopy. These two mineralogy techniques are highly complementary, as Raman signals occur as a result of a change in polarizability of a molecule with atomic vibrations, whereas infrared spectroscopy is sensitive to a change in the dipole moments. Raman spectroscopy is sensitive to a symmetric stretch but infrared spectroscopy is not. On the other hand, Raman can be insensitive to asymmetric stretches to which infrared spectroscopy is sensitive. The power of Raman spectroscopy originates from the fact that the activity of Raman modes depends both on the form of a vibrational harmonic and the stereochemistry of the molecule in question. Given the unexpectedly high abundances of feldspars (not generally recognized by near infrared spectroscopy) in igneous float rocks, conglomerates, and sedimentary outcrops in Aeolis Palus at Gale crater (e.g., Sautter et al. 2015, 2016), it is important to identify these minerals, a capability provided by Raman spectroscopy. Short-wave infrared spectroscopy is highly selective and diagnostic for phyllosilicates and other (polar/nonpolar) minerals, so SuperCam includes both spectroscopic techniques.

In addition, two more techniques were added. The hardware used for pulsed-laser Raman spectroscopy also enables time-resolved luminescence (TRL) spectroscopy. Luminescence is a general term that encompasses the fast, spin-allowed transitions referred to as fluorescence, and the slower, spin-forbidden transitions called phosphorescence. SuperCam can detect, distinguish, and characterize both fluorescence and phosphorescence using TRL. Finally, acoustic spectral sensing was added to remotely determine the physical properties of rocks and to assess atmospheric properties (e.g., Murdoch et al. 2019; Chide et al. 2019).

**Fig. 1:**
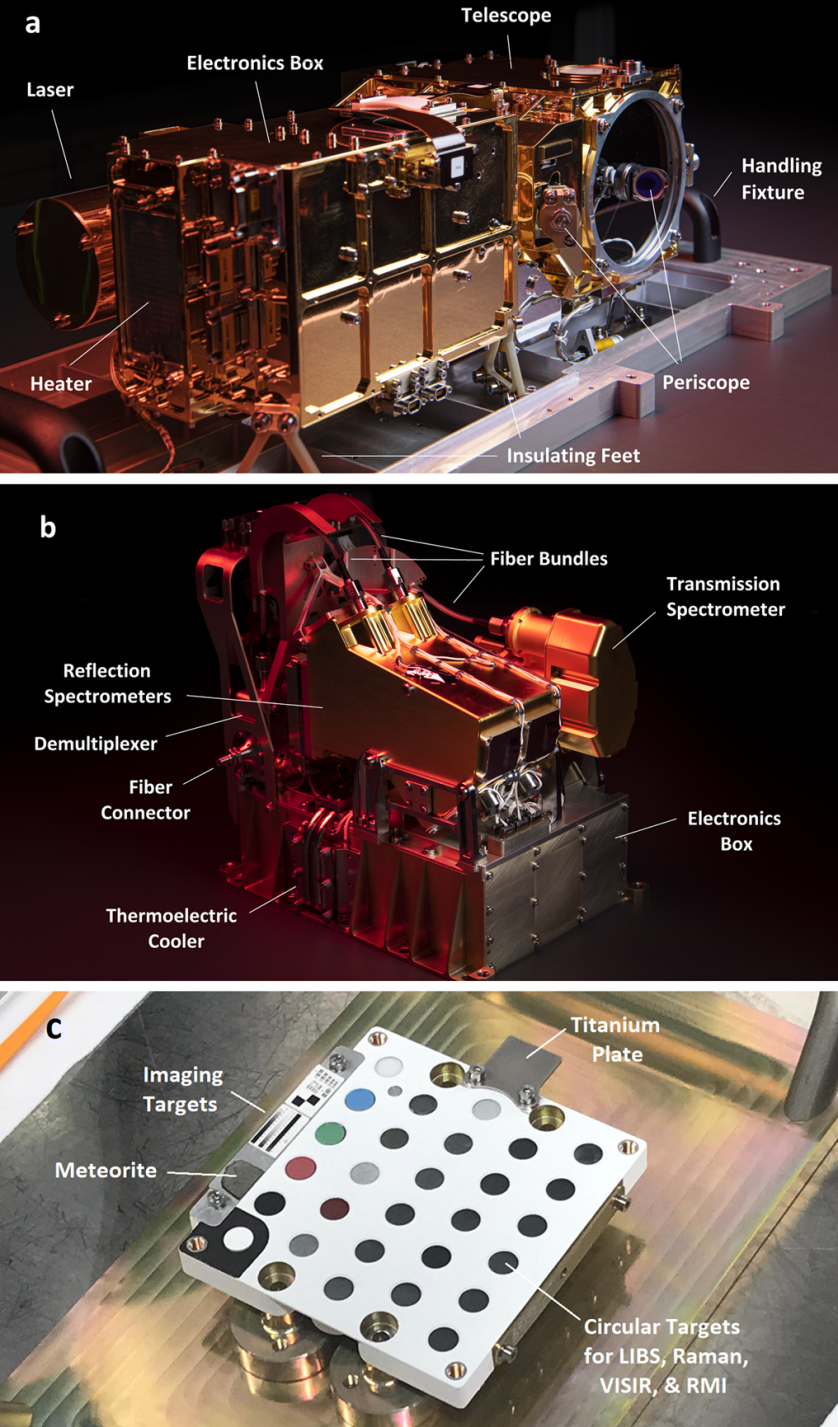
The SuperCam instrument, consisting of the Mast Unit (MU), shown in (**a**), the Body Unit (BU), shown in (**b**), and the SuperCam Calibration Target (SCCT), shown in (**c**). As part of the MU (**a**), the laser can be seen to the left, protruding from behind the electronics box. The telescope is at the far end, at the center of which the periscope mirror for the green laser beam is mounted. The corresponding periscope mirror can be seen just past the electronics box, facing away from the camera. At the near end of the electronics box, a heating pad is just to the left of the connectors. The Mast Unit is mounted on insulating feet, and is shown here resting on a handling fixture. The BU (**b**) shows a transmission spectrometer resting behind two identical reflection spectrometers, all mounted on top of the electronics box. Three optical fiber bundles can be seen with their protective shields near the upper left; these transfer light to the spectrometers from the demultiplexer. The only part of the demultiplexer that is visible is the fiber connector, protruding at the left center. This is where the light from the MU enters the BU. One of three sets of thermoelectric coolers is seen in the lower center, identified by two visible heat pipes that run under the spectrometers to cool their detectors. On the SCCT (**c**), twenty-nine circular targets and several other calibration targets are mounted. The titanium plate at the upper right is used for wavelength calibration via LIBS spectra. Imaging targets and a Mars meteorite sample line the left side. Dimensions of all three SuperCam units are given in Table 2

Description of the SuperCam instrument, shown in Fig. 1, is divided among several papers. The science goals, most of the technical requirements, and the description of the Mast Unit (MU) are in a companion paper (Maurice et al. 2020). Here we provide a description of the Body Unit (BU) and of the integrated testing prior to launch. An overview of the rover calibration targets is in another companion paper (Manrique et al. 2020). More detail on subsystems and calibrations are or will be provided in other papers published separately (Royer et al. 2020, and future papers).

## Instrument Overview

We begin with a brief overview of the entire instrument (Fig. 1) before focusing on the Body Unit. Table 1 provides a short list of the techniques employed by SuperCam.

**Table 1:** List of techniques employed by SuperCam for remote sensing. See text for explanations

Technique	Purpose	Distance^a^	Footprint
LIBS	Quantitative elemental abundances	To 7 m	0.25–0.45 mm
Raman	Identification of Raman-bright minerals, organics	To $\sim7~\text{m}$	0.74 mrad^b^
TRL	Identification of organics, minerals, REEs^c^	To $\sim7~\text{m}$	0.74 mrad
VISIR^d^	Identification of minerals; atmospheric studies	To km	VIS: 0.74 mrad IR: 1.2 mrad
RMI	Rock textures, contexts	To km	18.8 mrad
Microphone	Physical properties of rocks, atmospheric studies	To ∼4 m	N/A

^a^Minimum distance, all optical cases: 1.05 m from the MU, positioned $\sim2~\text{m}$ above flat ground

^b^mrad = milliradians

^c^REEs = rare-earth elements

^d^VIS spectral range is 0.40–0.85 μm; IR range is 1.3–2.6 μm; other ranges are given in the text

LIBS provides atomic emission spectra of material ablated from small spots on rock or soil targets, leading to quantitative elemental compositions of major, minor, and trace elements. ChemCam is able to detect and quantify $\sim25$ elements (e.g., Maurice et al. 2016), and SuperCam is expected to achieve the same as, or slightly better performance than ChemCam, with the same distance capability (Table 1). The analytical footprint is necessarily small, as the optical power density must be maximized to create a plasma on the target. The laser used to achieve the plasmas provides up to 14 mJ and $>10~\text{MW/mm}^{2}$ of 1064 nm photons per pulse (Maurice et al. 2020). As described later, the use of the transmission spectrometer in the green to red spectral range allows time gating and intensification of the signal, which may be used for special LIBS studies, such as to amplify an otherwise weak emission line.

SuperCam employs the first use of green-laser Raman spectroscopy in space, and shares the distinction of the first planetary Raman spectrometer with SHERLOC (Bhartia et al. 2020, this journal). SuperCam achieves Raman spectroscopy at remote distances to $\sim7~\text{m}$ by using a pulsed laser—the same one as for LIBS, frequency doubled to 532 nm—and an intensified, gated detector coupled to a transmission spectrometer. The green Raman laser beam is collimated rather than focused, and it overlaps with the 0.74 mrad field of view (FOV) of the spectrometer, defined by the telescope and optical fiber that transfers the light from the MU to the BU (Maurice et al. 2020). This footprint thus ranges between 1.5 mm diameter when looking straight down at the ground from the telescope’s height of 2 m, to $\sim5~\text{mm}$ diameter when observing at a distance of 7 m. Raman spectra are always dim, and collecting these spectra remotely results in a limited number of photons (Sect. 7.3.3). SuperCam’s design is a compromise to enable all of the techniques, which limited the ability to optimize the photon throughput, with the result that SuperCam observes minerals and organic materials that are strong Raman emitters. Among the best are carbonates, sulfates, and phosphates, but SuperCam also expects to identify quartz and plagioclase feldspar, the latter of which are not, or are only poorly, detected by near-infrared reflectance spectroscopy.

The pulsed laser and time-gated spectrometer also provide the capability for TRL. Prompt fluorescence is emitted by organic materials, being emitted and decaying within nanoseconds of stimulation (Lakowicz 2006). This prompt organic fluorescence is the bane of Raman spectroscopy on Earth, as even time-resolved Raman spectroscopy with nanosecond laser pulses and the fastest-gated detectors generally do not discriminate against it. However, on Mars, it may be a powerful tool for discovering concentrations of organic materials. SuperCam’s intensifier gate can be delayed up to milliseconds with a temporal resolution of 10 ns, providing a second dimension for characterizing mineral fluorescence. Using the time dimension, the presence of certain rare-earth elements (REEs) and transition metals can be identified (Gaft et al. 2015; Ollila et al. 2018).

SuperCam’s VISIR reflectance spectroscopy is the first to cover the spectral range of 0.4 to 2.6 μm from the surface of Mars. It does so utilizing several spectrometers. The main phyllosilicate identification region, in the near infrared, has been successfully used by the orbiting spectrometers, specifically the Compact Reconnaissance Imaging Spectrometer for Mars (CRISM; Murchie et al. 2007) and Observatoire pour la Mineralogie, l’Eau, les Glaces et l’Activité (OMEGA; Bibring et al. 2004). On SuperCam this spectral range is provided by a wavelength-scanning spectrometer (1.3–2.6 μm) in the MU, while observations in the VIS range (0.4–0.85 μm) take advantage of the BU spectrometers that are also used for LIBS, Raman, and TRL spectroscopies. There is a gap in the spectral coverage between 0.85 and 1.3 μm (Fig. 41 of Maurice et al. 2020). The VIS range (covered by the violet and transmission spectrometers) has a somewhat smaller target footprint than the IR spectrometer (Table 1). On Mars 2020, SuperCam’s point spectral observations are well complemented by Mastcam-Z, which can image at discrete wavelengths from 0.44 to 1.0 μm (Bell et al. 2020, this journal).

The remote micro-imager (RMI) and microphone complete the list of techniques available with SuperCam (Table 1). High-resolution context images of the analysis areas are critical to their interpretation, especially given the small footprint of the analysis techniques. The RMI provides this context via Bayer-filter color images with a resolution $\leq80~\upmu \text{rad}$ (defined as a line pair with more than 20% contrast), allowing 160 μm grains to be resolved at 2 m distance from the instrument, e.g., directly in front of the rover. (The RMI resolution is not pixel limited, as its instantaneous field of view, or IFOV, is 9.2 μrad, not considering the Bayer filter.) The microphone provides acoustic signals from the LIBS shock wave. Studies (e.g., Chide et al. 2019) have shown that a combination of rock hardness and density can be obtained by the rate of decrease of acoustic energy with increasing number of laser pulses at the same location. The microphone will also be useful for atmospheric studies (Chide et al. 2020), perhaps including phenomena of which we are not yet aware. As subsystems of the MU, the RMI and microphone are described in Maurice et al. (2020).

**Fig. 2:**
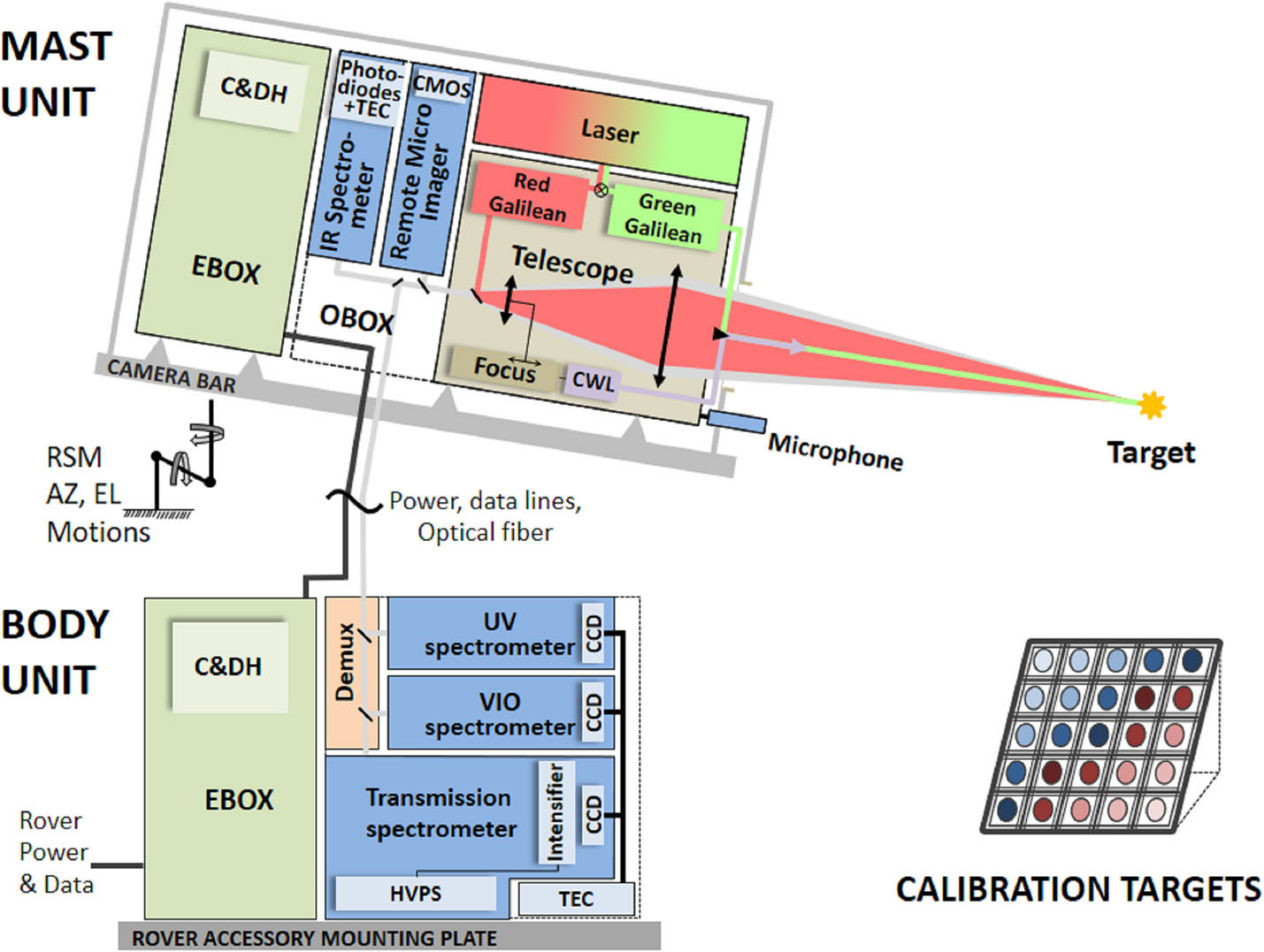
Schematic diagram showing the major units and subcomponents of the SuperCam instrument suite. The Mast Unit (MU) consists of the main laser which provides two wavelengths using two Galilean beam expanders, the telescope, a continuous-wave laser (CWL) for focusing, and a microphone. The optical box (OBOX) also includes the infrared (IR) spectrometer and the Remote Micro-Imager (RMI), the detector of which is a complementary metal oxide semiconductor (CMOS). An electronics box (EBOX) controls and powers the various subsystems in the MU. Acquisition of the target is provided by the rover mast azimuthal (AZ) and elevation (EL) motions. Electrical cables and an optical fiber connect the Body Unit (BU) to the MU. The fiber carries light in the 245–853 nm range to the demultiplexer (labeled Demux) in the BU, which distributes the light to three spectrometers covering ultraviolet (UV), violet (VIO), green, orange, and red spectral ranges. The latter are characterized by a transmission spectrometer, which uses an intensifier driven by a high-voltage power supply (HVPS). All three BU spectrometers collect light with charge-coupled devices (CCDs) cooled by thermoelectric coolers (TECs). The electronics box (EBOX) in the BU operates the instrument, provides power to the BU spectrometers and the MU, and communicates with the rover through the control and data handling (C&DH) board. A set of calibration targets is mounted on the back of the rover to facilitate calibration while on Mars

Figure 2 shows a schematic diagram of the SuperCam instrument. SuperCam is divided into two major units, the BU and the MU. The MU resides at the top of the rover’s mast and contains the laser, telescope, Remote Micro-Imager, Infrared Spectrometer, Microphone, and associated electronics. The MU was designed, built, and tested in France under the support and direction of the Centre National d’Etudes Spatiales (CNES), with integration at the Institut de Recherche en Astrophysique et Planetologie (IRAP). The BU contains an optical demultiplexer, optical spectrometers for LIBS, Raman, and passive VIS spectroscopy, and associated electronics to control the BU and MU and interface with the rover. The BU was designed, built, and tested at Los Alamos National Laboratory, in the US. A third part of the instrument, onboard calibration targets were provided by an international working group within the SuperCam team and consist mostly of sintered pellets (e.g., Montagnac et al. 2018). Characterization of the targets was performed by a team of European scientists; the Universidad de Valladolid was responsible for integration and environmental testing of the target assembly (Manrique et al. 2020).

**Fig. 3:**
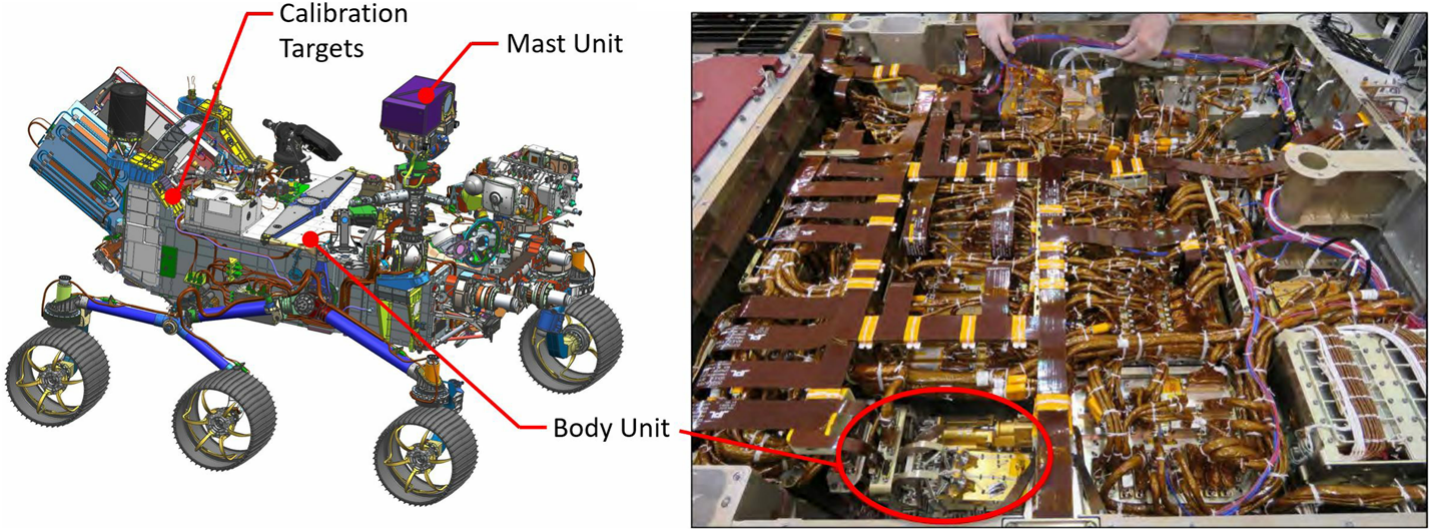
Locations of the SuperCam units on the rover. The right side shows the rover body inverted, with the Body Unit circled. It is next to the RSM side of the rover to minimize the length of the fiber that transfers the optical signal from the mast unit. The rover’s instrument and electronics bay is $1181 \times 1106~\text{mm}$ (length, left-right, x width)

Figure 1 shows the completed SuperCam instrument, which was designed to conform to the space provided by the rover project (Fig. 3). The available volume was quite similar to that of ChemCam (Maurice et al., Wiens et al. 2012), and so from both heritage and mechanical/thermal-requirement standpoints, SuperCam looks very similar to its predecessor. SuperCam’s telescope differs from ChemCam’s, as the newer instrument features a periscope mirror that directs Raman laser light to the intended sample (Fig. 1). The BU’s optical demultiplexer and two reflection spectrometers—ultraviolet and violet (UV, VIO)—are nearly identical to ChemCam’s, but its third reflection spectrometer was replaced by a time-gated, intensified, high-throughput transmission spectrometer to enable Raman spectroscopy. The calibration target assembly (Fig. 1; Manrique et al. 2020) is vastly expanded and improved over ChemCam’s (Fabre et al. 2011; Vaniman et al. 2012; Wiens et al. 2012).

**Fig. 4:**
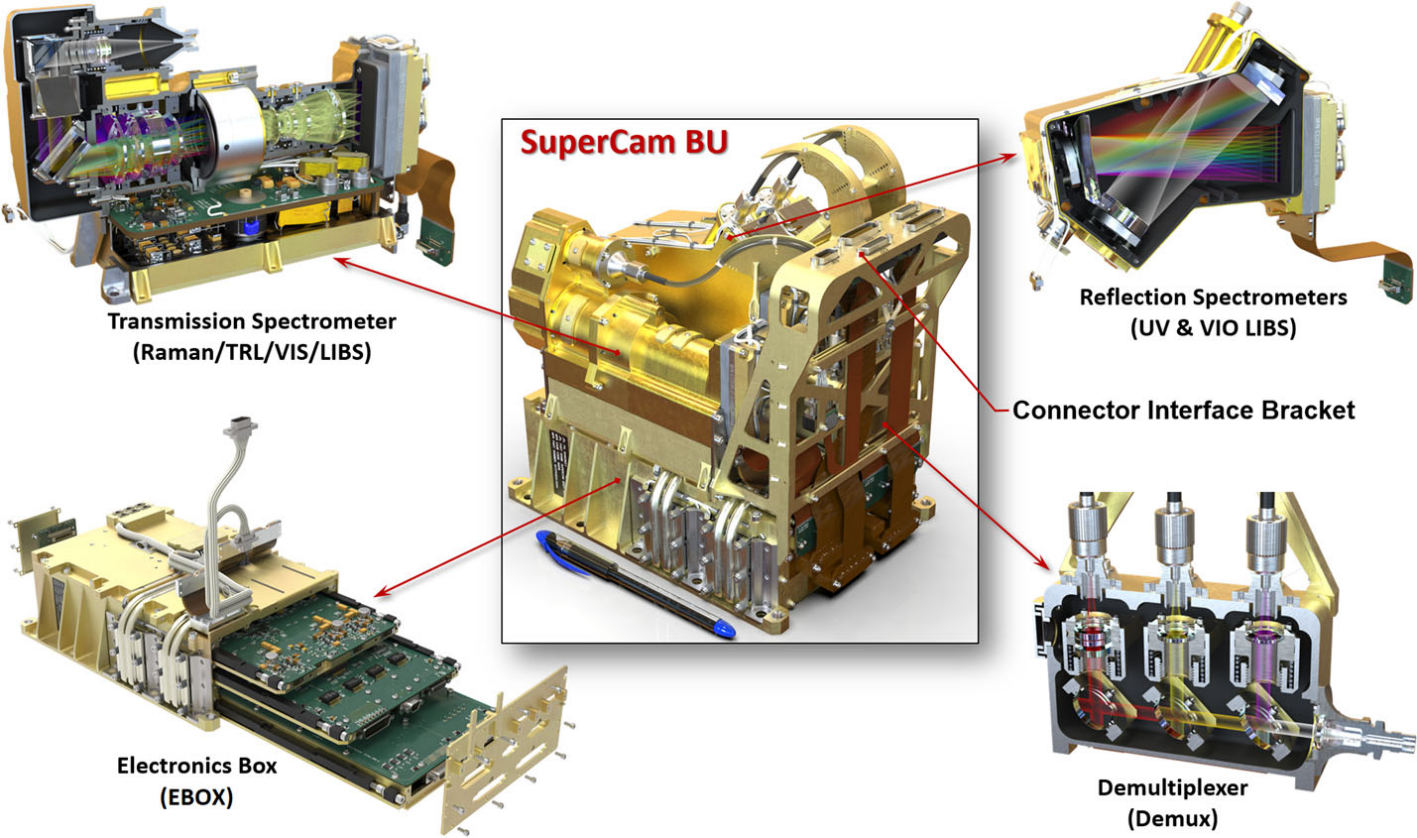
Subassemblies of the Body Unit

Table 2 presents some of SuperCam’s physical properties. Overall, SuperCam has almost exactly the same mass as ChemCam. The MU and calibration targets are slightly heavier, but the BU is lighter. The difference is largely due to ChemCam’s thermoelectric cooler (TEC) assembly, which—thanks to a JPL team–was added to that instrument within the final 18 months of its development, when mass was not a concern (Wiens et al. 2012). The TECs on SuperCam were planned from the beginning, and so the design of the associated cooling system is lighter. Another difference affecting the mass of SuperCam is the replacement of beryllium by titanium for the BU spectrometers. Thermal expansion of ChemCam’s spectrometers requires careful compensation for changes in the wavelength calibration with respect to the instantaneous temperature of the spectrometers on Mars, which change by $20~^{\circ}\text{C}$ diurnally. Titanium has a lower coefficient of thermal expansion (CTE) than Be, so maintaining wavelength calibration will be easier. To fit Ti spectrometers into the tight mass budget, the mechanical design of the reflection spectrometers was changed, while the optical design and mounting of optical elements remained the same. The overall result was very slightly heavier spectrometers (the reflection spectrometers total $\sim0.85~\text{kg}$), but within the mass budget. Another change to the BU was the addition of a connector interface bracket (left side of Fig. 1b, and Fig. 4). Space around the BU in the rover was heavily subscribed (Fig. 3), and the bracket made the electrical connections more accessible.

**Table 2:** SuperCam physical properties

	Mass (kg)	Dimensions *l* × *w* × *h* (mm)	Max power (W)
Mast Unit	6.11	383 × 201 × 163	27
Body Unit	4.44	221 × 157 × 205	43^a^
Cal. Target	0.25	110 × 100 × 17	0
TOTAL	10.80		70

^a^Includes operation of the thermoelectric coolers. BU power when idling is 12 W

Figure 3 shows the locations of the SuperCam units within the rover. The BU is mounted upside down (relative to Fig. 1), hanging from the Rover Accessory Mounting Plate (RAMP). The RAMP heats the instrument and the rest of the rover body via the thermal loop which circulates fluid through the radiothermal isotope generator (RTG). The RAMP also removes excess heat from SuperCam’s TECs and its BU electronics. The location of the BU is slightly behind the Remote Sensing Mast (RSM), a position selected to minimize the length of the fiber optic cable (FOC) that carries photons to the BU from the telescope in the MU. The MU is housed in the remote warm electronics box (RWEB), a white box at the top of the rover’s RSM. The RWEB is made of thin aluminum, with a window for the telescope. Survival temperature of $> -40~^{\circ}\text{C}$ in the RWEB is maintained by heaters mounted on the instrument. Separate operational heaters warm parts of the MU for operation, and control their cooling rate (Maurice et al. 2020). The MU is $\sim2~\text{m}$ above ground level, so SuperCam’s closest observations on the Mars surface are at $\sim2~\text{m}$ distance. The SuperCam Calibration Target assembly (SCCT; Fig. 1c) is mounted at the back of the rover (Fig. 3, left). The targets are between 1.541 and 1.567 m from the MU at RSM elevation angles between −27.15 and $-29.91^{\circ}$. The SCCT is mounted at an angle of $50^{\circ}$ relative to horizontal (when the rover is on a level surface).

## Body Unit

Figure 4 shows the sub-assemblies of the BU. We will start with an optical description of the BU, followed by mechanical and thermal, and finishing with the electrical and software descriptions.

### Optical Description

**Fig. 5:**
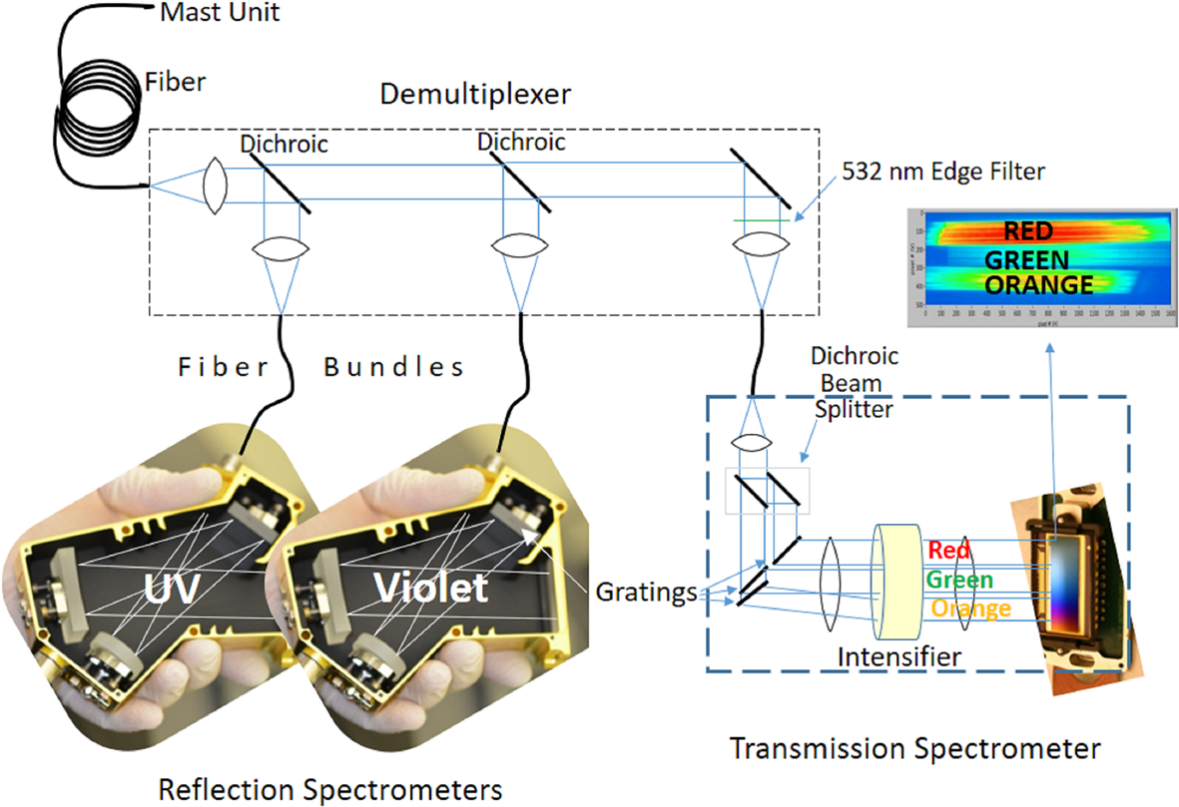
Schematic diagram of the optical layout of the SuperCam BU. The Fiber Optic Cable (FOC) transfers light from the MU to the demultiplexer, which uses two dichroic mirrors to split the light into three bands. An edge filter removes green laser light from the Raman signal. Fiber bundles transmit the light from the demultiplexer to the spectrometers. The ultraviolet (UV) and violet bands are dispersed and recorded by crossed Czerny-Turner reflection spectrometers of identical design but with their own mirrors and gratings. They are shown prior to CCD installation. The transmission spectrometer separates a red band using a dichroic beam splitter, and it separates green and orange bands using a compound grating. All three bands are focused onto the intensifier. Beyond the intensifier, a set of relay lenses re-focuses the light onto the CCD, which collects all three bands of light. For illustration purposes, the bands are shown orthogonal to the way in which they are actually projected onto the intensifier and CCD; the correct orientation is illustrated above, indicated by the blue arrow

All three BU spectrometers (Fig. 4) are used for LIBS. Raman and TRL spectra are generated by the transmission spectrometer. Passive VIS reflectance spectra are provided by the VIO and transmission spectrometers. The spectral ranges of each spectrometer, the techniques for which they are used, and other details are given in Table 3. Figure 5 provides an overview of the optical design of the SuperCam BU. The fiber optic cable (FOC) transfers light for LIBS, Raman, TRL, and VIS spectroscopies from the telescope in the MU to the demultiplexer in the BU. The demultiplexer efficiently splits light into three spectral bands, coupling the light into fiber bundles that contain the aperture slits of the individual spectrometers. The FOC will be described first, then the demultiplexer and spectrometers.

**Table 3:** Optical characteristics of the spectrometers and their detectors

	Ultraviolet (UV)	Violet (VIO)	Transmission
Techniques	LIBS	LIBS, VIS	LIBS, Raman, TRL, VIS
Spectrometer type	- - - Crossed Czerny-Turner - - -		Transmission
Spectral range	243.5–341.7 nm	382.1–467.5 nm	535–620 nm, green
			620–712 nm, orange
			712–853 nm, red
Pixel resolution	0.0478 nm/pixel	0.0417 nm/pixel	0.064–0.090 nm/pixel
Channels	2048	2048	2048 × 3
Vertical integration rows	200: 151–351	200: 151–351	Red: 91–161; 21–221^a^
	40: 232–272	40: 236–276	Green: 236–356
	16: 252–268	16: 248–264	Orange: 378–506
CCD coating	Enhanced UV	Enhanced Broadband	Enhanced Midband
CCD QE^b^	77.2% @ 250 nm	81.3% @ 345 nm	92.8% @ 635 nm
	64.9% @ 300 nm		
	55.4% @ 345 nm		
CCD dark current (e-/pix/sec @ 300 K)	269	202	224
Photon response non-uniformity (PRNU)	4.5% @ 350 nm	0.7% @ 400 nm	0.7% @ 650 nm
Full well, single pixel	123k e^−^	128k e^−^	128k e^−^
Full well serial reg.	190k e^−^	192k e^−^	192k e^−^
Conversion gain (e-/DN^c^)	3.14 ± 0.06	3.16 ± 0.05	3.15 ± 0.06

^a^QE, dark currents, and PRNU were measured by the CCD manufacturer

^b^Red region has options of 70 or 200 row integrations

^c^Digital numbers, also known as analog-to-digital units (ADU), or counts

#### Mast-to-Body Fiber Optic Cable

**Fig. 6:**
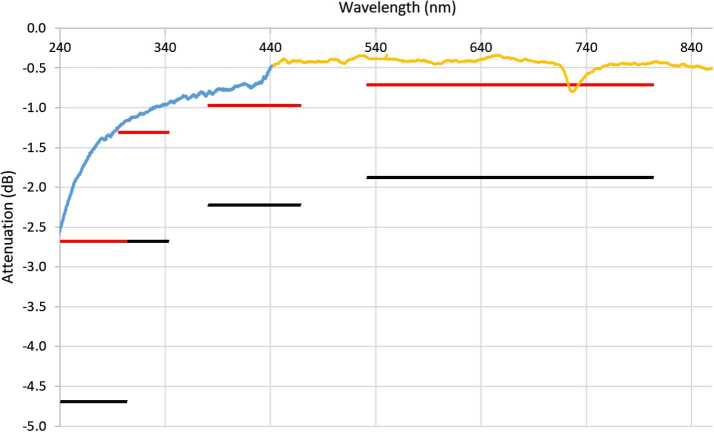
Attenuation and insertion loss for the flight fiber-optic cable, measured with two commercial spectrometers (orange and blue curves), made after planetary protection/contamination control bake-out and flight-acceptance thermal cycling. Red and black lines indicate goal and requirement levels, respectively

The FOC is a Polymicro fiber with 0.22 numerical aperture (NA), 300/330 μm diameter core/cladding, and is 5.78 m long, with AVIM connectors on both ends. The FOC is essentially identical to the one from the Mars Science Laboratory (MSL) rover (Wiens et al. 2012), and in fact, the SuperCam proposal offered to use flight spare units from MSL. Although several were available, it was decided to use new fiber that was cut, attached to connectors, and inspected at the terminations by a team at Goddard Space Flight Center. Figure 6 shows the performance of the fiber against requirements. The dip near 730 nm is thought to be due to an OH absorption; it was also observed with the ChemCam fiber.

The fiber is routed from the MU down the mast through two twist caps that are co-axial with the RSM azimuth and elevation gimbals. The fiber makes three rotations around each axis in the two twist caps, which ensure that the fiber stays above its minimum bend radius even when the gimbal is fully rotated in any direction. At the base of the RSM, the fiber traverses the rover deck before entering the rover body near the BU. The routing and packaging of the fiber, and the design of the twist caps, are nearly unchanged from MSL. Testing of the MSL RSM gimbals was done to verify their robustness. An RSM gimbal with a representative FOC was tested over 16500 cycles in a clean configuration at temperatures of −110 and $+70~^{\circ}\text{C}$, and an additional 16500 cycles in a dusty environment, with no degradation. The test was judged to be applicable for the SuperCam fiber as well. In 2018 it was noticed that the MSL FOC had slipped out of its place in the elevation mandrel. Inspection of images taken over the years since installation on the rover around 2010 indicated that the fiber had slipped out of place before launch; however, no degradation due to its current position has been observed to date. A slight change was made to the Mars 2020 mandrel to avoid this issue with SuperCam.

#### Demultiplexer and Fiber Bundles

**Fig. 7:**
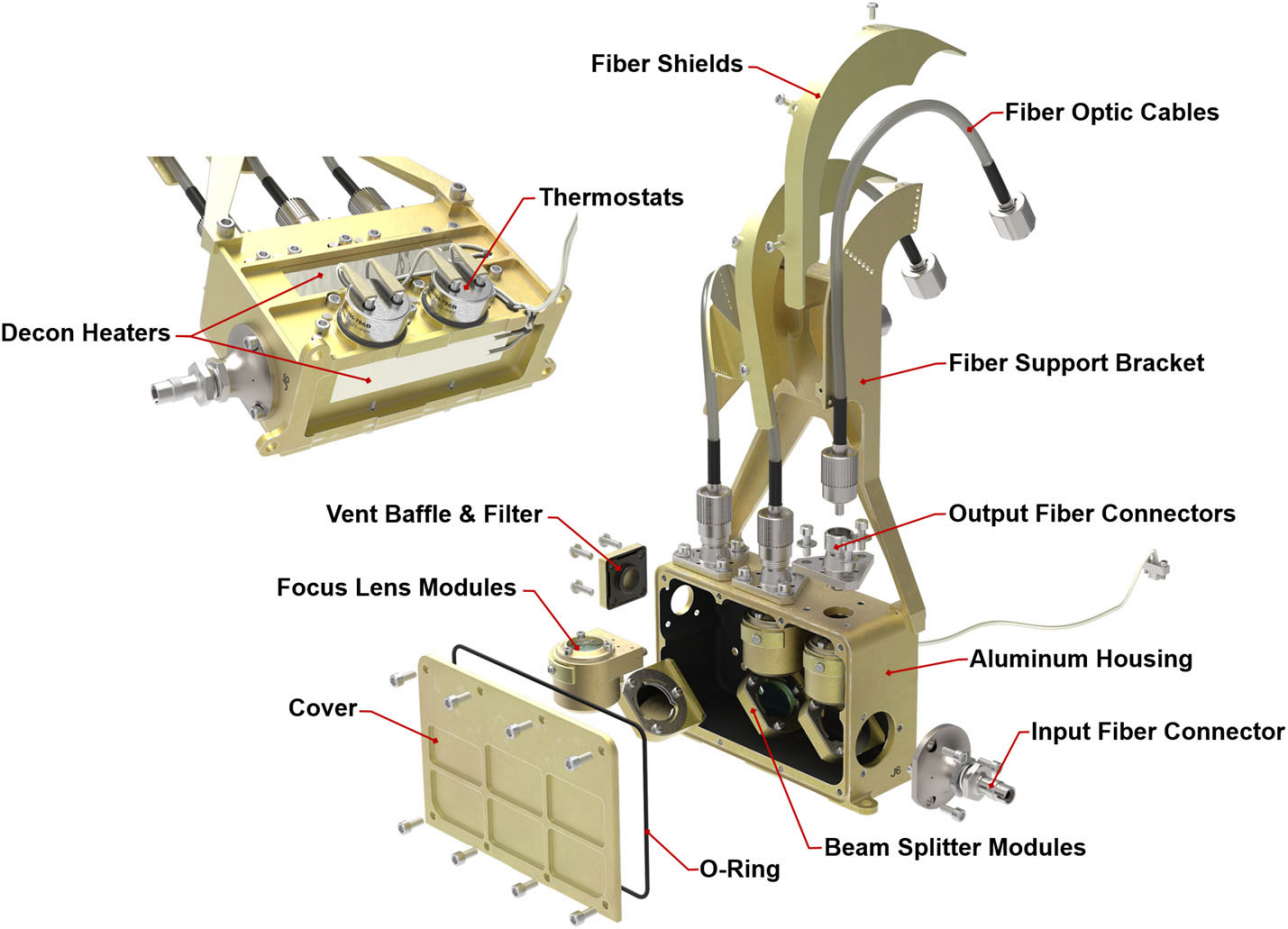
Rendering of the optical demultiplexer which accepts light from the FOC (at the connector at the right) and distributes it efficiently to the three spectrometers through three fiber bundles

The demultiplexer (Fig. 5, top; Fig. 7) consists of an input fiber connector for the FOC, a collimating lens, two dichroic mirrors to reflect successively the UV and violet bands for their respective spectrometers, and a third mirror for the remaining light. These are housed in beam splitter modules (Fig. 7). The mirrors were produced by Materion, with requirements for the UV to reflect $>90\%$ from 240–340 nm and transmit $>95\%$ average from 385–930 nm; the VIO mirror was required to reflect $>90\%$ from 385–460 nm and transmit $>95\%$ from 500–930 nm, including a back-side anti-reflection coating. The final mirror is a dielectric with $>95\%$ reflection from 500–930 nm. The optics are for random polarization. The last band of the demultiplexer includes a Semrock standard E-grade edge (high-pass) filter (Fig. 5), with a $90~\text{cm}^{-1}$ transition to remove the laser light at 532 nm. It is part of a two-filter system in which a Materion notch filter in the MU removes 95% of the light at 532 nm (Maurice et al. 2020) to minimize Raman excitation of the silica in the FOC, which had been observed in the first model of SuperCam, which did not contain this laser-rejection filter. Because this filter in the MU must transmit light at wavelengths $<465~\text{nm}$ as well as $>535~\text{nm}$, it was not feasible to construct a perfect barrier at 532 nm. The edge filter in the demultiplexer rejects the remaining laser light with an extinction ratio greater than 40 dB.

**Fig. 8:**
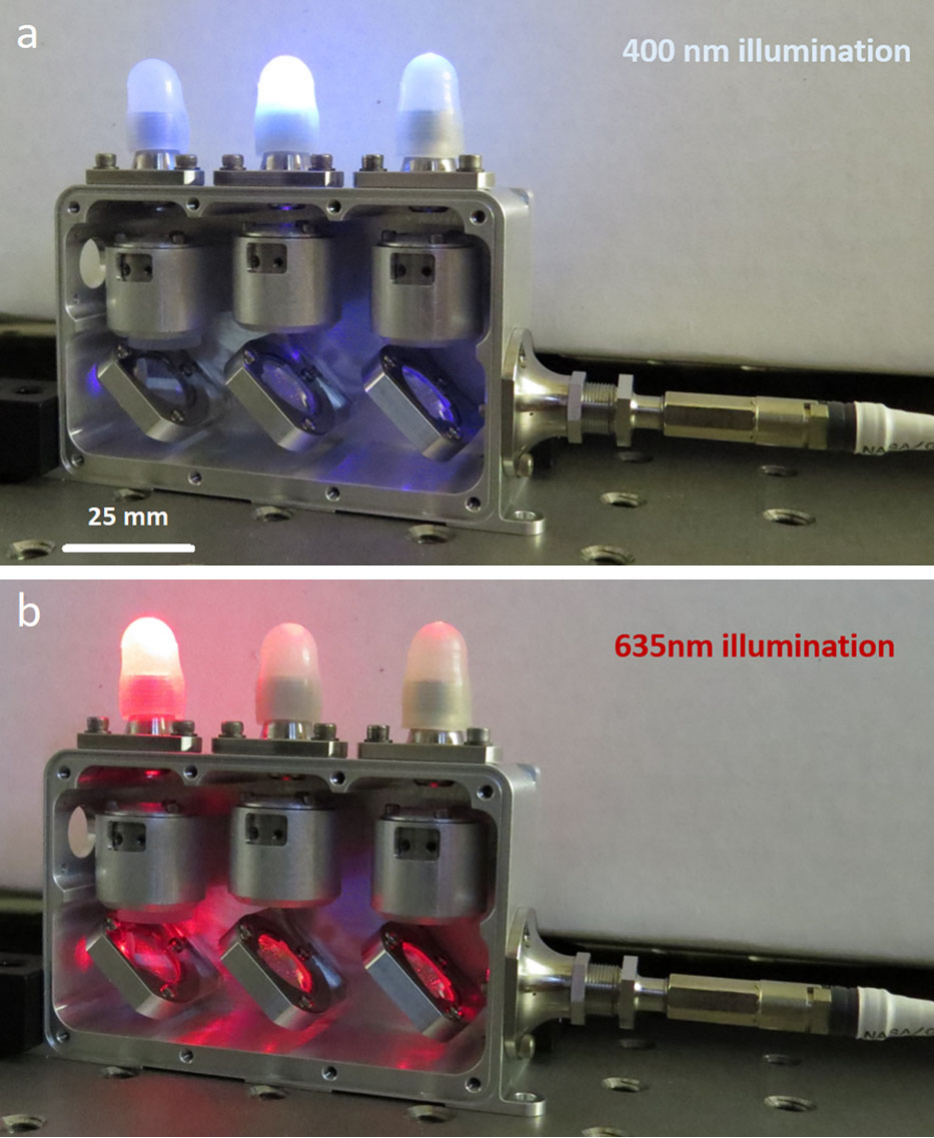
Demultiplexer illuminated with 400 (**a**) and 635 (**b**) nm light, showing the separation of wavelength bands for the VIO (**a**) and transmission (**b**) spectrometers. UV illumination of the third band on the right side is invisible to the human eye, so it is not shown. An approximate scale is indicated in (**a**). The table holes are centered 25.4 mm apart

All three demultiplexer bands have custom coupling lenses (Fig. 7), and adjustable fiber mounts for the output light. Starting with the general ChemCam design, Zemax modeling software was used to redesign the lenses in the demultiplexer. Each lens design was optimized for coupling efficiency of each wavelength band into its output fiber bundle. The design was iterated to optimize it to an incoming light cone with a NA of 0.15 received from the MU and adapt to $\text{NA} = 0.12$ for the spectrometers. Figure 8 illustrates the separation of light into wavelength bands.

Illumination provided by the FOC is assumed to be uniform in angle space, with half-angle given by the NA. The output fiber bundle is modeled as a single fiber with 280 μm diameter. It is actually 19 fibers with 50 μm diameter core and a 55 μm cladding. The difference is a fixed loss, independent of the input NA, so we leave it out of this analysis. “Coupling” means rays land on a circle of 280 μm diameter with an incident angle not greater than $\pm6.9$ degrees, which is the largest angle accepted by the spectrometers. This corresponds to $\text{NA}= 0.12$ which is f/4. All rays in the collimated section were constrained to 6.35 mm diameter clear aperture. The demultiplexer receives light at

$$ A \Omega = \pi (150~\upmu \text{m})^{2} (\text{NA})^{2} $$

where $A\Omega $ is the étendue. The demultiplexer couples light out at

$$ A \Omega = \pi (140~\upmu \text{m})^{2} (0.12)^{2} $$

So we expect the efficiency to go as

$$ \eta = \frac{0.0125}{\text{NA}^{2}} $$

No matter how good the lenses inside are, it is not possible to perform better within the angle that the rays must subtend to couple to the fiber. The results of both the modeling and post-alignment measurements are shown in Table 4. The measurements were made with a 300 μm output fiber, and scaled to correspond to a 280 μm fiber for comparison with the optical model.

**Table 4:** Modeled theoretical maximum transmission through the demultiplexer, and measured transmissions for various wavelengths

Spectrometer	% Transmission @ Wavelength (nm)					Theoretical maximum
	300	405	565	660	780	
UV	49	–	–	–	–	59
VIO	–	55	–	–	–	59
Transmission	–	–	63	61	58	58

ChemCam was optimized for transmission of the UV range; performance in the spectral range $> 500~\text{nm}$ (green to red bands) was below the étendue limit. Designing the demultiplexer for $\text{NA} = 0.15$ improved the efficiency in the transmission spectrometer up to the étendue limit. This is a slight degradation for UV and VIO ($65\% \rightarrow 59\%$), and a significant improvement for green to red range ($43\% \rightarrow 58\%$). Note that measured throughput in the transmission-spectrometer channel exceeds the theoretical maximum: We believe this is because the model assumes the input illumination is uniform in angle and spatial extent, whereas the true distribution is likely somewhat brighter in the center.

**Fig. 9:**
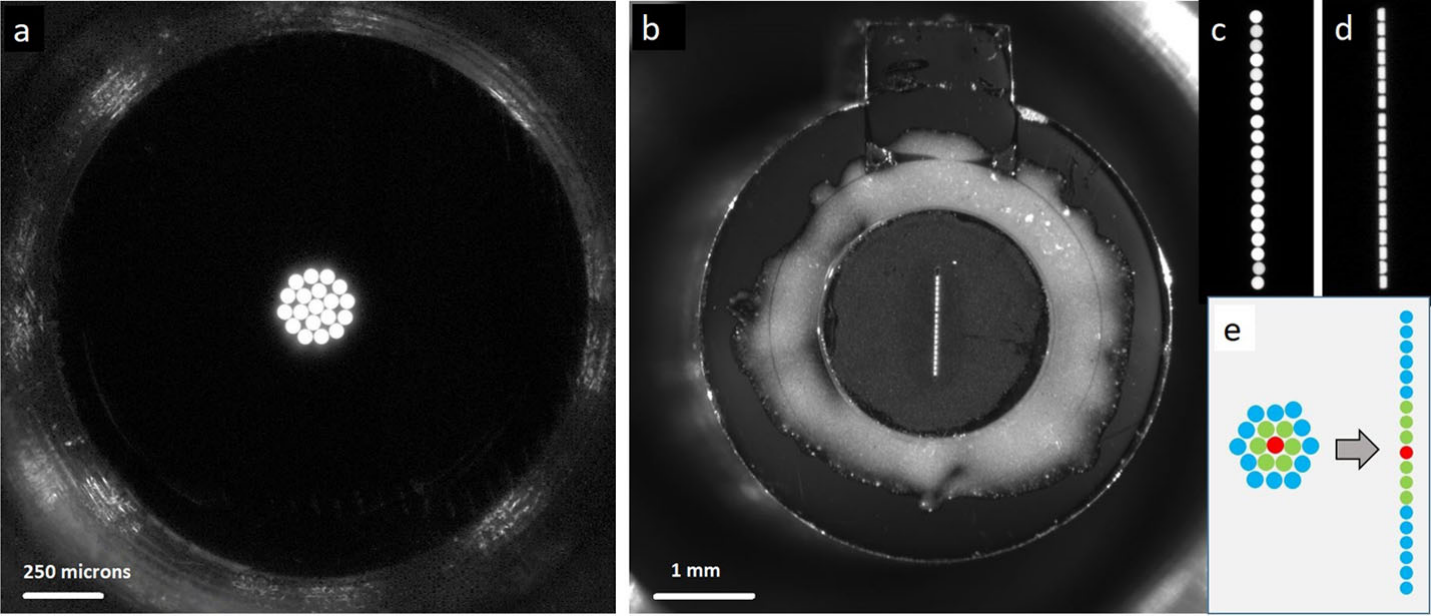
Typical inspection images of demultiplexer end (**a**) and spectrometer end (**b**) of fiber bundles, showing the one feeding the transmission spectrometer. The core of each fiber is 50 μm diameter. Fibers are backlit for the inspections. The relative intensity of each fiber may be a function of the position of the lamp, and so does not indicate relative throughput in this case. Inset shows a magnified image of the linear array of fibers at the spectrometer end before (**c**) and after (**d**) bonding a 28.7 μm slit. Inset (**e**) shows the mapping of fibers in the array, with central fibers in the circle mapped to central fibers in the line

The fiber bundles consist of nineteen individual high-OH fibers manufactured by PolyMicro, which were fabricated into bundles by FiberTech Optica. The bundles are 184 mm length, with custom keyed connectors on the spectrometer end. As shown in Fig. 9, the circular end, which accepts light from the demultiplexer, is a closest-packed arrangement of a single fiber at the center surrounded by six fibers, with twelve fibers forming the perimeter. The fibers are mapped to the spectrometer end, such that the seven center fibers in the demultiplexer end are at the center of a linear arrangement of fibers (Fig. 9e). The bundles are packaged in protective tubing that is flexible enough to permit a bend between the demultiplexer and the spectrometers. The fiber bundles were inspected both at the manufacturer and at LANL for functionality and throughput, and to ensure proper terminations, circularity and linearity of the arrangements, and mapping from one end to the other.

After characterization, slits procured from National Aperture were installed on the best bundles. The slits are 1.16 mm long, produced in 13 μm thick plates that were blackened. The slits were inspected and measured at LANL prior to installation, and performance was checked after installation. The slit widths used in each spectrometer are given in Table 5, along with the measured optical efficiency of the completed fiber assemblies with slits attached. Note that the reported transmission values include losses both from the slit (given under “Theoretical maximum”) and the losses from the packing fraction of the nineteen 50 μm fibers.

**Table 5:** Modeled theoretical maximum transmission through the fiber bundle and slit assemblies, and measured transmissions for various wavelengths

Spectrometer	Slit width (μm)	% Transmission @ Wavelength (nm)					Theoretical maximum
		300	405	565	660	780	
UV	20.7	23	42	42	44	36	51
VIO	21.0	22	42	42	42	35	52
Transmission	28.7	32	51	53	53	46	69

**Fig. 10:**
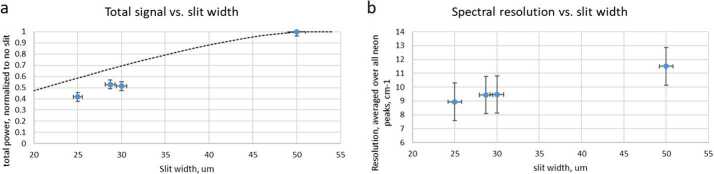
Comparison of optical throughput efficiency (**a**) and spectral resolution (**b**) as functions of entrance slit width for transmission spectrometer. The theoretical maximum optical transmission is shown as a dotted line in (**a**). As expected, a wider slit allows more light but also increases the FWHM of spectral features. The 28.7 micron slit was selected for flight. Although not shown in the figure (the lowest-wavelength Ne line used was at 576 nm), this slit just meets the $12~\text{cm}^{-1}$ resolution requirement at the short-wavenumber end of the spectrum

As with any spectrometer, selecting a slit width involves a trade between spectral resolving power and optical efficiency. To ensure the optimal choice, we built and tested fiber bundle assemblies with several slit widths and on each we measured the optical throughput and resolving power, defined as the average full width at half maximum (FWHM) of several atomic emission lines from a neon lamp. The results, seen for the transmission spectrometer in Fig. 10, led us to select the 28.7 μm wide slit assembly for flight. The neon emission lines that were used do not cover the most challenging spectral region, from 535–555 nm, where spectral resolution is worse. With this slit, the transmission spectrometer just meets its FWHM resolution requirement of $12~\text{cm}^{-1}$ everywhere. Similar testing led to the selection of the 20.7 and 21.0 μm slits for the UV and VIO spectrometers, respectively (Table 5).

#### Reflection Spectrometers

**Fig. 11:**
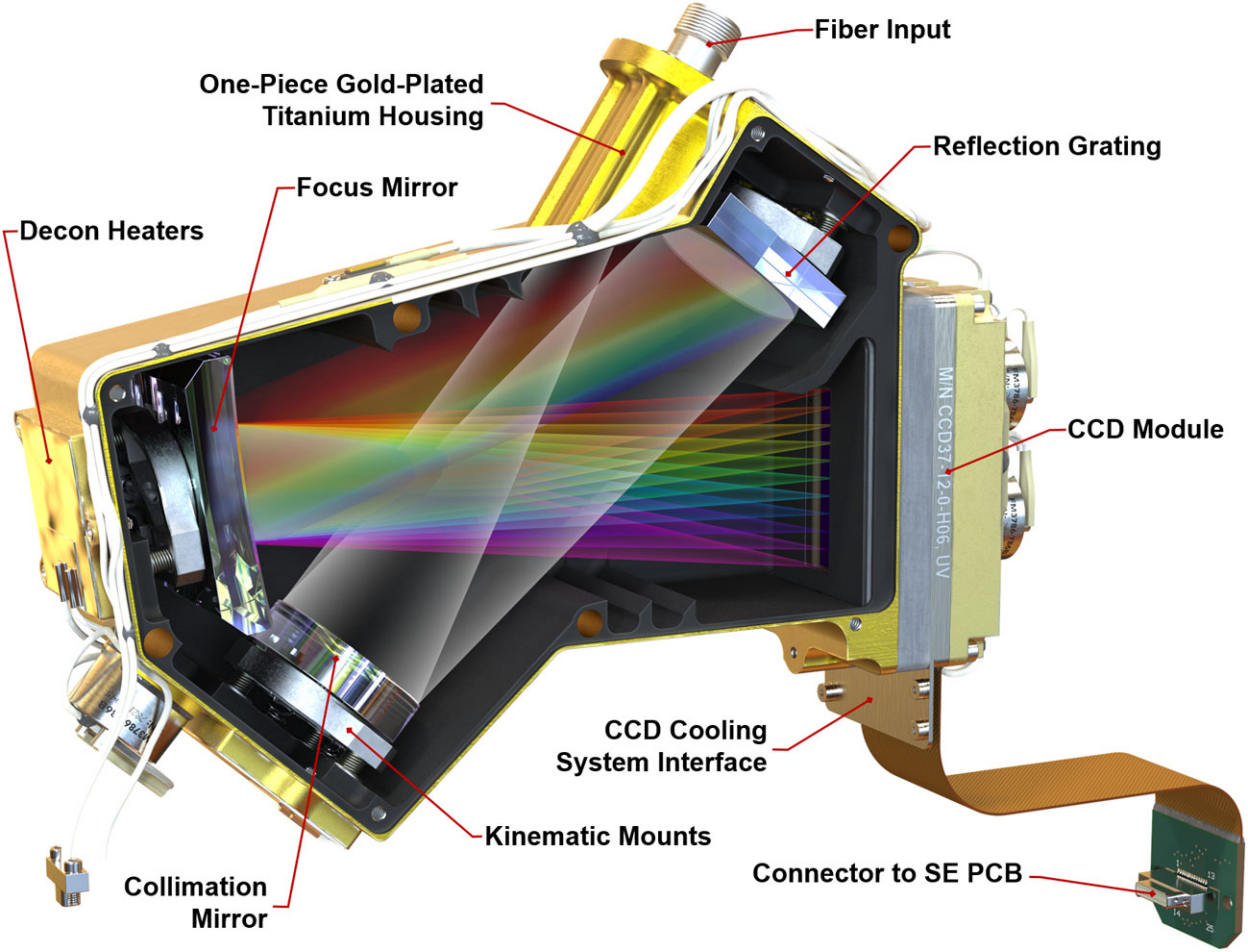
Rendering of a SuperCam reflection spectrometer. Light enters from the fiber bundle and slit assembly at the upper right. It is collimated by the circular mirror at lower left. The grating at upper right provides spectral dispersion, the first order of which is focused by the rectangular mirror at the upper left onto the detector assembly at the lower right. Baffles can be seen along the upper and lower sides and next to the grating, used to absorb higher order reflections from the grating. The cylinder protruding at the left is a thermal switch

As shown in Fig. 5, two of the three spectrometers are crossed Czerny-Turner reflection spectrometers. Table 3 gives specifications in terms of wavelength range and resolution of the spectrometers. The f/4 optical design is essentially identical to that of ChemCam (Wiens et al. 2012). The two spectrometers are identical to each other with the exception of the mirrors, gratings, and grating angles. Figure 11 shows a rendering of the interior of the reflection spectrometers. The gratings, manufactured by Richardson, are 2400 lines per mm (lpmm) on Schott N-SF8 glass substrates with aluminum coatings. The UV grating is 240 nm blaze wavelength, while the VIO grating is 300 nm. The mirrors have spherical concave surfaces with 100 mm radii of curvature, with dielectric surfaces for maximum reflectance in the respective spectral ranges. Mirrors were manufactured by OPCO, and have reflectivity $>94\%$ between 240–340 nm for the UV spectrometer, and $>98\%$ between 380–500 nm for the VIO spectrometer.

The diffracted images of the spectrometer slits are projected onto charge-coupled devices (CCDs). All three spectrometers have identical CCD assemblies with the exception of the CCD surface coatings, which were selected to optimize light collection at the respective ranges (Table 3). As with ChemCam, they are E2V 42-10 CCDs, 13.5 μm square pixels in an array of $2048 \times 515$ with 50 additional blind serial-register pixels on each side. The active area is $27.6 \times 6.9~\text{mm}$. Extensive testing was carried out on the CCDs and on replicates from the same factory lot. In addition to testing and characterization at the manufacturer (Table 3), read noise, dark current, image and serial pixel well capacities, and sensitivity to input voltages were all tested at LANL, and the best among several CCDs of each type were assigned for the flight instrument. A blemish was found in the flight VIO CCD after integration onto the CCD board, in which the sensitivities of pixels in rows between 240 and 250 at columns 1323–1326 are significantly reduced. This was not noted by the manufacturer.

**Fig. 12:**
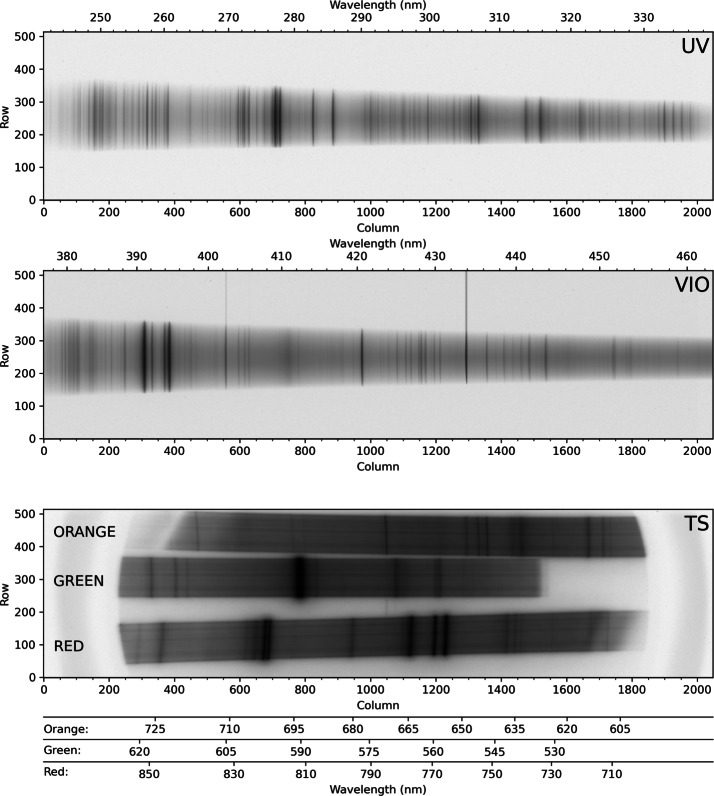
LIBS light from andesite standard JA-3 (shot in terrestrial atmosphere) as seen by SuperCam’s detectors. Shown are full images of the CCDs of UV, violet (VIO), and transmission (TS) spectrometers. These images are shaded as the natural logarithm of intensity. Rows and columns are indicated, as are the wavelengths. Two lines extending to the top of the VIO image are Hg emission lines from room lights. In the transmission spectrometer, the edge of the intensifier can be perceived by the edges of the continuum emission

The LIBS spectral image from andesite standard JA-3 is shown in Fig. 12. In the reflectance spectrometers, the light is spread across up to 180 central rows. (The VIO blemish is not seen.) ChemCam integrates the signal from 200 vertical rows to maximize the collection (Wiens et al. 2012). For SuperCam we plan to use several different vertical integration-row settings on the reflection spectrometers (Table 3). Operation of the SuperCam CCDs and their timing with respect to the laser are discussed in Sects. 3.3.1 and 4.2.

#### Transmission Spectrometer

The transmission spectrometer is a significant innovation relative to ChemCam; it enables the remote Raman and TRL spectroscopy techniques, while providing enhanced capabilities for LIBS and also collecting passive VIS reflectance spectra. The primary functions are to provide high transmission and intensification at relatively high resolution for the weak Raman signal, and to provide time gating to 100 ns to minimize noise from background ambient light and fluorescence. The transmission spectrometer design is based very loosely on earlier commercial Raman transmission spectrometers such as the Kaiser Holospec, but with a long separate development period, first at U. Hawaii with modified commercial parts (e.g., Sharma 2007) and then at LANL with custom parts, starting around 2009.

**Fig. 13:**
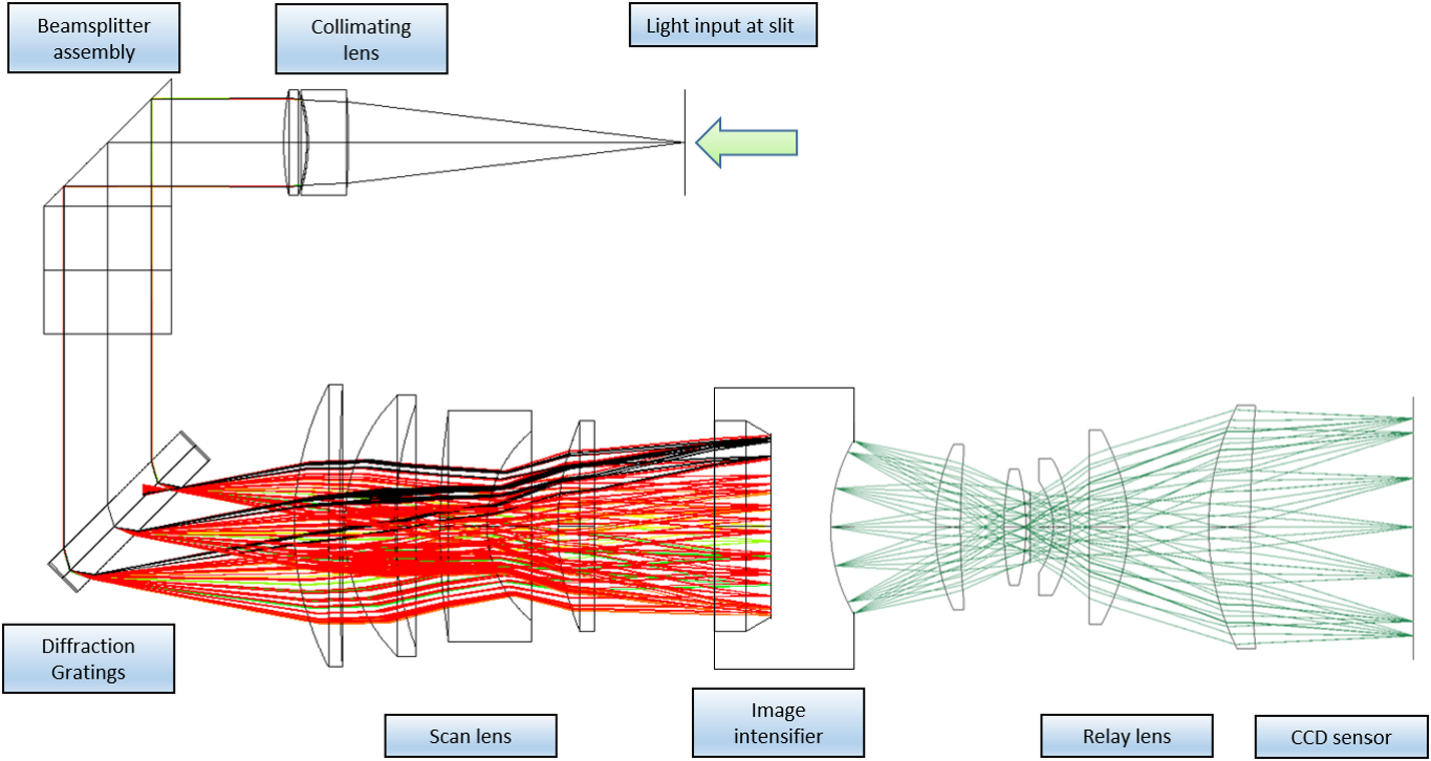
Ray traces in a model of the transmission spectrometer, showing collimation, diffraction, and formation of slit images on the image intensifier entrance window and relay lens carrying intensified image to CCD sensor. Rays are colored by wavelength; 534 nm to 853 nm to the intensifier, and 545 nm from the intensifier phosphor to the CCD

Figure 5 shows the basic architecture of the spectrometer, while Fig. 13 shows an optical ray trace. Three diffracted bands are used to simultaneously cover the wavelength range required for LIBS (535–853 nm) while achieving the resolution needed for planetary green-laser Raman spectroscopy ($12~\text{cm}^{-1}$) over its range ($\sim105$ to $4000~\text{cm}^{-1}$; 535–676 nm). Because the LIBS spectral resolution and light collection requirements are not as stringent in this spectral range, the transmission spectrometer was optimized over the narrower Raman range while still covering the broader LIBS spectral range. The feature that limits the resolution is the intensifier rather than the CCD.

**Fig. 14:**
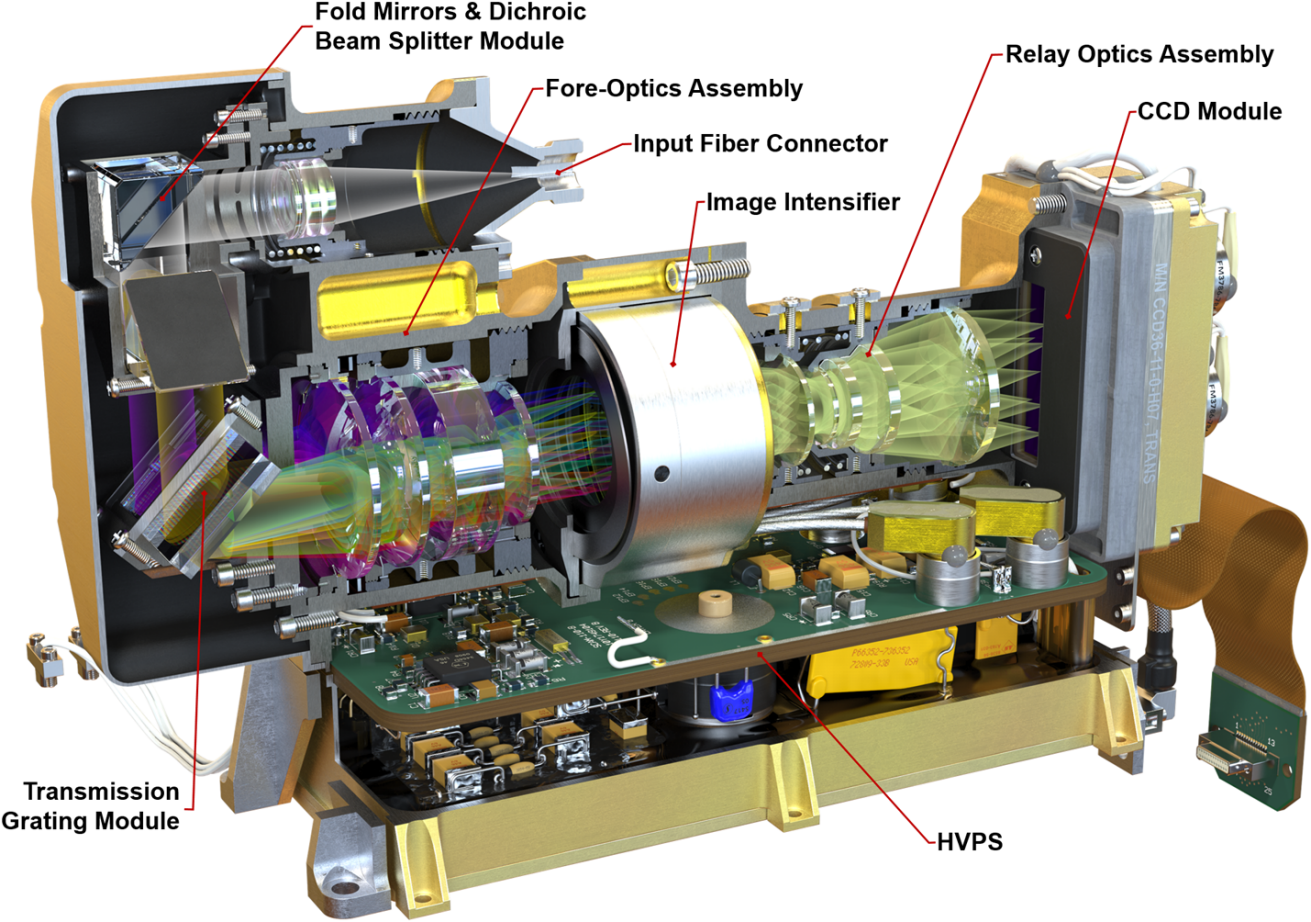
Cutaway rendering of the transmission spectrometer showing the internal layout

As shown in Fig. 5 and Fig. 13, light passes through the slit and is collimated before encountering a dichroic beam splitter assembly, which separates a red optical band. Two parallel beams travel to separate transmission gratings. A compound grating (one directly behind the other, with an angular separation) separately diffracts green and orange light, while an adjacent grating diffracts the red band. All three bands are focused on the intensifier. As a final step, intensified light is re-focused on the CCD in the relay section. A rendering of the spectrometer is shown in Fig. 14.

**Fig. 15:**
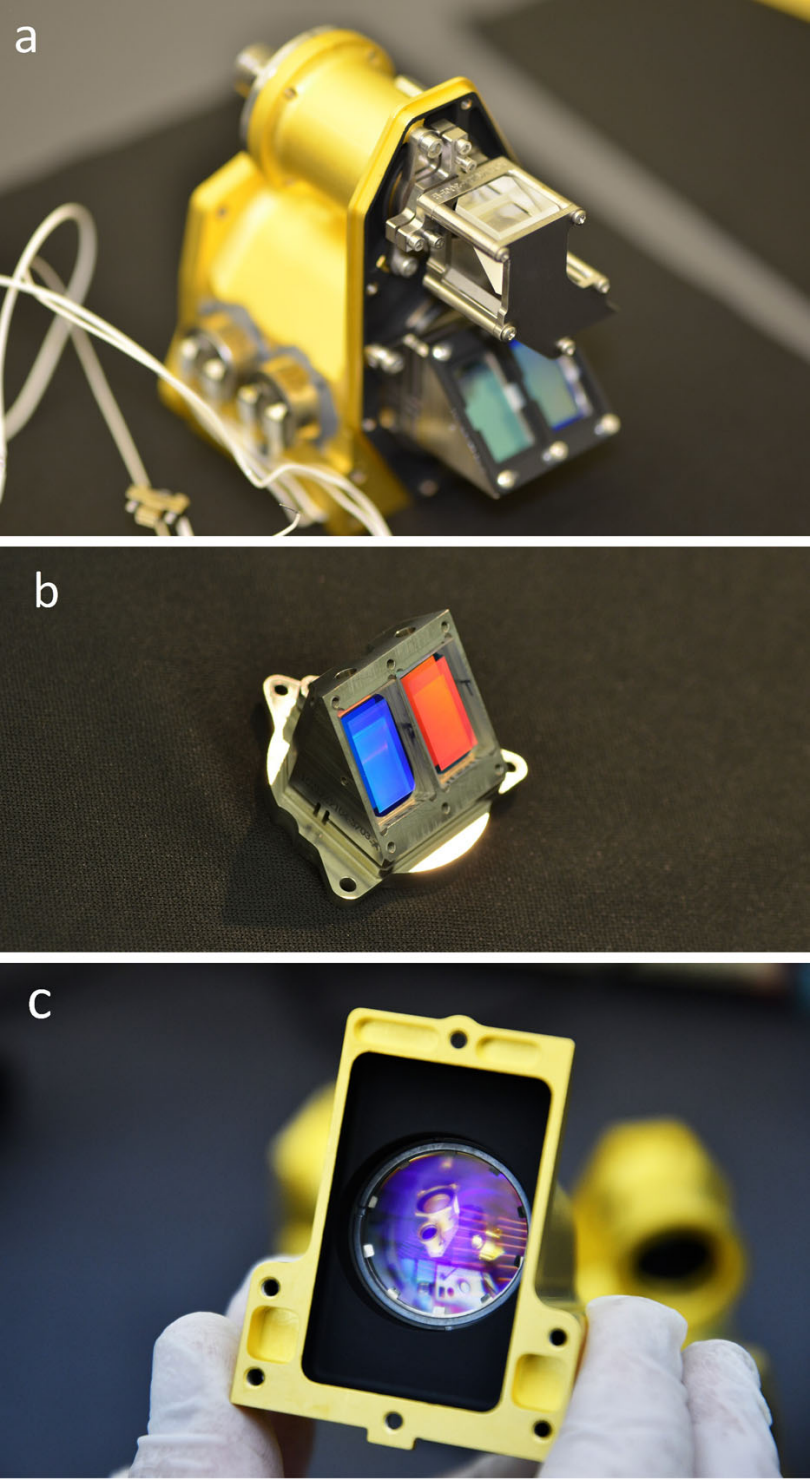
Subsections of the transmission spectrometer. The spectrometer section (**a**) is shown with the fiber connector facing to the rear. The dichroic beam splitter is in focus. The grating assembly (**b**) is shown with the input side visible, and relay lens is seen in its housing (**c**), looking from the CCD side. Other components on the table are visible through the relay lens

The dichroic beam splitter (Figs. 14, 15a) receives light from the collimating lens and reflects it down toward the gratings. Below the reflecting mirror, a custom dichroic mirror from OptoSigma, positioned at a $45^{\circ}$ angle, passes $>93\%$ of light from 720–850 nm directly down to the red-band grating while reflecting to the side $>95\%$ of light from 530–700 nm. This light forms the green and orange bands. It is reflected by another $45^{\circ}$ mirror straight down toward the compound green-orange grating.

**Fig. 16:**
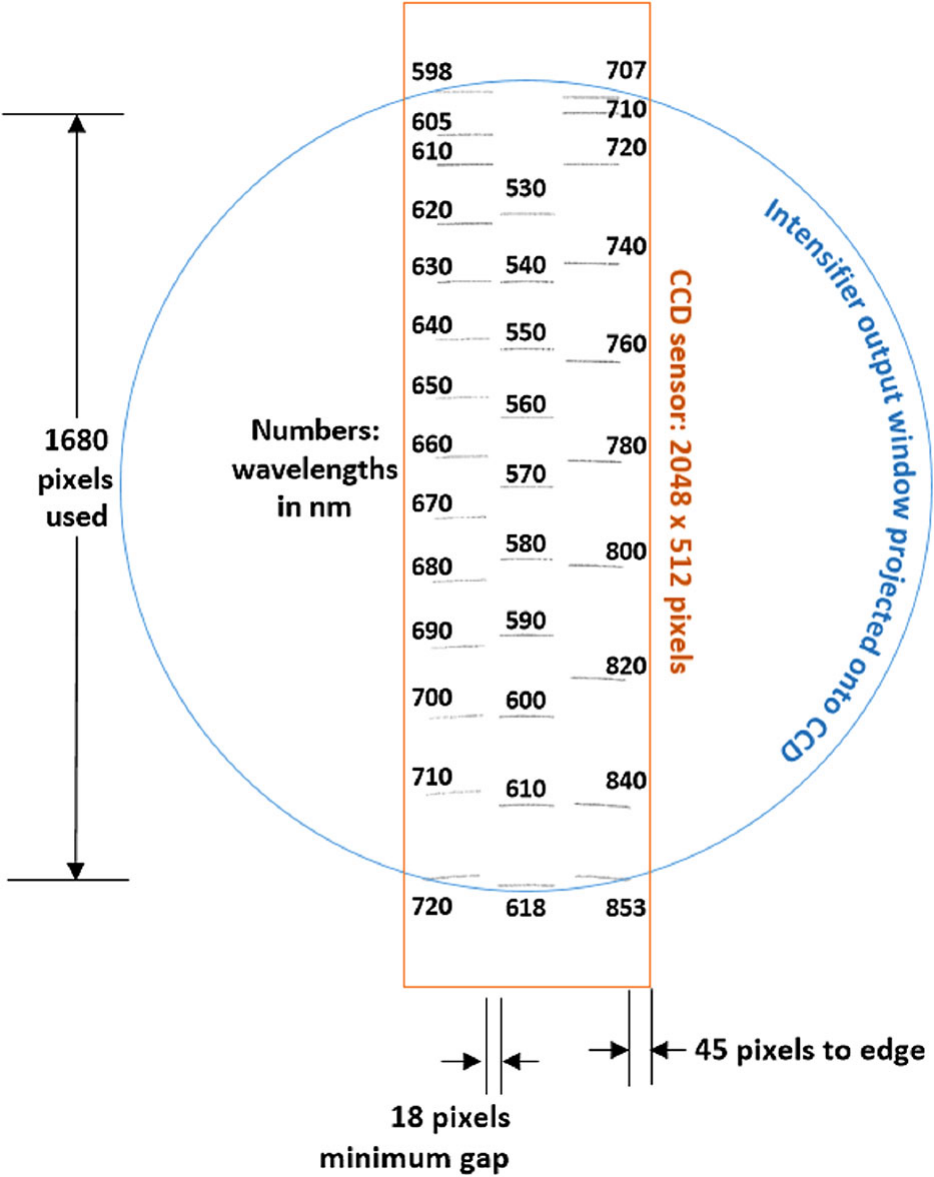
Map of spectrometer slit images at various wavelengths (in nm) with the field of view of the intensifier (circle) drawn for scale. The green band (middle, 530–618 nm) starts part-way in from the CCD’s edge in order to optimize the resolution. The orange band (598–720 nm) is projected to the left, and the red band (707–853 nm) to the right. The CCD is read-out from the right side in this orientation. Compare with the bottom panel of Fig. 12

The two parallel beams are directed onto three transmissive diffraction gratings (Figs. 14, 15b); the long wavelengths $>715~\text{nm}$ go to one grating, and the shorter wavelengths of 530–715 nm go to a duplex compound grating. The diffractive element in the 715–855 nm beam is a single 1800 lpmm grating. The compound grating contains two diffractive elements: a 2480 lpmm grating optimized for the 530–620 nm range (nominal center: 570 nm), and a 2110 lpmm grating optimized for the 610–700 nm range (nominal center at 670 nm). The rulings in the two gratings are rotated 1.8 degrees from each other, so that the diffracted spectra are spatially separated on the intensified CCD (ICCD) input window. The single 1800 lpmm grating is rotated by an additional 1.8 degrees so that its diffracted output is spatially separated from the other two. Volume-phase (VPH) transmission gratings from Wasatch Photonics Inc. were previously flight qualified as part of the ExoMars/RLS instrument (Rull et al. 2017). The output of the three VPH diffraction gratings is a set of three spectral bands stacked in the spatial direction on the face of the ICCD (see Fig. 16). An achromatic lens assembly, similar to a scan lens in design, focuses each spectral component onto the photocathode of the ICCD sub assembly. The images of the slit on the ICCD face are magnified 1.22 times by the scan lens assembly.

The ICCD subassembly consists of three main elements: intensifier tube, CCD sensor, and relay lens (Fig. 15) to carry light from the intensifier to the CCD. The intensifier tube provides two essential functions for Raman and TRL spectroscopies: It amplifies the weak optical signal returned from the sample and allows very fast (requirement to $\leq100~\text{ns}$) gating. This gating is essential to isolate the brief Raman signal from the delayed and much longer-lived luminescence generated by the sample (Sect. 7.4) and to study time-resolved luminescence.

Primary ICCD design considerations are optical gain, resolution, spectral responsivity, gating ability, and flight qualification. Working closely with ITT Exelis in Roanoke VA (now part of Elbit Systems), we identified an intensifier which meets all requirements. The intensifier features a continuously variable gain over 45 dB photon/photon, resolution exceeding 48 lpmm, and a photocathode with quantum efficiency (QE) over 30% from 530 nm to $\sim900~\text{nm}$. Gain is adjusted by selecting the voltage across the tube’s microchannel plate, and signals are time-gated by gating the voltage.

A high-performance relay lens assembly couples light from the intensifier phosphor output to the CCD. A custom double-Gauss lens design is used to meet requirements for high coupling efficiency (f/2.7), $>30^{\circ}$ FOV and compact size. Lens design takes advantage of the fact that light exiting the intensifier is nearly monochromatic at 545 nm. The intensifier’s P43 phosphor does generate minor outputs at other wavelengths, but these are suppressed by coatings on the relay lens surfaces. The resolving power of this relay lens assembly surpasses that of the intensifier tube. The paraxial magnification of the relay lenses is −1.26. A 20 μm feature on the intensifier maps to 25.2 μm on the CCD, which corresponds to 1.87 pixels.

### Mechanical and Thermal Description

#### Mechanical

**Fig. 17:**
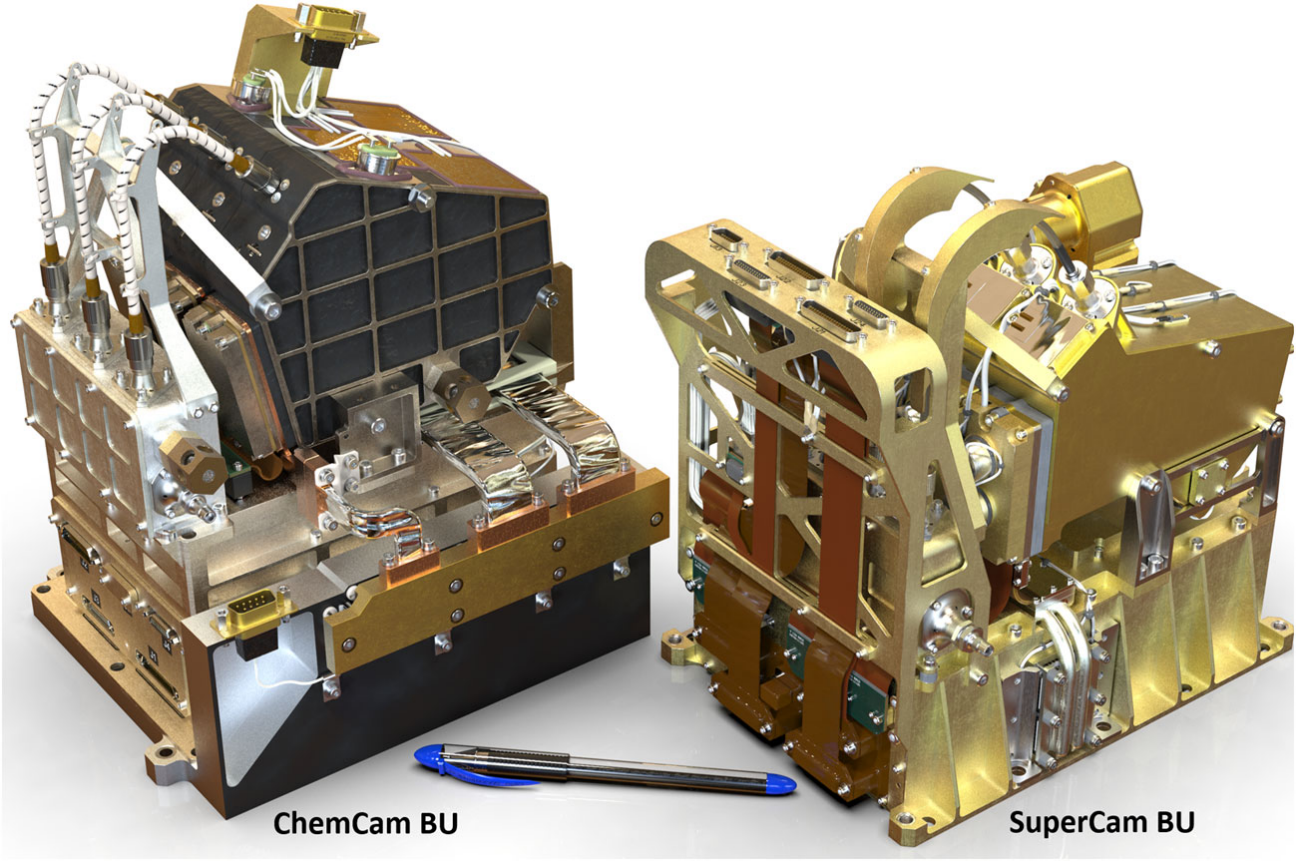
Side-by-side rendering of ChemCam and SuperCam Body Units. SuperCam’s BU is 345 g lighter and occupies a slightly smaller volume

The SuperCam BU mechanical design uses ChemCam as its starting point (Fig. 17), but incorporates many refinements and design changes driven by the specific SuperCam requirements. The BU consists of five major sub-assemblies (Fig. 4). These are the electronics box, demultiplexer, reflection spectrometers, transmission spectrometer, and connector interface bracket. The design is modular, allowing for each sub-assembly to be assembled and initially tested and aligned before final integration. This modularity was also intended to minimize the impact upon other modules in case disassembly was needed for trouble shooting.

**Fig. 18:**
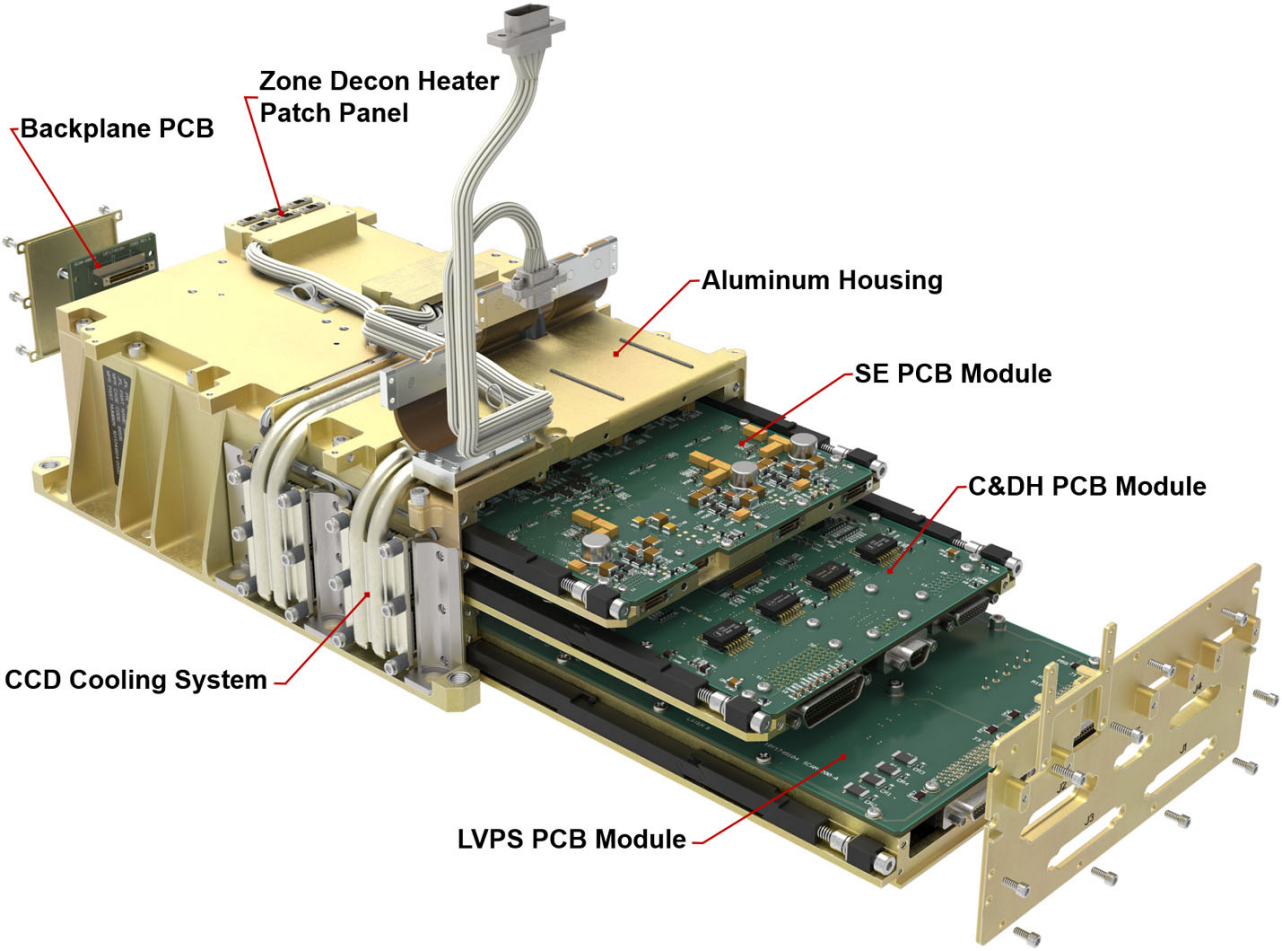
An exploded view showing the major components of the electronics box (EBOX). Its backplane/daughter-card architecture allows easier removal and installation of circuit boards during assembly and testing. The EBOX acts as the support structure for the optical sub-assemblies

The electronics box (EBOX) (Fig. 18) is an aluminum structure that contains the spectrometer electronics (SE) printed circuit board (PCB), command and data handling (C&DH) PCB, and the low-voltage power supply (LVPS). It also functions as a stable platform for the other sub-assemblies mounted on its top panel. Additionally, the EBOX serves as the thermal interface to the RAMP; the interface is described in Sect. 3.2.2. The EBOX design uses a backplane/daughter-card architecture. Each daughter card can be easily and independently installed or removed without significant impact to the overall assembly, and with no impact to the opto-mechanical assemblies. This is an improvement over the ChemCam design. All electrical connections between boards are through the backplane using nano-D connectors. Each daughter board is mounted to an aluminum frame, which acts as a thermal sink and provides stiffness to the board in vibration. Each module slides into the housing and is clamped in place with wedgelocks. The wedgelock joints provide a reasonable thermal interface between the housing and the module frame. The LVPS frame also makes up the base panel of the EBOX. On the top of the EBOX is a patch panel for distributing power to the decontamination heater systems. This panel uses nano-D connectors to minimize mass and volume, and allowed easy electrical connection of heaters during assembly of the major optical subsystems onto the EBOX.

The demultiplexer uses the same overall architecture as ChemCam’s but was optimized for SuperCam. This included reducing the overall length by $\sim25~\text{mm}$ to allow more room for the transmission spectrometer, and introduction of an improved fiber cable support bracket to better support the fiber cables in vibration and to protect the fiber optic cables during handling. The modifications also included accommodations for the refined optical elements. Figure 7 shows the opto-mechanical design of the demultiplexer. The lenses used to focus the light onto the fiber tips are mounted in titanium barrels with anti-backlash springs. The focal distance was adjusted during assembly by turning the barrels which were easily accessible with the demultiplexer cover removed. Two-dimensional alignment of the fibers was accomplished by translation of the fiber tip and connector across the top surface of the demultiplexer body. Once aligned and focused, the barrels and fibers were locked in place to prevent movement in vibration. The demultiplexer is also equipped with decontamination heaters, thermostats, and a light-tight venting baffle.

The reflection spectrometers were based on the same basic optical design as ChemCam but the mechanical designs were significantly refined (Fig. 11). The SuperCam reflection spectrometers are composed of two mechanically identical spectrometer assemblies bolted together. For ChemCam, the three reflection spectrometer housings were manufactured from beryllium. Due to the health-related complications of using beryllium and to improve thermal stability, it was decided to use titanium for SuperCam’s spectrometers. Titanium has a significantly lower CTE than beryllium and 97% lower thermal conductivity which helps thermally isolate the optics. Titanium is 2.4 times denser than beryllium, and therefore the overall design required significant optimization to keep the mass as low as possible. In the end, each SuperCam spectrometer had almost the same mass as those on ChemCam. The optical mounts were also refined to reduce mass, improve bonding of optical components, and make optical adjustment easier than on ChemCam. Each optic is mounted on compact kinematic mounts that can be adjusted from the exterior of the housing. Once adjusted and staked, a cover was placed over the mounting region. This cover provides a light tight seal as well as a mounting location for decontamination heaters. Each spectrometer housing vents through a light-tight baffle equipped with an 18 micron filter mesh to protect from particulate contamination.

The transmission spectrometer (Fig. 14) is an entirely new design and accounted for a large fraction of the developmental work to create a compact robust package that could fit within the allocated SuperCam volume and mass limitations. The lenses are mounted in Ti barrels with finger springs and retaining rings. Titanium was chosen to better match the CTE of the lenses to minimize thermal stresses on the lenses and allow for tighter tolerances. A number of the optical elements required the ability to adjust after assembly. These adjustments then required locking to prevent motion in vibration and thermal cycling. Some of the barrels could be adjusted for focus from the exterior of the spectrometer and then locked in place. The fold mirrors and diffraction gratings were mounted in Ti flexures that allowed tip/tilt adjustment. Below the transmission spectrometer is the high-voltage power supply (HVPS) (Fig. 14) that powers the intensifier. The HVPS is attached to the intensifier assembly only by the electrical leads; it is isolated thermally and structurally to eliminate any thermal leaks or induced stresses into the optical assembly. Packaging the HVPS in the small space below the transmission spectrometer required special care.

**Fig. 19:**
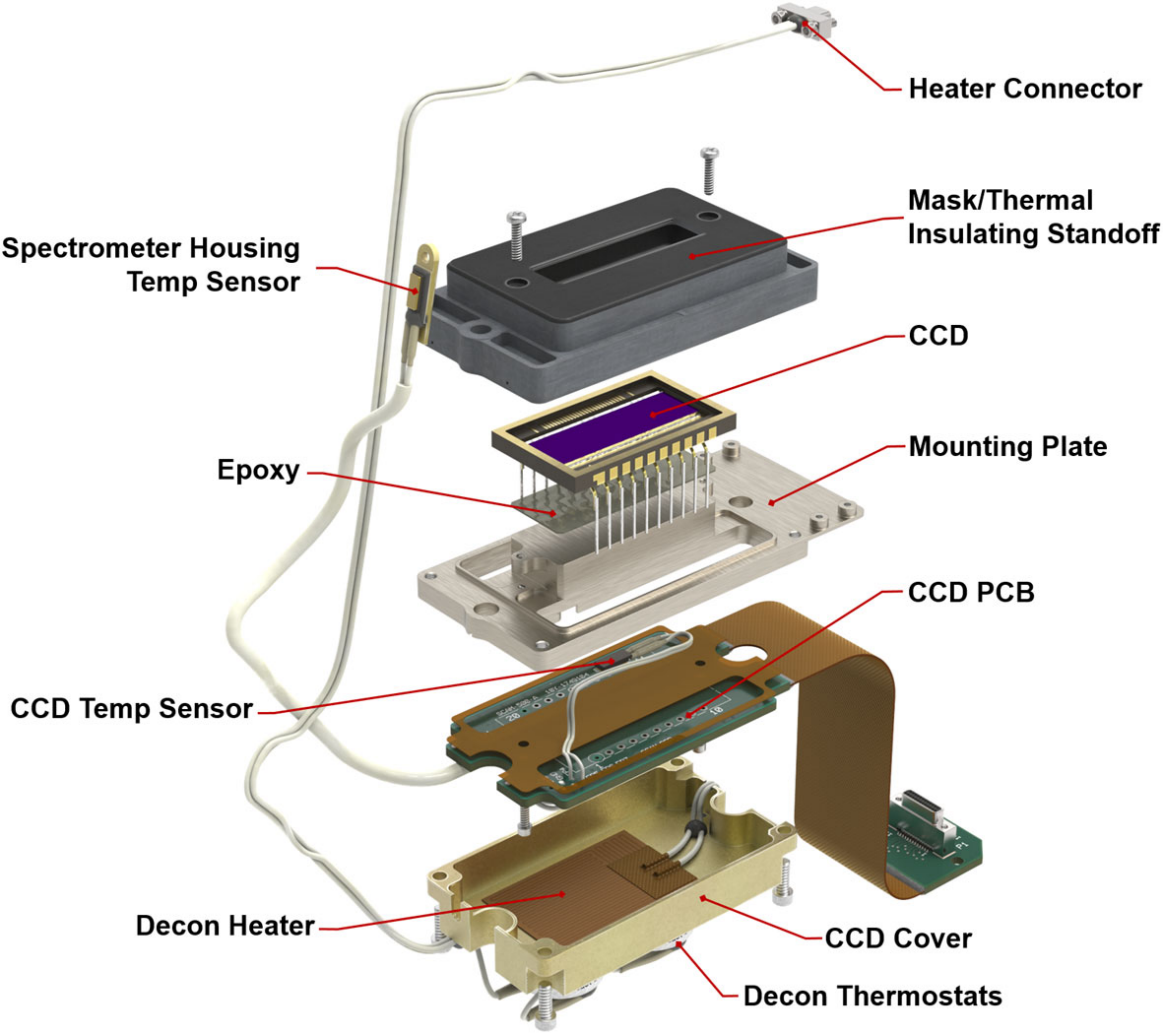
Exploded view showing the assembly of the CCD module. The basic design is common to all three spectrometers. The VIO CCD does not have a temperature sensor for its spectrometer housing

All three spectrometers have mechanically identical CCD modules mounted to the rear of the spectrometer housings; they are thermally isolated from the housings (Fig. 19). The modules contain the CCDs and CCD PCBs as well as decontamination heaters and thermostats which interface with the cooling system on the EBOX. The CCD boards connect to the SE board via flex cables routed to maintain more than the minimum bend radius for the cables. Additionally, two of the CCD modules have thermostats that measure the external housing temperatures for the spectrometer units and report this information in the state of health data.

Due to the very confined space SuperCam occupies in the rover, a connector interface bracket was added to provide easier access to the electrical connectors during integration with the rover (Fig. 3). Flex circuits route the connections from the EBOX PCBs to the top of the connector bracket.

Despite significant increases in capability and performance, the final flight SuperCam Body Unit ended up 345 g lighter than ChemCam’s, occupying a slightly smaller volume.

#### Thermal

The SuperCam BU thermal design consists of two active systems for CCD cooling and for decontamination, and passive design elements for cooling of electronics and thermal stability for optical systems. In all cases, the major thermal interface for the instrument is the RAMP, which is a fluid-loop-controlled mounting surface for avionics and other instrumentation. The RAMP provides an interface temperature within −40 to $+50~^{\circ}\text{C}$. Based on experience with the MSL rover, which has the same overall thermal design, the RAMP interface to the BU is expected to be between 0 and $+35~^{\circ}\text{C}$ on Mars. In order to maximize heat transfer between the instrument and the RAMP, SuperCam’s EBOX is mounted with a thermally conductive room-temperature vulcanizing (RTV) silicone. In addition to mechanical coupling to the RAMP, the instrument is radiatively coupled to surrounding electronics modules, rover belly pan (the covering on the underside of the rover), and rover sidewall. The sidewall and belly pan were modeled to reach temperatures as cold as $-130~^{\circ}\text{C}$, while the surrounding avionics temperatures can vary widely based on operational usage. Finally, the Martian atmosphere, while thin, does facilitate convective heat transfer that must be considered, especially in the CCD cooling design.

There were three thermal requirements that drove the design of the thermal management systems on SuperCam. First was maintaining the electronics at an acceptable temperature to maintain reliability and avoid thermal induced failures. This was accomplished by mounting the three EBOX PCBs on aluminum frames that would efficiently transfer their heat to the housing via wedgelock joints, as described above. The LVPS, being the highest heat dissipater of the boards (7.32 W maximum), is located lowest in the housing and its frame doubles as the base panel of the EBOX. This allowed the LVPS to be wet-mounted (with RTV) to the RAMP along with the EBOX frame, providing a direct heat sink. The power converter on the LVPS is mounted such that it can dissipate heat directly to the frame. The other two EBOX PCBs, the SE and C&DH, are mounted to the wedgelock-equipped aluminum frames mentioned above, and transfer their heat through the EBOX walls into the RAMP. The total heat dissipation from SuperCam’s BU when taking data is 43 W with most of the heat being generated by the TECs, described below. The design was verified with finite-element modeling and thermal/vacuum testing. The other passive thermal control feature is the gold plating on the titanium spectrometer housings. This low-emissivity plating effectively decouples the optical assemblies from the temperature extremes of the surrounding environment, improving thermal stability of the instrument.

**Fig. 20:**
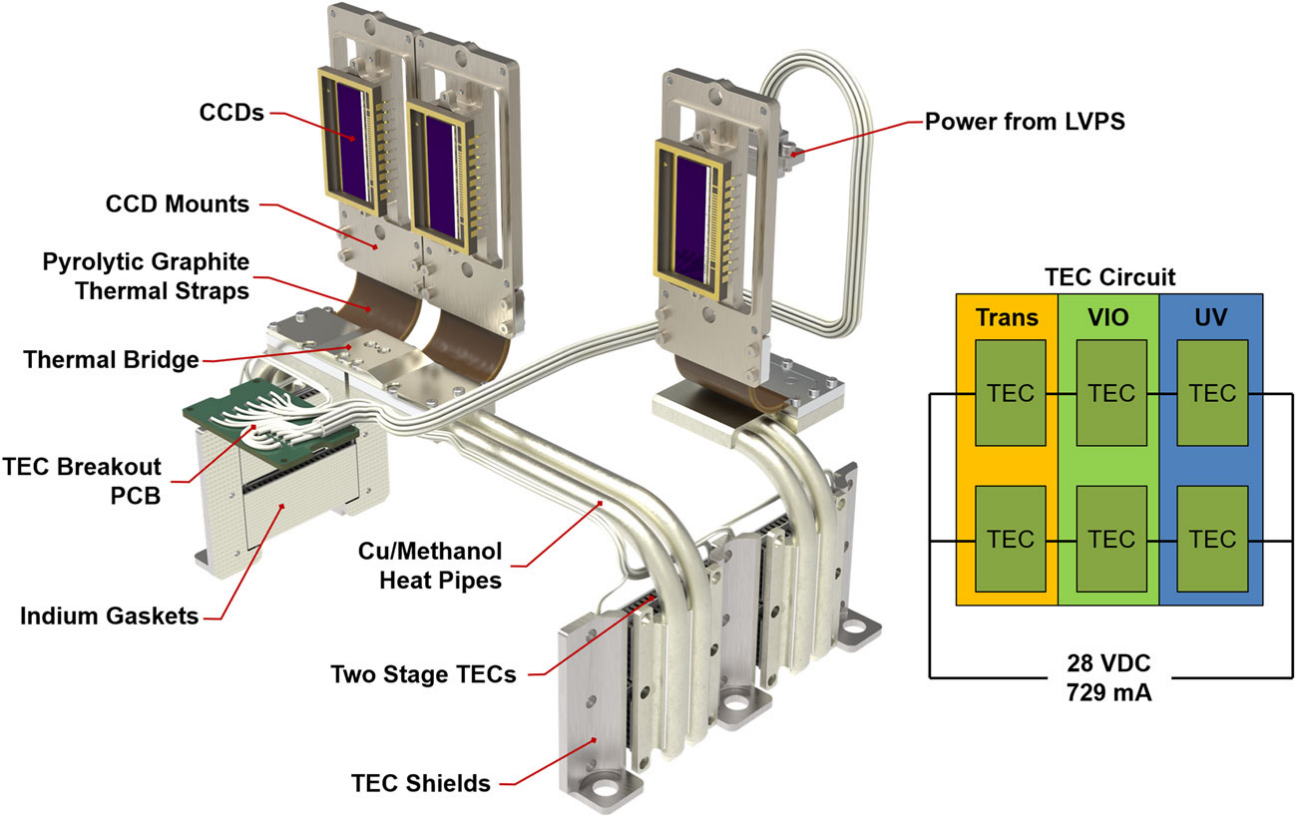
The system for cooling the CCDs shown in isolation along with the wiring schematic. The TEC shields provide both protection to the TECs during rover integration and improve the thermal path to the mounting fasteners

The second requirement was the need to cool the CCDs during operation. CCDs generate thermal noise (dark current), which can be mitigated by cooling the detectors. While colder is better, power limitations required a reasonable choice for target operating temperatures; previous experience on ChemCam (which uses the same CCDs) has shown that $0~^{\circ}\text{C}$ is a sufficient target temperature for achieving low noise with reasonable cooling power. The cooling is achieved via TECs, which are solid-state devices that use the Peltier effect to pump heat from a cold plate on one side to a hot plate on the other. Only about 15% of the power provided is converted to active cooling, but the simplicity and lack of moving parts create a very robust cooling system. SuperCam uses the same TECs as ChemCam (Wiens et al. 2012), operated in two parallel strings of three TECs wired in series and cross strapped (see Fig. 20). This configuration is powered directly from the rover through an instrument-command-controlled relay located on the LVPS board. The rover nominally supplies 28 V, or 9.33 V per TEC, with a current draw of 729 mA, for a power consumption of 6.8 W. The operating voltage of the TECs was limited by the amount of heat the RAMP could dissipate, thus limiting available cooling power. The TECs are arranged such that each CCD is cooled by a pair for an overall 13.6 W of TEC power, but due to the inherent inefficiency of TECs only about 2 W of this is actual cooling power, with the balance of the power consumption resulting in waste heat.

This waste heat on the hot side of the TECs drove the majority of the cooling design; in order to efficiently dissipate this heat without impacting the rest of the instrument, the TECs needed to be mounted as close to the RAMP as possible (unfortunately they could not be mounted directly on the RAMP due to space limitations; Fig. 3). They are mounted on the sides of the E-Box near the mounting flange (Fig. 18), while the CCDs are mounted up on the spectrometer assemblies, creating the need for an efficient thermal path between the TEC cold plates and the detectors. To accomplish this, a pair of 5 mm copper-methanol heat pipes are bonded to each TEC pair, and run along the EBOX to just under the CCD assemblies of their respective spectrometers (Fig. 20). The heat pipes use methanol as a working fluid. To avoid creating a rigid connection between the EBOX and the CCD assemblies, which would induce structural loads and drive tight assembly tolerances, the heat pipes are thermally connected to the CCD mount assemblies via annealed pyrolytic graphite (APG) thermal straps. These straps are approximately twice as conductive as copper at 740 W/mK, while using only three layers of polyimide encapsulated graphite as the conductive path. This creates a relatively flexible, high conductivity coupling between heat pipes and the CCD mounts. The CCD mounts themselves (Fig. 19) are made of copper, and are bonded to the CCDs with the same thermal epoxy used to mount the heat pipes to the cold plates of the TECs. This copper mounting plate provides both a high-conductivity path to the cooling system, and a significant thermal mass to reduce (damp) thermal fluctuations. The CCDs have a manufacturer-recommended maximum temperature change rate of $5~^{\circ}\text{C/min}$, so the thermal mass of the mounting plates is important because the TECs are not actively controlled. They are at full power when switched on and provide a rapid rate of initial cooling. All other thermal interfaces in the cooling chain were either bonded with application-specific material or used indium gaskets to minimize losses to contact resistance. Note in Fig. 20 that the cooling systems for the UV and VIO CCDs are coupled. This allows the two systems, which have differing heat-pipe lengths, to balance the cooling provided and keep CCD temperatures similar on both channels. The 2 W of cooling power generated by each TEC pair provides a stable $\sim20~^{\circ}\text{C}$ of cooling below the RAMP temperature; only a little under half of this cooling power is needed to overcome the active load of the detectors ($\sim875~\text{mW}$). The remainder of the cooling power must overcome parasitic losses through the insulating Noryl mounts to the EBOX and spectrometer housings, and convective losses to the Martian atmosphere.

The SuperCam TECs are controlled by the instrument, in contrast to the ChemCam TECs which are controlled at the rover level. This is a disadvantage, as pre-cooling of ChemCam’s CCDs can be done before powering the instrument on, while for SuperCam the instrument must be turned on to start cooling the CCDs. Thus the TECs must fight the heat generated by the instrument. This disadvantage for SuperCam resulted from a dearth of power switches available on the rover. Neither ChemCam nor SuperCam meet the thermal requirement of $0~^{\circ}\text{C}$ for the CCDs throughout the day on Mars (based on experience with ChemCam and modeling with SuperCam), as the RAMP warms over the course of the day. Rather, observations by both instruments are preferred to be made during the morning, when the requirement is met; afternoon observations have noisier backgrounds.

**Fig. 21:**
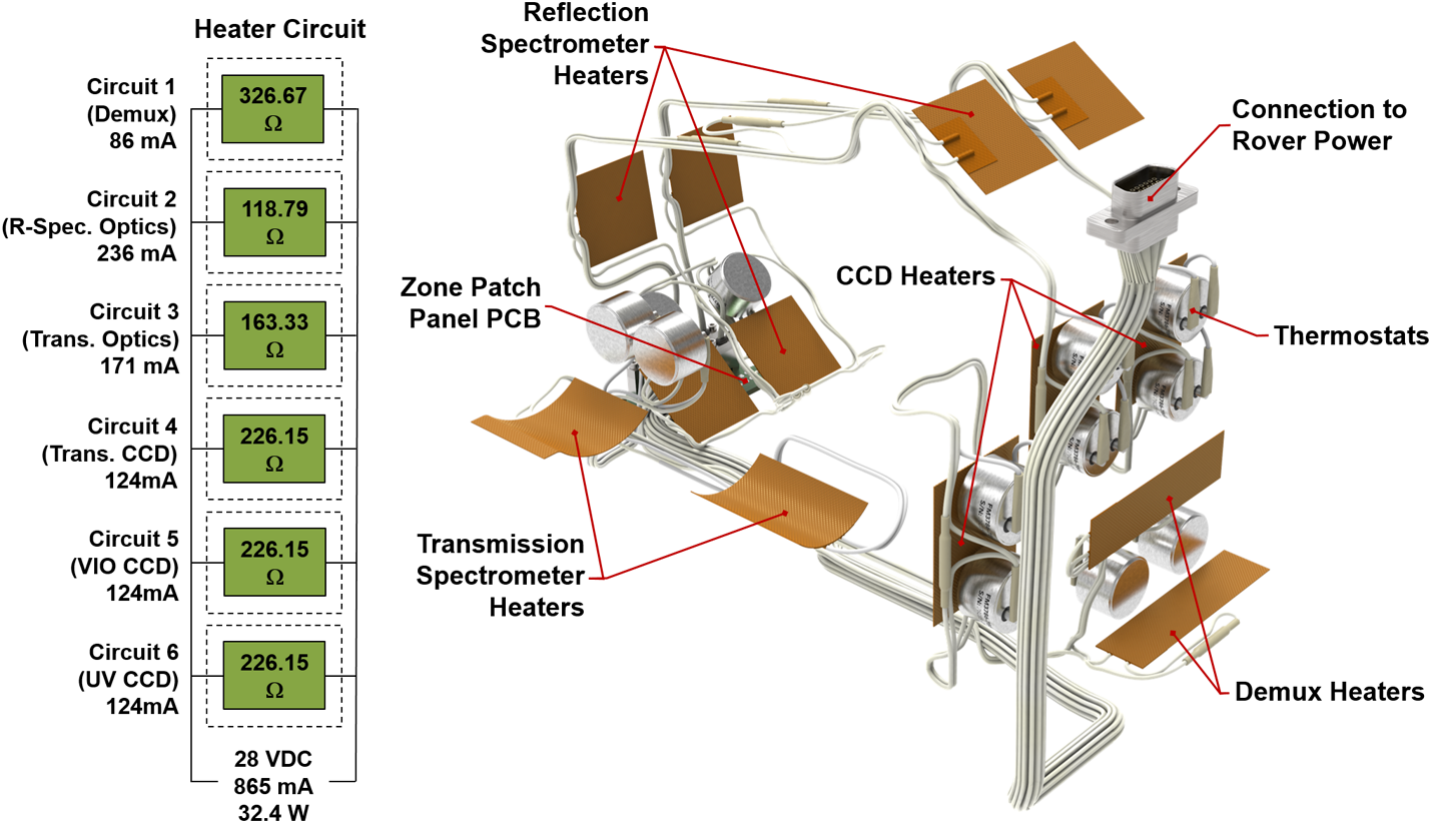
Decontamination heater system shown in isolation along with the wiring schematic. The system is composed of six independent zones, each controlled by a pair of redundant thermostats and operating on rover power

A third thermal requirement was the need to maintain clean optics during the cruise phase to Mars. Since the SuperCam BU is inside the rover body, it shares the space with electronics and cabling that may outgas material that may condense onto the optical surfaces, resulting in degradation in the UV range. This condensation could happen when the operating electronics are significantly warmer than the SuperCam optics. The solution was to strategically place decontamination heaters to raise the temperature of the optics above the maximum expected temperature of the surrounding hardware. This temperature was determined by JPL to be $+40~^{\circ}\text{C}$. In a departure from the ChemCam design (Wiens et al. 2012), the heaters are controlled by redundant thermostats rather than sensors and rover flight system software. The heaters were sized and thermostats set points selected so the temperatures cycle between $+43$ and $+50~^{\circ}\text{C}$ using rover bus power and a rover power switch (necessary because SuperCam is turned off during cruise). There are six independently controlled decontamination heat systems: one for each CCD assembly, one for the demultiplexer, and one for each spectrometer assembly. The overall system and the electrical schematic for the decontamination heating system is shown in Fig. 21. The maximum duty cycle of any heater at 28 V (the nominal rover bus voltage) is 72% at the coldest part of cruise. The maximum power consumption of the systems if all heaters are running simultaneously is 24.2 W.

This design was challenging because the CCDs are attached to a thermal system designed to efficiently remove CCD heat. Fortunately, the TECs have a fairly high thermal resistance when not powered so it was possible to size the heaters so the CCDs reach the required temperature. To maintain the elevated temperature on the demultiplexer it was necessary to add thermal standoffs under its feet to reduce thermal losses to the EBOX. There are a total of 12 thermostats, and 13 heaters in the system. All heaters except the CCD heaters (which are internal to the CCD cover and thus do not radiatively couple directly to the environment) were coated with low emissivity tape to better match the housing gold plating and reduce radiative exchange with the surrounding environment.

**Fig. 22:**
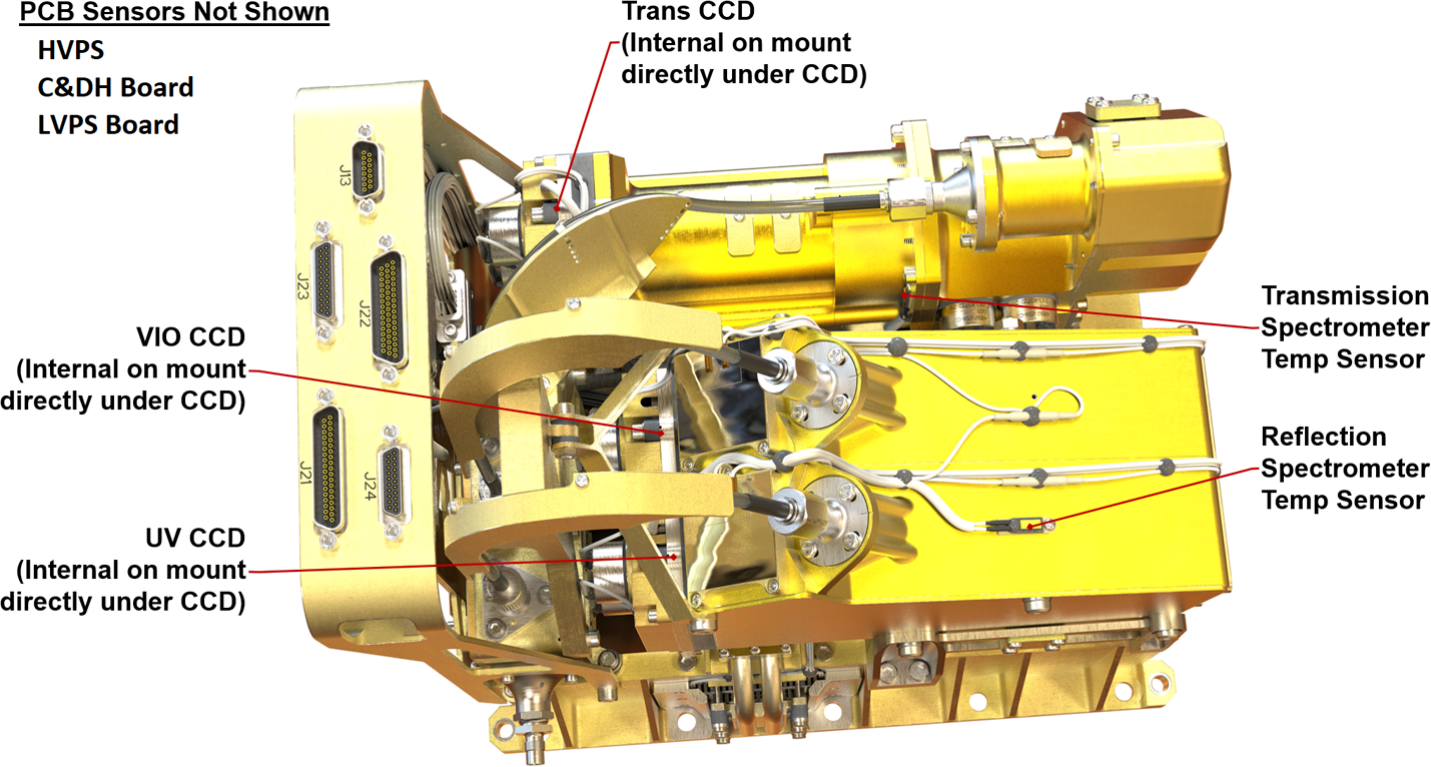
Locations of all the SOH temperature sensors on the SuperCam BU. Locations were chosen to provide the best temperatures for calibration. Trans = transmission spectrometer

Figure 22 shows the locations of the state-of-health (SOH) temperature sensors. These sensors are located in and on the instrument to maximize useful data for calibration. Their values are recorded by SuperCam and are part of the SOH data package. Most critical are the sensors at the CCDs. These are mounted directly behind where each CCD is bonded (Fig. 19) to give accurate CCD temperatures. This is an improvement over ChemCam which had only one sensor on the exterior cover of the UV CCD, giving only a rough indication of the CCD temperatures. ChemCam also had sensors on the spectrometer and demultiplexer housings. These were monitored by the rover and had to be extracted from the rover SOH data, while on SuperCam these temperatures are read by the instrument and are found in the instrument data files. SuperCam has sensors on the transmission and reflection spectrometer housings to provide their temperatures; it was deemed unnecessary to monitor the demultiplexer. There are also sensors on the LVPS and C&DH PCBs and the HVPS.

### Electronics

The electronics for the SuperCam BU consist of a LVPS board, C&DH board, and a spectrometer electronics (SE) board, all of which are mounted via the wedge-lock system inside the EBOX enclosure and electrically connected via a small backplane (Fig. 18). Additionally, CCD front-end electronics and CCDs, a HVPS for the intensifier, control circuits for survival heaters and TECs, and thermal sensors are electrically controlled and/or monitored by the modules in the EBOX. We will describe the low voltage electronics first, then the high-voltage.

#### Low-Voltage Power, Control, and Data

**Fig. 23:**
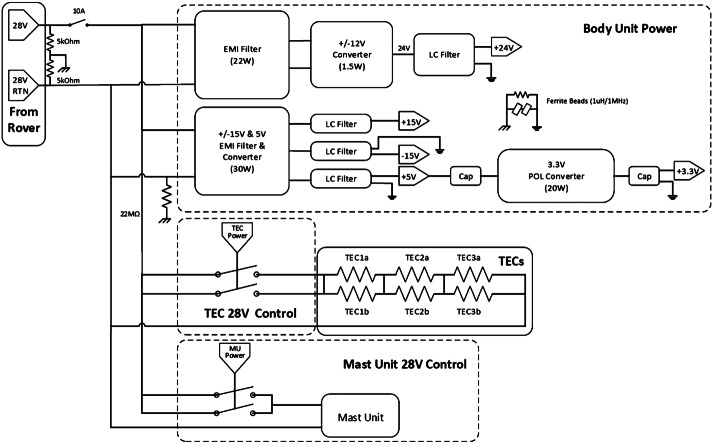
Schematic diagram of the BU low-voltage power supply. RTN = return; EMI = electromagnetic interference; Cap = capacitor; LC = inductance-capacitance

A schematic diagram of the BU power supply board is shown in Fig. 23. The BU receives unregulated power from the rover at a nominal value of 28 V. Power is passed directly to separate redundant switches for the MU and the TECs. The power is filtered before passing to all other circuits. For purposes internal to the BU, the following voltages are supplied: $+3.3$, $+5$, $\pm15$, and $+24~\text{V}$. The filtering requirement is for voltage ripple to be $<20~\text{mV}$ peak-to-peak, with voltage regulation within 5% or less, except for the 3.3 V, which has a 9% requirement. Requirements were also levied on maximum transient voltages on each circuit. When power is applied to the instrument, the LVPS secondary voltages become active, the C&DH begins the boot process, and the SE initializes to a default, idle state.

**Fig. 24:**
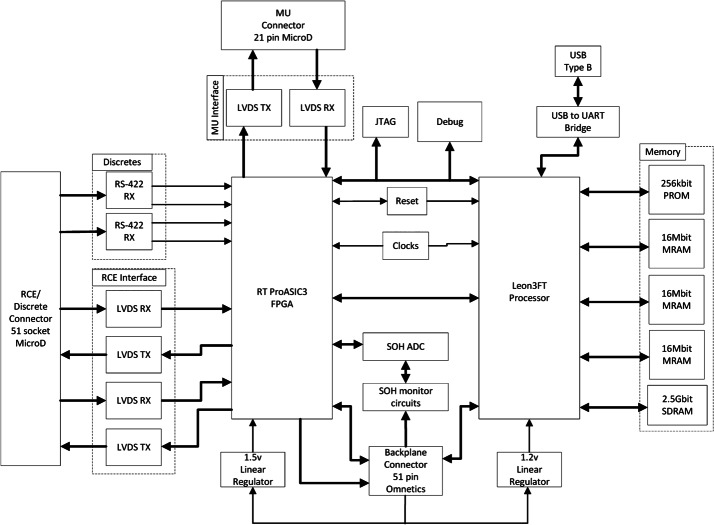
Schematic diagram for the BU C&DH board. See main text for definitions of abbreviations

The C&DH module (Fig. 24) is the primary SuperCam processor module, featuring a Leon3FT processor operating at 50 MHz, a Microsemi field-programmable gate array (FPGA) for custom logic, and an array of memory. Start-up read-only memory (SUROM) functionality is provided by a Cobham/Aeroflex programmable read-only memory (PROM) module. If program memory is corrupt, the SUROM is able to interface with the rover compute element (RCE) to accept a limited set of commands that allow the corrupt memory to be rewritten, if needed on Mars. Non-volatile program and parameter storage are maintained in a set of three Cobham/Aeroflex 2 Mbyte magnetoresistive random-access memory (MRAM) components. Finally, a Cobham/Aeroflex 2.5 Gbit synchronous dynamic random-access memory (SDRAM) module is used for volatile program and data buffer storage.

The C&DH is the primary digital interface between SuperCam and the RCE. The C&DH accepts commands and provides telemetry over a Mars 2020-standard low-voltage differential signal (LVDS) high-speed serial (HSS) interface operating at 1.94 million bits per second (Mbps) for commands (from RCE to SuperCam) and 7.5 Mbps for telemetry (from SuperCam to RCE). Reset and boot bank selection is provided via a set of RS-422 initialization discrete signals.

The C&DH also acts as the digital interface to the MU over a 6-meter flex cable between the BU and the MU. An LVDS HSS interface is used to transmit RMI and Microphone data products from the MU to the BU at up to 10 Mbps. In the C&DH FPGA, this HSS interface is mapped to one of the Leon3FT’s four SpaceWire interfaces at 30 MHz. An LVDS universal asynchronous receiver-transmitter (UART) interface at up to 9600 baud is the primary command and telemetry interface between the two units, and a set of LVDS discrete signals are used as a laser trigger, an intensifier trigger, and a reset for the MU.

A set of three SpaceWire links operating at 30 MHz interface between the SE module’s three CCD interfaces and the C&DH, which allows the use of direct memory access (DMA) to the C&DH SDRAM. A number of discrete signals to control the HVPS also originate on the C&DH and are passed through to the SE.

The C&DH controls two power switches on the LVPS board, used to control power to the MU and to the BU TECs, respectively. SOH circuitry is also provided on the C&DH to monitor an array of temperatures and voltages.

**Fig. 25:**
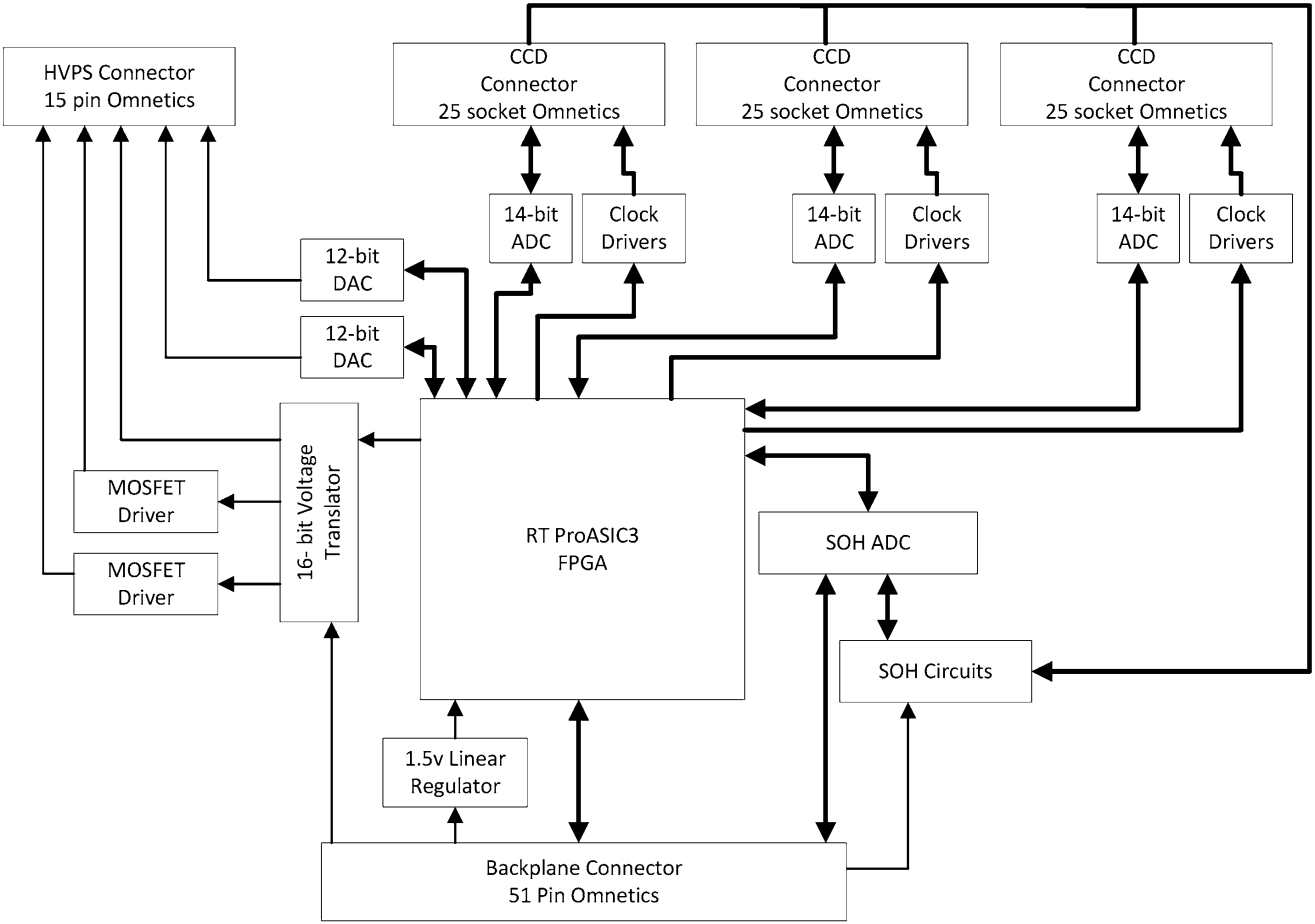
Schematic diagram for the BU spectrometer electronics board. ADC = analog-to-digital converter; DAC = digital-to-analog converter; Op Amp = operational amplifier; MOSFET = metal-oxide semiconductor, field-effect transistor. See main text for the meaning of other abbreviations

**Fig. 26:**
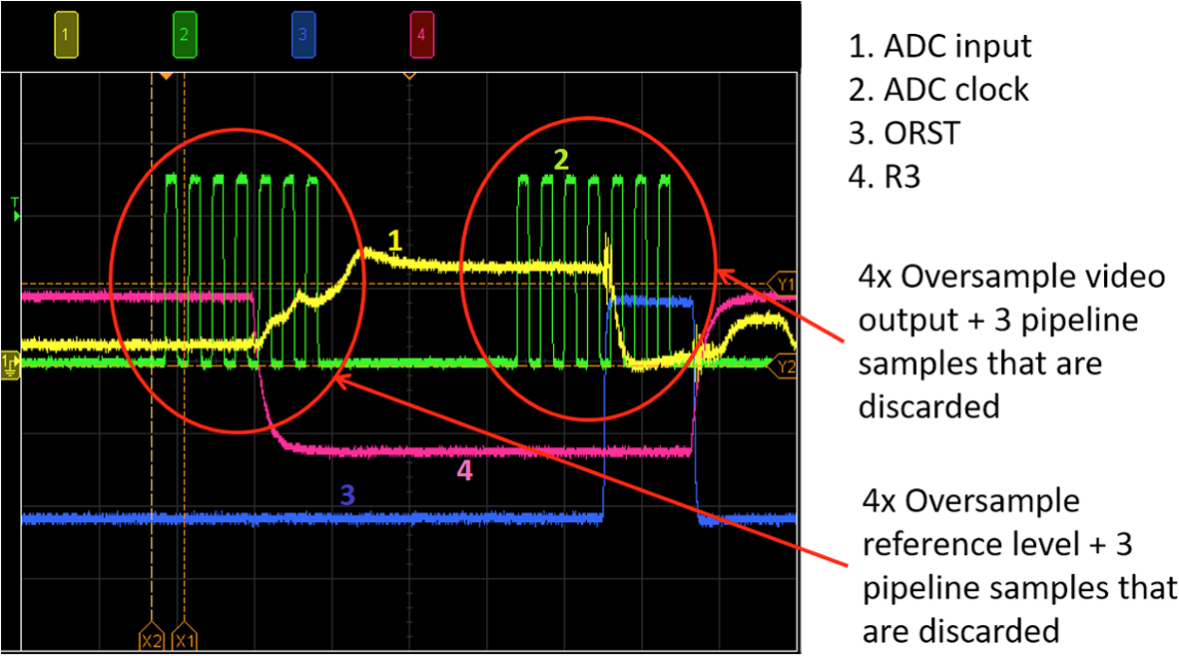
Oscilloscope traces illustrating the correlated quadruple sampling used to improve the signal-to-noise ratio. ORST is output reset pulse; R3 is readout register phase-3 clock pulse

The SE module’s primary function is to clock and read the e2v CCDs. A schematic diagram is shown in Fig. 25. A set of three ChemCam-heritage 14-bit Maxwell/DDC analog-to-digital converters (ADCs) are used. ChemCam-heritage CCD clock and voltage conditioning circuits are implemented on-board. A Microsemi FPGA is used to house the CCD clocking logic and SpaceWire firmware. The CCD pixels are digitized at 14 bits, but are over-sampled to 16 bits, taking the mean of four readings (Fig. 26). Additionally, a digital correlated double-sampling technique is employed to subtract the CCD reference voltage from the active pixel. These techniques together improved the detector read noise by a factor of over two versus ChemCam. One side effect is that a saturated pixel can read slightly below $2^{16}$ digital numbers (DNs) due to the averaging and subtraction that occurs simultaneously with the digitization. Another side effect is that the maximum transfer rate using this method is 400 kHz, which is about 20% slower than used by ChemCam. The result is somewhat higher dark noise due to longer transfer times (Sect. 4.2). Nominally, the CCDs are read in a row-summed one-dimensional (1D) mode. A two-dimensional (2D) diagnostic mode is also available, and can be used to image all rows of the CCDs (e.g., Fig. 12).

The SE board interfaces with the HVPS by providing the supply voltage from the LVPS, along with trigger signals from the C&DH and a gain voltage supplied by a 12-bit digital-to-analog converter (DAC). Board-level SOH circuitry is also provided on the SE for monitoring of SE, CCD, spectrometer, and HVPS temperatures, voltages, and currents.

**Fig. 27:**
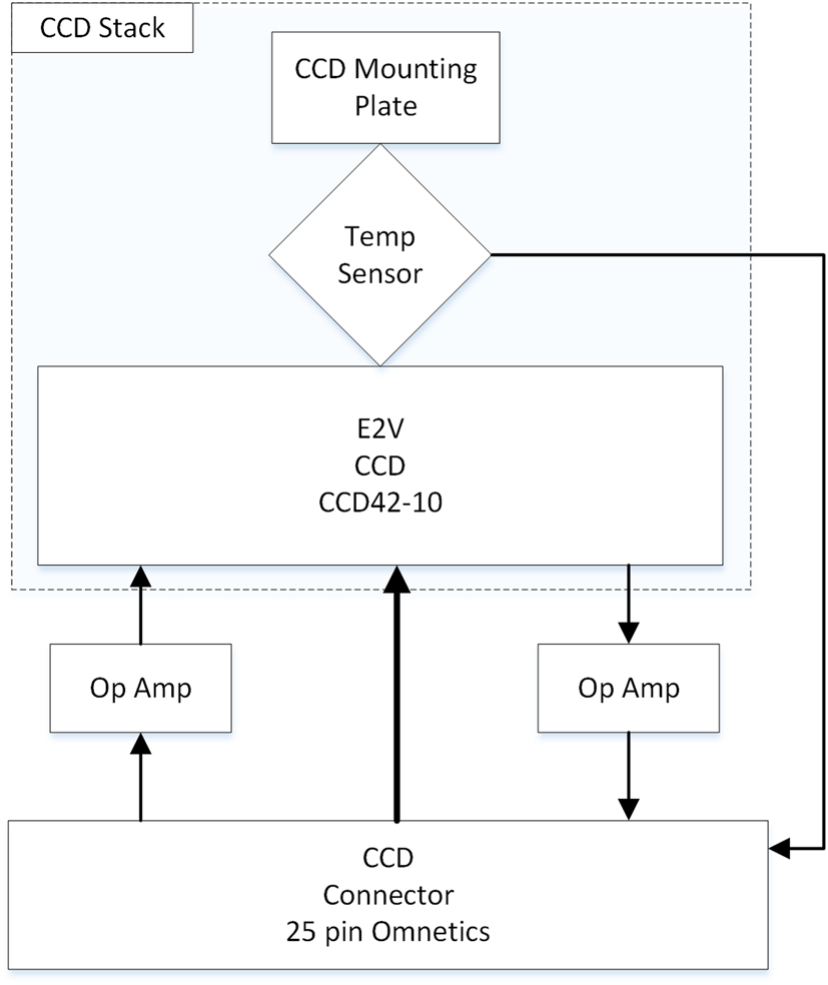
CCD board schematic diagram. Op Amp = operational amplifier; Temp = temperature. See main text for other abbreviations

The CCDs are each individually mounted to identical circuit boards (Fig. 27), which contain front-end signal-conditioning electronics. These modules are physically mounted in the three BU spectrometers, and are electrically connected to the SE via flex connectors.

A number of instrument temperature sensors are used to monitor the LVPS, C&DH, the CCD detectors, the HVPS, and the spectrometer housings. These sensors are only active when the instrument is powered on. They were calibrated during instrument thermal-vacuum tests.

#### High-Voltage Power Supply, Intensifier, and Timing with Respect to the Laser

**Fig. 28:**
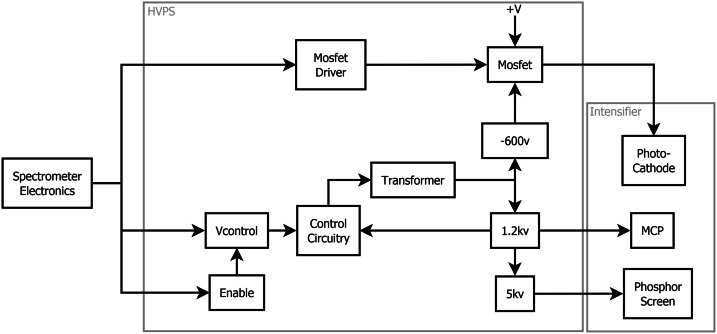
Simplified schematic diagram of the high-voltage power supply that operates the intensifier depicted in Fig. 14. MCP = micro-channel plate

A custom high voltage power supply (HVPS) powers the transmission spectrometer’s optical intensifier. A simplified schematic is shown in Fig. 28. The HVPS applies three bias voltages to the intensifier: $-600~\text{V}$ from the photocathode to the microchannel plate front, $+1200~\text{V}$ across the microchannel plate front to rear, and $+3800~\text{V}$ from the microchannel plate rear to the phosphor screen. Gain is adjustable via 0–5 V input from the SE board using a 12-bit DAC. This adjustment changes the bias voltage to the intensifier. Resolution is maintained across the range of gains used for this instrument.

The HVPS circuitry consists of a low-voltage side and a high-voltage side. The high-voltage side is potted to prevent arcing in the thin Mars atmosphere. Thermal-cycling life tests were performed on several HVPS units to ensure that they could survive a large number of diurnal cycles as will be experienced within the rover body.

**Fig. 29:**
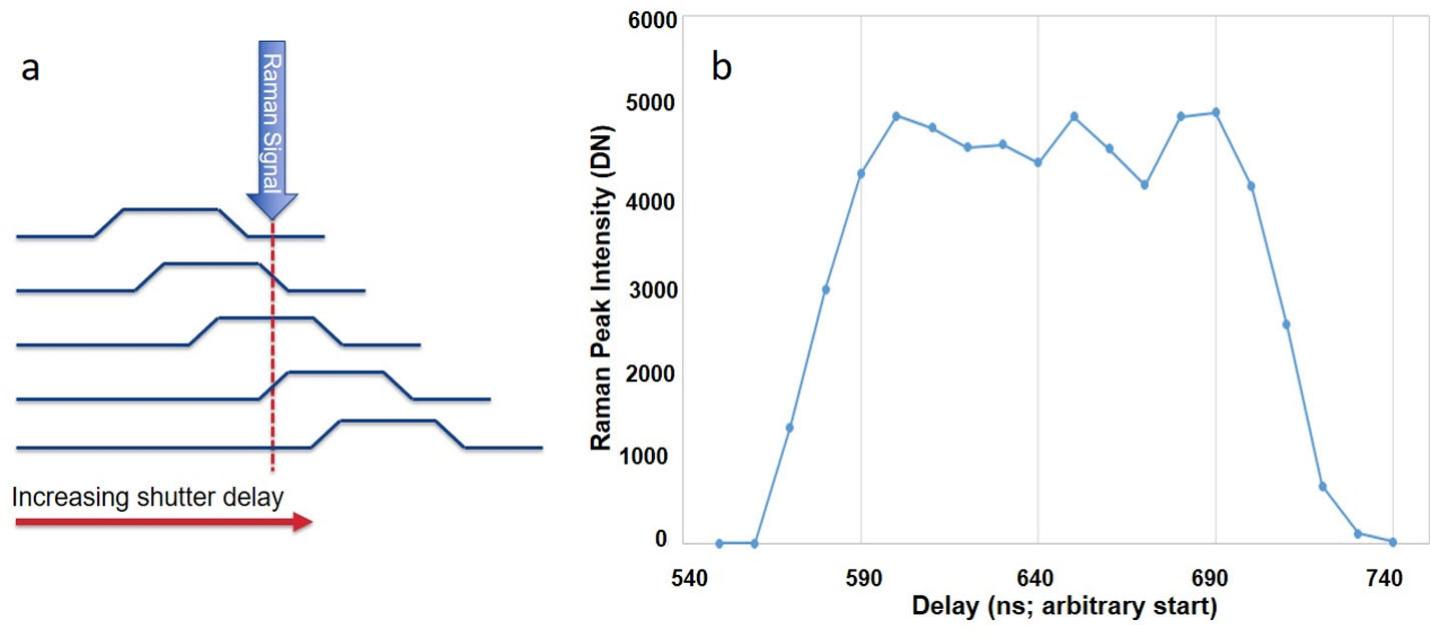
Description of testing the duration of the intensifier gate by generating a Raman signal on a target multiple times with different delays for each collect. The technique is illustrated schematically in (**a**). The result is shown in (**b**) for a 100 ns gate. Each point represents data collection at the delay given on the x-axis. The y-axis represents the Raman signal peak intensity for a given delay setting. The rising portion of the curve on the left of the plot (**b**) comes from signals received at the end of the integration period (**a**, top); the falling portion on the right in (**b**), at longer delays, represents signals received at the beginning of the integration period, as shown at the bottom of (**a**)

The C&DH board provides gate-open and -close pulses that are synchronized with the laser and with the CCD and its readout process. Diagnostic testing of the intensifier’s rise time and stability was performed by firing the laser at a Raman-bright target and making separate collects with different delay times. The laser pulse (and hence the Raman signal) duration is 4 ns (Maurice et al. 2020, this journal). The precision of the laser-to-intensifier timing is approximately $\pm14~\text{ns}$, as the two components are operated by different FPGAs in their separate parts of the rover (MU clock frequency is 20 MHz and the BU clock speed is 50 MHz; timing uses the half-frequency steps for double the accuracy). Figure 29 shows the results for a 100 ns intensifier gate, collected from an ensemble of readings at different gate times. The rise and fall times of the signal are 30–40 ns, a little longer than the sum of the timing uncertainty and the laser pulse duration, indicating that the intensifier goes from full off to full on in a few (10–20) nanoseconds. The minimum recommended gate time for this system is 100 ns. Shorter gates can be used, however, the transistor-transistor logic (TTL) pulses start to overlap, which can stress the system, and the gain curve becomes compressed. Given the timing uncertainties and variable observation distances, the peak of the gain curve may not coincide with the arrival of the light at the intensifier if shorter gates were to be used.

The top of the curve in Fig. 29b shows that stability is within $\pm10\%$. The slight dips at 640 and 670 ns are likely part of a fixed pattern. Tests with longer cables between the intensifier and the HVPS showed lower stability due to ringing caused by slight impedance mismatches in the cables. Given the expected reproducibility of the pattern, stability between observations made under the same conditions are generally better than the standard deviation across the plateau in the figure.

The zero point of the timing units on the x-axis in Fig. 29 is arbitrary. The laser actually fires at around $t = 660~\text{ns}$ in every case, and the delay is adjusted to move the 100 ns window around the time the light arrives at the detector. For a target $\sim2~\text{m}$ distant, it takes $\sim33~\text{ns}$ for the light to travel from the laser to the target, back to the telescope, and down the 6 m optical fiber to the BU spectrometer. A delay setting of 690 ns results in just catching the Raman signal as the gate opens completely. At the other end, a delay of 590 ns results in the intensifier observing the Raman signal just as the 100 ns gate is starting to close. The BU controls the firing of the laser, and the zero point of the intensifier delay ($t = 0$ extrapolation from Fig. 29) was designed to occur before the laser fires.

A number of details are covered during the sequence containing the laser pulse and the ICCD gate. First, the intensifier HVPS must be powered on, the CCDs must be powered on, and the laser capacitors must be charged. If the CCD is not yet integrating (i.e., for the first of several laser pulses to be collected on a single CCD exposure, or using single-shot exposures), then the pixels are continuously being dumped to the dump drain near the serial register. The CCD exposure is started and a “fire-the-laser” pulse is sent on a discrete line to the laser. The signal is received at the MU, which starts laser pumping and then triggers the Q-switch Pockels cell (Maurice et al. 2020). Simultaneously the MU sends a “laser sync” signal on a discrete line to the BU, which starts the delay timer for the intensifier, opening the gate by pulsing the cathode voltage at the proper time. The intensifier delay uses 24 bits; the gate uses 32, each bit representing a 10 ns time tick. These result in a maximum intensifier exposure of 42.95 s and a maximum delay of 167 ms. The intensifier voltage is refreshed every millisecond for exposures that exceed 1 ms.

Tests with calibrated lamps showed excellent stability between successive spectra, with standard deviations of 0.5–1.0% for the reflection spectrometers and in the 1.5–3.0% range for the transmission spectrometer for 30 spectra taken at room temperature. Integration times for the reflection spectrometers were 5 ms, integrating across the center 16 vertical rows. The gate for the transmission spectrometer was 10 μs, with a gain setting of 2500. The light source was an Energetiq EQ-99 lamp positioned at a distance of 5 m, as described in Sect. 7.1.1.

#### Power Consumption

The SuperCam BU consumes 12.0 W when idling. Additional power is used by the CCDs, the HVPS, and the intensifier when they are operating, bringing the maximum BU power to 19.1 W. The TECs consume an additional 24.0 W.

### Software

**Fig. 30:**
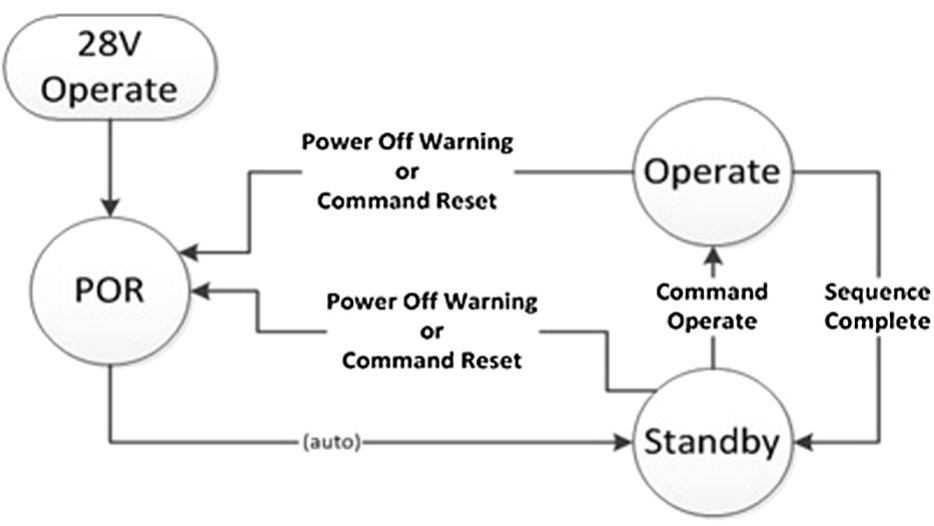
Operating states of the SuperCam BU as commanded by the rover. POR = power-on reset, which is first achieved by providing power to the instrument

The BU flight software consists of a VxWorks 6.7 operating-system kernel and the custom SuperCam application. There are two copies of the kernel and application stored in radiation-tolerant magnetoresistive random-access memory (MRAM). A boot loader resides in a radiation-hard programmable read-only memory (PROM) that controls the startup of the instrument flight software. Figure 30 gives a state diagram of the instrument. The SuperCam BU software starts by executing a boot loader contained in a PROM. Based upon the initialization signal sent to the instrument, the boot loader selects one of the two kernels and applications stored in MRAM. The boot loader performs integrity checks on the software, loads it into synchronous dynamic random-access memory (SDRAM), and starts executing the flight software. Once the flight software begins execution, it initializes itself, performs a built in self-test, then reaches the idle state awaiting commands from the rover compute element (RCE). When a command is received, the instrument goes to an operate state and executes the command. Once the command is complete it returns to the standby state. Only one command can be executed at a time and the RCE must wait until the instrument is in the standby state before sending another command. The only exception is the abort command.

The instrument flight software is designed to handle off-nominal situations. The instrument handles errors in commanding, such as invalid parameters or invalid commands, using reply condition codes as defined by the project. Instrument-specific off-nominal situations are reported using condition codes and status flags as defined in the instrument command dictionary. The instrument maintains two logs in non-volatile memory: an instrument error log and a command history log. If a software processing error occurs, a timestamp and an error code are recorded that indicate the nature of the error that occurred. Both logs can be placed into the instrument’s data buffer and transmitted to the RCE using specific dump commands and the transmit data command as defined in the command dictionary.

During the mission, it will be possible to upload new software, including new kernels, or applications, using the LOAD_MEMORY command as defined in the instrument command dictionary. It is possible to write to any area of memory using this command.

#### Command Handling and Instrument Status

Two key responsibilities of the BU software are to receive and process commands coming from the RCE, and to provide telemetry and data to the RCE. Some commands like CONFIGURE_CCD_REGIONS are executed quickly and only a command reply is sent to the RCE. Other commands like DO_FOCUS, which take much longer to execute, result in the BU sending a command reply followed some time later by a science data frame. Both responses to the RCE contain status flags and a condition code to communicate the instrument’s state.

There are fifteen persistent status flags that the software maintains and are communicated to the RCE as part of every instrument transfer frame. These include flags for MU power, MU direct current (DC) converters, IR spectrometer ready, continuous-wave laser (CWL) ready, laser frequency doubler ready, shutter status, laser stack ready, laser HVPS on, sun safety, BU HVPS state, and CCDs almost ready and ready. Some of the status flags are for MU functions that are explained in the companion paper (Maurice et al. 2020, this journal). If the instrument software detects a fault, the software will alert the RCE by setting the SEND_EVR, SYS_ERROR, or SHUTDOWN status flags depending on the severity of the fault.

In addition to status flags, there are 30 condition codes that the instrument software can use to communicate status with the RCE. These range from indicating there was corruption of the received command, to an error communicating with the spectrometer electronics, or an error communicating the MU.

**Fig. 31:**
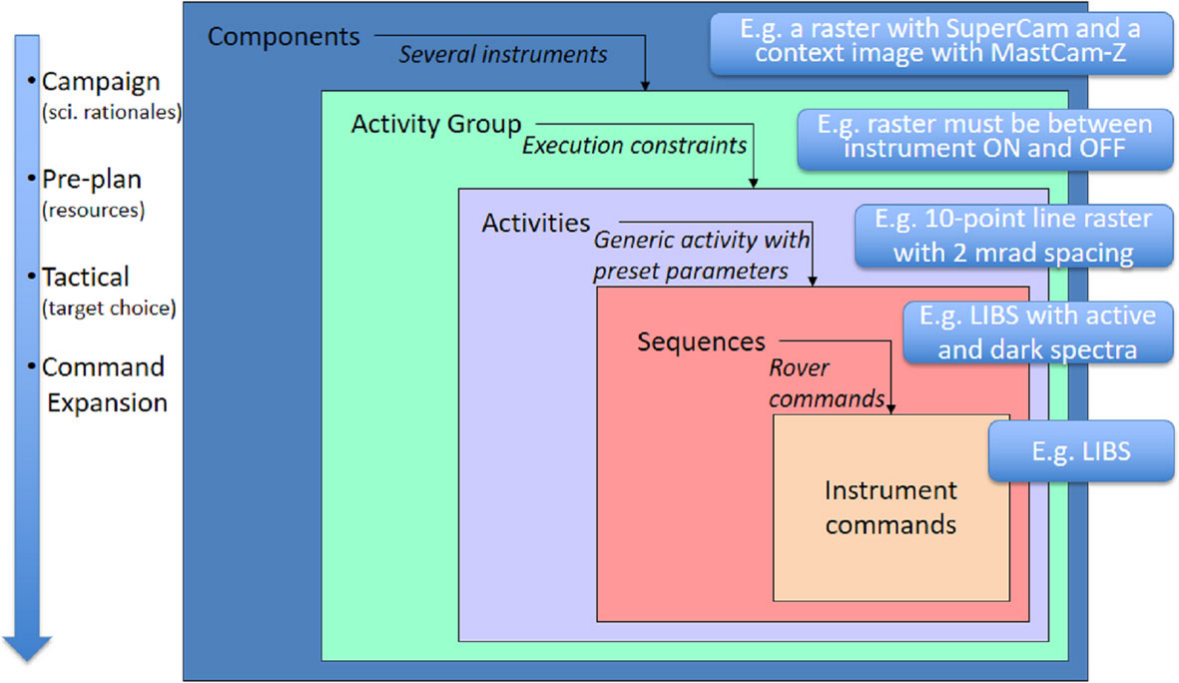
Illustration of the nested nature of the commands that operate SuperCam, spanning from instrument-level commands (I-cmds) to spacecraft-level commands (S-cmds) up to components, which operate more than one instrument

Instrument commands represent one level in a large set of nested levels. Figure 31 illustrates the fact that the rover uses a higher level of (spacecraft) commands, such that a spacecraft command may parse several SuperCam instrument commands. Higher levels exist in the command structure such that a person using the Component-based Campaign, Planning, Implementation, and Tactical (COCPIT) tool for planning the rover operations may be able to use a template to carry out a significant portion of the remote-sensing activity for a given time frame.

The instrument BU software only responds to instrument level commands. Those commands are combined to complete a higher level function. As an example, the following simplified sequence of instrument commands could be used to perform a LIBS collect:

- COLLECT_SOH
- MU_SEND_HOUSEKEEPINGS (housekeeping data generally consists of SOH and status flags)
- CONFIGURE_CCD_VERT_TIMING (this command and the next two configure the vertical and horizontal timing of the CCD exposures, and the specific rows from which to collect data)
- CONFIGURE_CCD_HORZ_TIMING
- CONFIGURE_CCD_REGIONS
- CONFIGURE_HVPS
- CONFIGURE_INTENSIFIER
- MU_DC_DC_CHAIN_ON (turns on the DC converter to enable various MU functions)
- COLLECT_SOH
- MU_SEND_HOUSEKEEPINGS
- MU_CONFIGURE_LASER
- DO_SPECTRA (fires the laser and collects the spectra using the specified laser, intensifier, and CCD configurations)
- COLLECT_SOH
- MU_SEND_HOUSEKEEPINGS
- XMIT_DATA (sends the resulting data to the rover)

The BU software has been designed such that all commands received and all data replies like state of health (SOH) or spectra are assembled into a single generic buffer in the instrument. Data markers and data lengths are used to identify each segment and make the generic buffer parsable. The entire generic buffer is transferred at once to the RCE using the XMIT_DATA instrument command. Section 4.4 provides detail on the formatting of the data product generated by the instrument.

#### MU Commanding, Focus Management, and Monitoring

Other responsibilities of the BU flight software include powering and commanding of the MU, focus management, and monitoring of the MU. Instrument-level commands from the RCE intended for the MU are in many cases just a pass-through for the BU software. However, there are times when the BU must modify command arguments before forwarding the command, and there are times such as the DO_FOCUS command where, as part of the command execution, the BU software will internally generate an MU-specific command. The commands and MU replies are sent over a UART data link between the BU and MU. Similar to the interface with the RCE, the MU indicates status in its command and data replies to the BU.

The BU flight software is responsible for maintaining the state of the MU focus position. This includes keeping track of the focus position in motor steps (table position), converting the table position to distance to the target in millimeters, converting command arguments in millimeters to motor steps, calculating slight focus offsets needed to optimally perform either RMI or spectroscopy, and maintaining the state of the sun-safe flag, indicating that the focus position is not near infinity (Maurice et al. 2020), which would be dangerous for the optics if the sun passed through the FOV. The focus motor position is held in the BU’s non-volatile memory. When the focus motor position changes either due to a manual move or an autofocus, the MU communicates the change to the BU. If the focus position maintained by the BU is deemed to be inaccurate, the proper position can be reestablished by driving the motor to a limit switch.

There are seven components on the MU that the BU monitors to ensure they are within the allowable flight temperature (AFT): laser stack, laser doubling crystal, focus table, CWL laser, infrared spectrometer acousto-optic tunable filter (IRS AOTF), and IRS TEC hot side, and LPVS. The BU checks the temperatures every time an MU_SEND_HOUSEKEEPINGS command is sent, and, if there is no other command being executed, the BU will automatically send the command every ten seconds. If a temperature is found to be out of AFT, the BU will set an error status flag and communicate it to the RCE when the next command is received.

## Details of Operation

### Overview

**Fig. 32:**
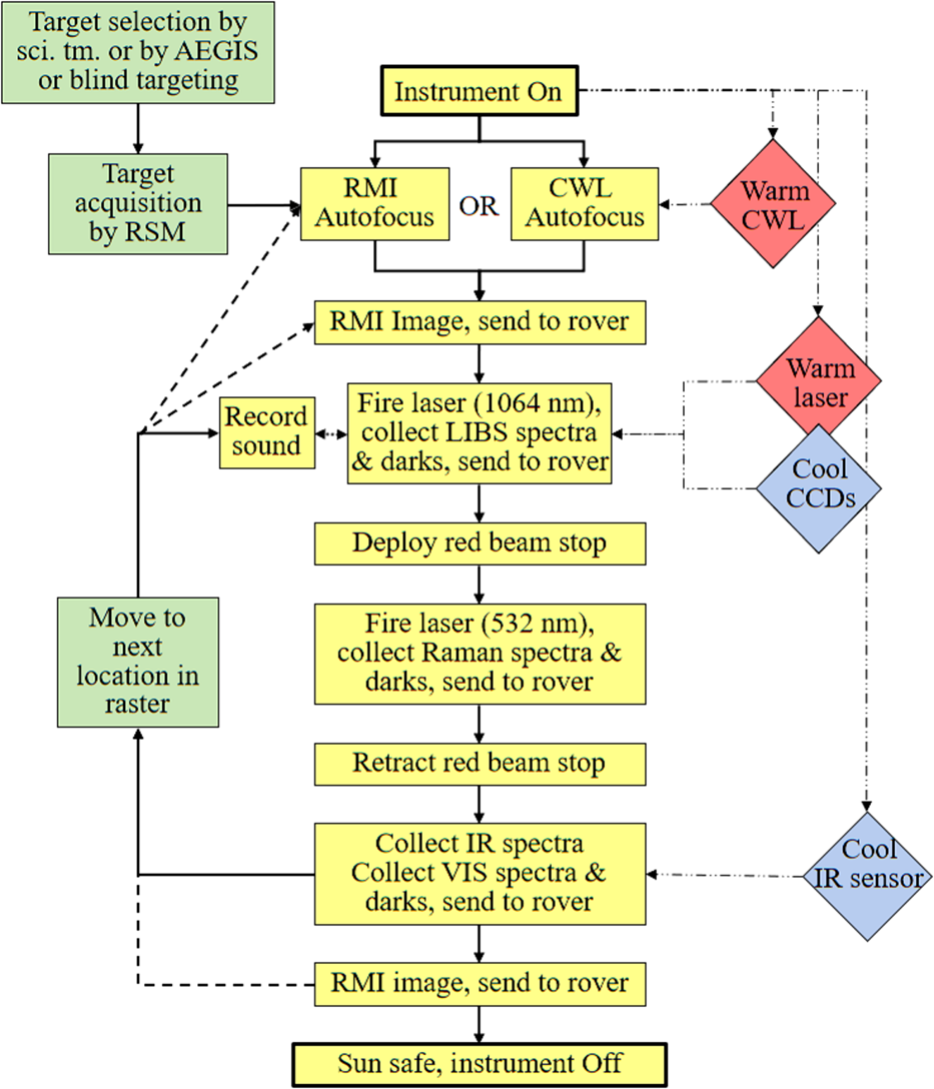
Flow diagram for typical SuperCam operations. This illustration includes all of the spectral techniques, though it is not necessary to use all techniques on a given observation, nor to use the order of taking spectra shown here. See text for description

In general, SuperCam is designed to operate in a similar manner to ChemCam, which takes images and performs spectral analyses on a series of points arranged in a line or grid, referred to as a “raster.” It uses an autofocus routine and takes an RMI image at the beginning of each raster and occasionally thereafter, as required to maintain good focus and to cover the raster area with context imaging. Upon closer look, there are many more details involved in SuperCam’s operations due to the larger number of types of spectral observations and the increased complexity of some of them. Figure 32 shows a general flow diagram for operation of SuperCam. The figure and the description below includes all of the types of spectroscopy, but observations using only one type of spectroscopy (or only imaging), or a different combination or a different order of observations, are all equally feasible.

*Target acquisition, preparation, and RMI imaging*: A target is selected by the science team, and its coordinates are uplinked to the rover as part of the sequence of commands. The target could also be selected by AEGIS (Autonomous Exploration for Gathering Increased Science), an onboard algorithm currently being used on MSL for autonomously selecting ChemCam targets (Francis et al. 2017). It is also expected to be able to perform closed-loop pointing based on pattern recognition by the rover computer operating on Navcam or SuperCam images. Another possible option is to use blind targeting, in which the instrument is pointed at a fixed location on the ground in rover coordinates without any knowledge of the terrain (Maurice et al. 2016). This was done on ChemCam prior to AEGIS. In any case, the rover is commanded to turn SuperCam on and to send it the proper commands for the sequence of analyses. Once SuperCam is on, it is commanded to do several things in preparation for analysis (Fig. 32): Assuming that LIBS, Raman, or VISIR spectroscopy will be done, the TECs will be turned on to start pre-cooling the CCDs. A pre-cooling time of 15–20 minutes is expected to be used prior to the start of taking spectra. If LIBS or Raman spectra are planned, the Nd:YAG laser will be warmed to its operating temperatures of $-15~^{\circ}\text{C}$ for the stack and $-10~^{\circ}\text{C}$ for the frequency-doubling crystal (Maurice et al. 2020). Finally, if the CWL will be used for autofocus, it needs to be warmed to $-10~^{\circ}\text{C}$. The preferred autofocus method uses a series of RMI images, for which no warming is required (Maurice et al. 2020). During thermal equilibration, the RSM turns toward the proper coordinates for the target. Autofocus is the next step, followed by an RMI image centered at the first point of the raster. After the RMI data have been transferred, spectra are taken on the first point.

*LIBS*: Typically, if LIBS plus other spectra are planned, we expect to perform LIBS first, as the LIBS shock wave removes dust from the target, clearing the surface for the other techniques. The laser pit is much smaller in diameter ($\sim250\text{--}400~\upmu \text{m}$; Maurice et al. 2020, this journal) than the FOVs of the Raman, VIS (both 0.74 μrad), and IR (1.15 μrad) observations. At a target distance of 2.5 m, the area of the LIBS laser pit represents $\sim2.5\%$ of the FOV for Raman and VIS observations, and $\sim1\%$ of the FOV of the IR observations. Because of this, the damage caused by the laser is expected to have negligible impact on the other observations (Fau et al. 2019). Instrument commands prepare the laser and spectrometers. Both active (with laser) and dark (background, without laser) LIBS spectra are taken at 3 Hz; the darks will be subtracted from the active spectra on the ground. We plan to use $\sim30$ laser shots for each standard LIBS observation. A separate spectrum is returned for each laser shot, while we downlink only statistics (mean, median, and standard deviation for each channel) from the 30 dark collects. The separate active spectra potentially record changes as the laser profiles up to $\sim100~\upmu \text{m}$ into the sample (assuming 30 laser shots). Microphone recording of the LIBS plasmas is optional; acoustic data are sent to the rover after the recording is finished. After the LIBS data have been transferred to the rover, the next spectral activity can begin.

*Raman/TRL*: For Raman and TRL spectroscopies, changes in the MU include deploying a beam stop to block the LIBS laser path and preparing to use the Pockels cell to cause the laser beam to be frequency doubled, to produce a green 532 nm beam (Maurice et al. 2020). Instrument commands prepare the transmission spectrometer. Both active and dark spectra are taken, usually using 100–200 laser shots at 10 Hz. For Raman spectroscopy, the active spectra can be averaged on board, as no changes to the sample take place as a result of successive laser pulses. In that case, the mean, median, and standard deviation are returned for active and dark collects, each. For TRL, short bursts of laser shots are fired, each burst with a different time delay between the laser and intensifier (see Sects. 4.2.2 and 7.4) to record a time spectrum of the intensity of the laser-induced luminescence. Once the Raman or TRL observation is complete, the LIBS laser beam stop must be removed; there is a time limit of two minutes for its deployment (Maurice et al. 2020).

*VISIR*: For IR observations, the IR TEC in the MU must cool the IR photodiode $\sim50~^{\circ}\text{C}$ below the MU temperature before the observation. VIS spectra are taken using the VIO and transmission spectrometers in the BU, followed immediately by IR spectra (Maurice et al. 2020). The SuperCam IRS is wavelength-scanning, and typically takes $<80~\text{s}$ to acquire a spectrum plus darks. The VIS spectral acquisition is done in several seconds at most.

Typically, once these spectra are taken, the RSM moves to a new point on the same target, developing a raster pattern, usually with point-to-point spacing of 0.5 to 2 mrad ($= 1.25$ to 5 mm at a typical distance of 2.5 m). Planned rasters include $1\times5$ and $1\times10$ line scans, and $2\times2$ and $3\times3$ grid patterns. As the raster proceeds, an additional autofocus may be needed to ensure proper focus is maintained, especially in the case of a vertical raster, where the distance to the target is more likely to change. Another RMI image is taken at the conclusion of a raster, and depending on the angular distance that the raster covers, an additional RMI image may need to be inserted in the middle to ensure imaging documentation of all raster points. Typically an RMI image should be taken every 8 mrad or less to keep the spectral observation points within its FOV (Table 1).

This process can be repeated for successive targets. Several clean-up activities are performed to conclude the observations of one or several targets, shown in Fig. 32. These include moving the focus stage back to the sun-safe position, and performing a controlled warm-up of the IRS TEC. After that the instrument is powered off.

**Fig. 33:**
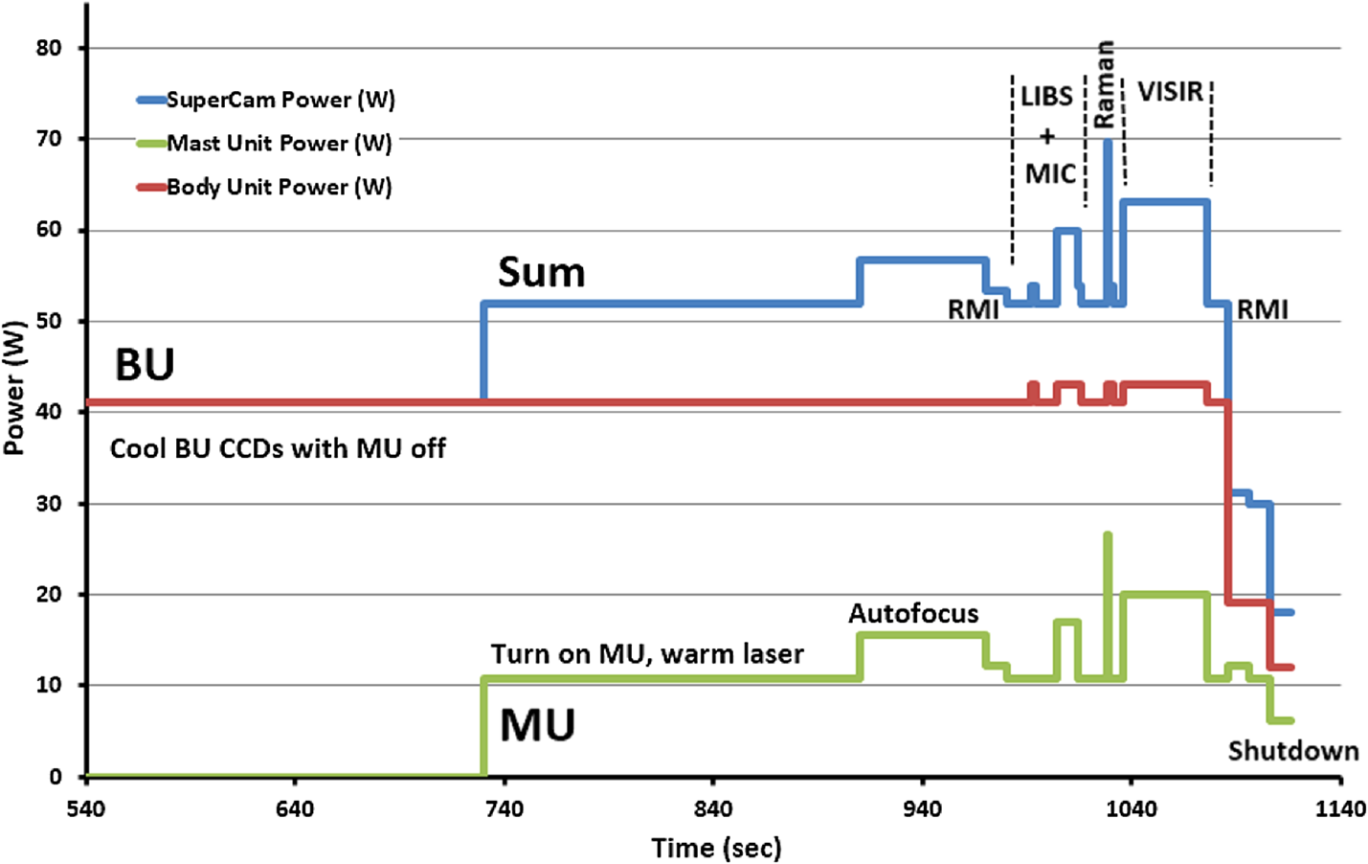
Power profile for one observation point, starting with a power-on and warm-up sequence. Note that the y-axis starts at 540 seconds; in this example the first 12 minutes is used exclusively for BU TEC cooling. After that the laser is warmed, followed by autofocus, an RMI image, and spectral collects for LIBS (30 shots at 3 Hz), Raman (10 shots at 10 Hz), and VISIR spectroscopies prior to a final RMI image and shut-down. Total duration is 18 minutes, 36 seconds; total energy is 13.8 W-hr with a maximum power of 69.7 W, and total data volume is 26.5 Mbytes, dominated by the two RMI images

The three critical resources for Mars operations are time, energy, and data volume. Tracking and optimizing each of these is important for efficient operations. A notional power profile is provided in Fig. 33. In this example, the BU TECs are powered on for 12 minutes before the MU laser warming is started. Activities that follow are CWL autofocus, RMI imaging, LIBS + Microphone, Raman, and VISIR, followed by a final RMI image, and ending with controlled shut-down of the instrument. Just one point is observed in this example, but using several spectral techniques. The turn-on and thermal equilibration takes over 15 minutes, while the analysis portion of the sequences takes a little under four minutes. Given the relatively long time it takes for turn-on and thermal equilibration, it is useful to spend enough time to make more observations once the effort has been made to prepare the instrument. The MU is required to be able to operate two hours at a time. After that, it needs to cool. Certain restrictions apply on the number of laser shots. While SuperCam’s maximum power consumption rate is relatively high, at 70 W, its overall energy budget is relatively low, at under 15 Watt hours in this example, and most of that is for thermal accommodation.

Table 6 provides more details on data volume, duration, and power for some of the raster sequences expected to be used most often. The RMI images have the highest data volume, particularly if not compressed (66.4 Mb; the maximum size for the microphone data is the same). In general, ICER compression (Kiely and Klimesh 2003) is expected to be used for all images, providing a factor of six or more compression relative to no compression. The $48\times1$ IR RMI raster noted in Table 6 uses a mode planned for rapid scanning of an outcrop with just three IR wavelengths; it is supported by RMI images only at the start and stop points. The points in the middle must be co-registered with a Navcam or Mastcam-Z image (Bell et al. 2020, this journal). A microphone raster (Table 6) allows sound to be recorded with the RSM facing three different directions to check directionality, for example, to listen to wind. Thermal accommodation and sun safing of the MU are described in Maurice et al. (2020).

**Table 6:** Duration, energy, and data volume for several typical observations

Activity	Duration (MM:SS)	Energy (W⋅hr)	Data Vol. (Mb)	Notes
Power on & thermal accommodation	15:00	11.8	0.03	BU TEC on 12 min alone; then also IRS TEC cools, YAG & CWL warms 3 min
5 × 1 LIBS, RMI raster	13:23	12.4	107.7	2 RMI w/ ICER comp., 2 AF
5 × 1 LIBS, Raman, TRL, IR, RMI	31:44	31.2	228.8	2 RMI, no comp., 2 AF
48 × 1 IR, RMI raster	15:51	14.7	135.8	2 RMI, no comp., 4 AF (BU TEC cooling not needed)
3 × 1 Microphone	04:19	3.8	36.0	$3\times30~\text{s}$ @ 25 kHz, thermal on; (BU TEC cooling not needed)
Power off & thermal	02:05	1.8	0.01	Includes sun safing

YAG = Nd:YAG laser; comp = data compression; AF = autofocus

### Timing Between Laser, Intensifier, and CCD; Dark Noise

One of the more complicated details that affects the science return and performance of laser spectra from SuperCam is the timing between the laser, intensifier, and CCD. Understanding the operation of the CCDs and intensifier is also important for passive observations. We will start with simplest case and work toward the more complicated ones.

#### Reflection Spectrometers: 2D CCD with No Shutter or Gate

**Fig. 34:**
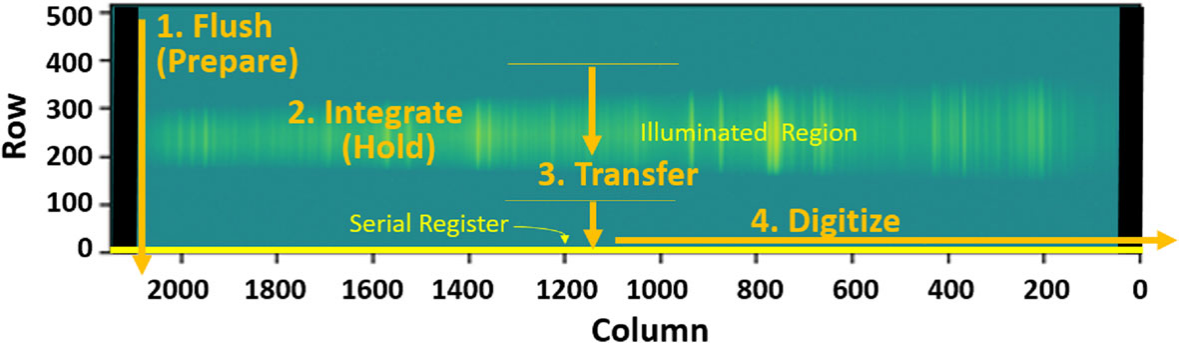
Image of a SuperCam BU CCD illustrating the sequence of events that take place in collecting a spectrum. The spectrum is shown in the center of the CCD. The region from which the light is collected lies in the rows between the two horizontal lines. The serial register that integrates the charge from each column is represented by the yellow row at the bottom. The serial register is read out sequentially to the right

LIBS and VIS spectroscopy use the reflection spectrometers, which have CCDs with two-dimensional arrays of pixels with no shutter or gate. Aside from the timing with the laser for LIBS, which is simple in this case, the main issues are the accumulation of dark current and ambient light. While a 2D image of the spectrum can be read out (e.g., those shown in Fig. 12), the CCDs have a 1D spectral read mode that is more efficient for collecting spectra, as its read process is much faster than reading all one million pixels individually. Instead, columns (channels) are summed into the serial register. The process is illustrated in Fig. 34. When a measurement is about to be made, the CCD flushes the active pixels through a dump drain until the measurement is imminent (Step 1 in Fig. 34). The dump drain is near the serial register. All active pixels have their charges transferred vertically at a rate of 17.99 μs per row. During an exposure, photon-induced charge accumulates in the respective image pixels (Step 2). As soon as the exposure is finished, charges collected in the non-illuminated rows near the bottom are dumped; collected charges in each pixel in the entire array are all moved down in this process. Once they are dumped, charges from the pixels representing the illuminated region are moved down (summed) into the respective 2048 pixels of the serial register (Step 3). As soon as the vertical summing is finished, the serial register is read out, or digitized, pixel by pixel (Step 4), starting at the right side, each one taking 2.50 μs. The vertical and serial transfer rates are adjustable, but are not expected to be changed in flight, as they were optimized on the ground. The entire process, from the time the first charge is transferred from the top of the array (even while dumping the lower pixels just before a collection) to the digitizing of the last serial pixel containing photon data, is $(515 \times 0.01799) + (2148 \times 0.0025) = 14.63~\text{ms}$, not counting the actual exposure time. The first serial pixel is read 5.25 ms earlier than the final one. These time details are important for computing the accumulated dark current. SuperCam’s units of CCD exposure time (“time ticks”) are 34.133 μs. At the shortest setting of a single time tick, used for LIBS, most of the exposure to ambient light occurs as the charges pass vertically through the illuminated region (Fig. 34, Steps 1 and 3, depending on the position of the pixel). This cannot be avoided without a shutter, and experience from ChemCam shows that ambient light in the UV and VIO ranges is low compared to the LIBS signal, and can be accurately subtracted. The CCD time-tick register has 22 bits, so the longest exposure possible is 143.2 s.

The number of rows that are integrated onto the serial register is adjustable. For collection of the maximum amount of light, 200 vertical rows are used. For bright LIBS sources, it is possible to integrate charges from a reduced number of rows. The instrument has been calibrated for settings of 200, 40, and 16 integration rows at the center of the CCD, as noted in Table 3. As indicated near the bottom of Table 3, the charge capacity of each image pixel is $\sim60\%$ less than that of the serial pixels, so going to much fewer rows of integration might run the risk of saturating individual image pixels without knowing it (e.g., without approaching the ADC limit of 65k DN). The calibrations done with reduced image regions may be subject to some variations if the position of the imaging region shifts significantly over the course of the mission. Sections 7.1.2 and 7.1.3 show that the spectrometers have excellent mechanical stability over a large range of temperatures. Occasionally while on Mars a 2D image of the CCD will be collected and used to re-check the number and positions of the rows receiving light. Even though the dump drain is used just prior to the exposure and read-out, the first row that is read from a 2D CCD image has high noise, and should be discarded. This same phenomenon also has a minor effect on the first 1D spectrum that is read in a series of reads, such as for a laser burst.

Use of reduced integration rows works for LIBS, where the illumination is transient within the exposure period. It does not work for passive spectroscopy, where the illumination is continuous, due to the lack of a shutter. The use of reduced integration rows will not be needed for reflectance spectra of rocks, which are quite dim in the UV and VIO ranges. The UV reflectance is too dim to generally collect useful data, and optimal exposure times for the VIO range are many milliseconds.

#### Transmission Spectrometer Chronology

For the transmission spectrometer, the intensifier acts as a shutter. However, reading the CCD becomes more complicated due to the fact that the CCD has three regions to digitize separately (Fig. 12). Additionally, light emission from the phosphor of the intensifier is not emitted instantaneously, so time must be added to collect all of the phosphorescence from the intensifier in order to avoid smearing its light across the different bands as the CCD is being read. Additional complication comes from the short time duration of the Raman signal. We will start our discussion with a comparison between LIBS, Raman, and TRL, and then apply this information to the operation of the intensifier and CCD.

For normal LIBS observations with the transmission spectrometer, the intensifier gate is opened at the same time as the laser is fired, and it is closed after 10 μs, at which point all of the atomic emissions and essentially all of the molecular emissions have been collected (Sect. 7.2.3). The intensifier allows more specialized studies to be done, such as greatly amplifying the signal to study a very weak emission line, or changing the delay or gate to discriminate against some species to more easily detect other species.

**Fig. 35:**
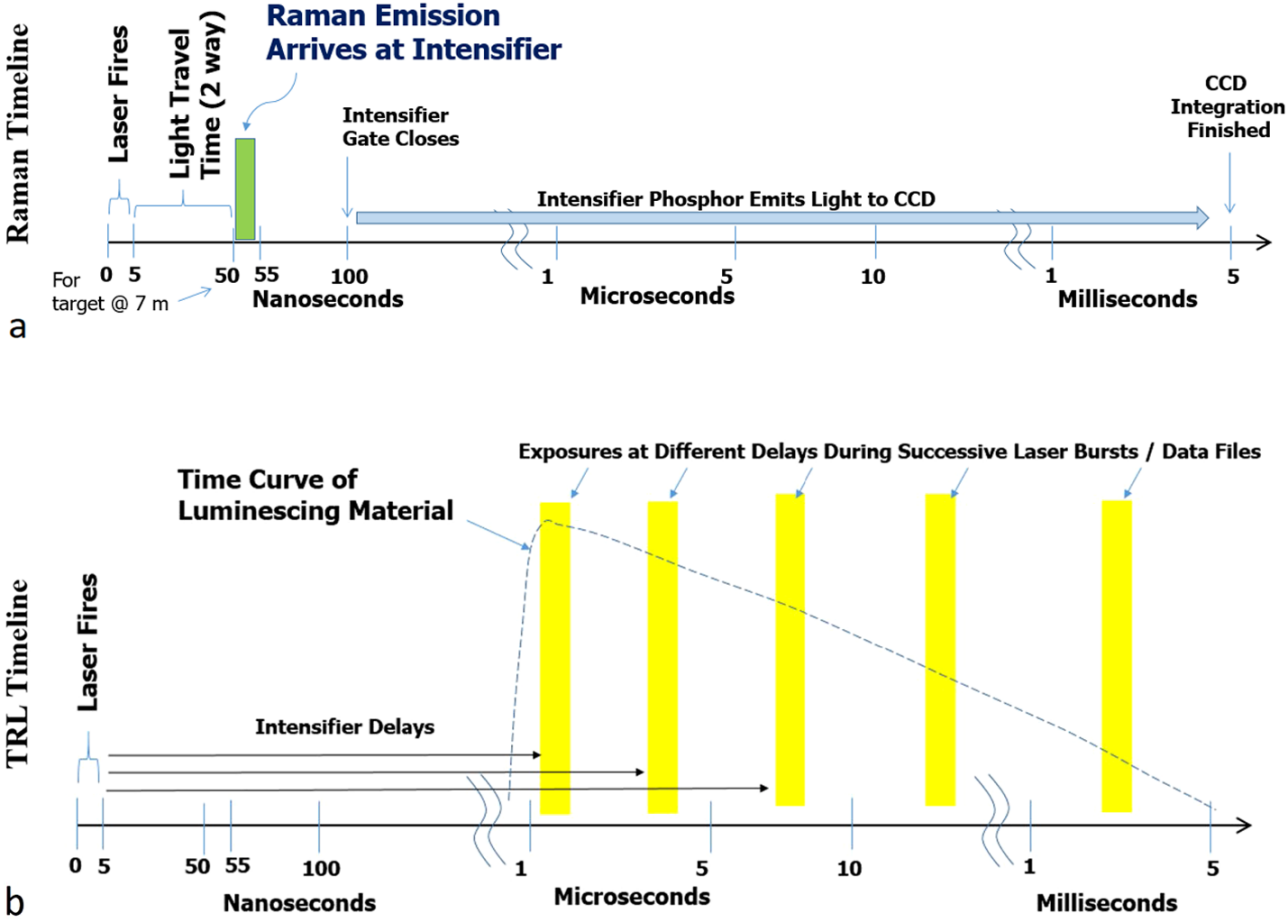
Illustration of the timing sequence of the ICCD in the transmission spectrometer for collecting Raman emission (**a**). The intensifier gate closes at 100 ns. The phosphor emission from the intensifier continues for several milliseconds, so the CCD does not transfer its signal until 5 ms after the gate closes. The TRL data collection scheme is also shown (**b**). In this case, data are collected at up to five different delay times to determine the time dependence of the luminescence. Each exposure at a different delay requires a separate laser burst and collection by the CCD

Figure 35a gives a schematic view of the transmission spectrometer operation for Raman spectroscopy. The intensifier duration is set to 100 ns to reject most mineral luminescence, which emits over a much longer timescale, as well as to minimize ambient light. If Raman observation distances are quite long ($\geq6~\text{m}$), attention needs to be paid to the light travel time, to avoid cutting off the Raman signal. As described in Sect. 3.3.2, the start of the intensifier timer is at a somewhat arbitrary time shortly before the firing of the laser. Because of this, the intensifier delay is normally set to $\sim65$ time ticks for close-range Raman observations (each time tick is 10 ns).

Figure 35a shows another important detail in the operation of the intensifier-CCD pair. The light excited by electrons hitting the phosphor screen at the output end of the intensifier continues to be generated after the gate is closed. Hoess and Fleder (2000) show that P43 emission drops to 0.2% of its original intensity by 5 ms. The CCD must wait to transfer its signal to avoid smearing the last of the phosphor emission onto the wrong wavelength bands. So a CCD integration time of 5 ms beyond the closure of the intensifier gate is planned for all transmission spectrometer observations. This also includes LIBS, TRL, and VIS spectroscopy.

For purposes of understanding the dark current accumulation in various parts of the spectrum, Table 7 shows the timing of the various steps in reading and digitizing the CCD of the transmission spectrometer.

**Table 7:** Transmission spectrometer CCD operation steps

Action	1/rate (μs)	Number	Duration (ms)	Total (ms)
Integration^a^	5000.00		5.00	5.00
Dump unexposed rows^b^	17.99	92	1.65	6.65
Transfer **red** band to serial register	17.99	70	1.26	7.91
Digitize serial register	2.50	2148	5.37	13.28
Dump unexposed rows	17.99	74	1.20	14.61
Transfer **green** band to serial register	17.99	120	2.16	16.77
Digitize serial register	2.50	2148	5.37	22.14
Dump unexposed rows	17.99	22	0.40	21.54
Transfer **orange** band to serial register	17.99	128	2.30	24.84
Digitize serial register	2.50	2148	5.37	30.21

^a^Does not include intensifier exposure duration, which should be added on for long gates

^b^Calibration has been carried out for two settings of the red band. The one shown here, with 70 rows, cuts off some of the light and provides higher resolution

TRL operation is illustrated in Fig. 35b. Each TRL observation uses a series of laser bursts aimed at the same point on a target. For each laser burst, a different delay is used for the intensifier gate, probing different timescales for luminescence generation and decay. As will be illustrated in Sect. 7.4, luminescence generated by different sources can vary in duration by orders of magnitude, and these temporal differences are important in identifying the species (Gaft et al. 2015).

### Misc. Operating Modes

SuperCam permits CCD spectral collection and transmission in several different modes for active spectroscopy.

- Single-pulse collection: The simplest and most frequently planned is single-pulse collection, in which the light returned from a single laser pulse is collected from the CCD(s) as a single one-dimensional spectrum. This is done routinely for LIBS, where each spectrum provides unique depth-sensitive information as the laser probes deeper into the target.
- Co-adds on chip: For Raman spectroscopy, with its weak signal, the light from multiple laser pulses can be collected on a single one-dimensional CCD exposure. The intensifier still gates each pulse (with no light transmitted from the input side of the intensifier in between), but the charges are not read from the CCD until after several pulses. For a large number of laser shots, a hybrid approach can be used, such as firing 100 laser pulses and collecting 10 spectra of 10 pulses each (ten one-second exposures at 10 Hz) to avoid saturation. The advantage of co-adds on chip is that by reading the CCD fewer times, the fixed noise associated with each read (“read noise”) is reduced for the ensemble, but this is done at the expense of increased dark noise which accumulates as the CCD is integrating. The dark noise depends sensitively on temperature and radiation damage.
- 2D spectra (Fig. 12): Full images can be made from the CCD. This will only be done for diagnostic purposes, as the time involved in these full reads results in orders of magnitude more dark noise and data volume.

LIBS optical spectra can be combined with acoustic spectra, thanks to SuperCam’s microphone (Maurice et al. 2020). The purpose of the LIBS + microphone pair is to study physical properties of the targets from the evolution of the sound intensity with successive laser pulses (Murdoch et al. 2019; Chide et al. 2019, 2020; Maurice et al. 2020). There are two modes that include acoustic recording of LIBS. One is with a continuous recording during the laser burst. The other is with the microphone in pulsed mode, in which a 60 ms recording is made for every laser pulse. This pulsed-recording mode reduces data volume while still capturing the laser pulse and any associated echoes. More details of the microphone operation are in Maurice et al. (2020).

Most of SuperCam’s imaging is done with either a simple image or a high-dynamic range (HDR) mode, as described in Maurice et al. (2020). One other mode has been developed to compensate for the very narrow depth of focus of the RMI camera. The BU can command a z-stack to be performed, in which a series of several images (3–7) are collected at slightly different focus positions, moving successively in the close-focus direction. The images are sent to the BU, where they are combined into a single image. Either a $3\times3$ or a $5\times5$ box filter (comparing either the 8 adjacent pixels or the 24 nearest) can be applied to determine which image has the highest contrast for each pixel, providing a Laplacian of Gaussian; the algorithm operates on a single color (currently set to red and $3\times3$). The algorithm is relatively simple, to avoid lengthy processing time. For example, the algorithm does not re-register pixels. As a result, the range of focus is somewhat limited. A three-image stack takes $\sim220~\text{s}$, while a seven-image stack takes 350 s. The algorithm is limited to seven images by a time-out at 360 s. ChemCam has performed some z-stack RMI images (e.g., Le Mouelic et al. 2015); the advantage with SuperCam is the ability to make them onboard, resulting in significant data-volume savings at the expense of data-processing time.

### Output Data Format

Raw spectra are read from the SuperCam spectrometers sequentially. First the VIO data are read, followed by UV data, and finally, transmission spectrometer data. For the latter, the bands are recorded in the order in which they are digitized: red first, then green, and lastly orange. The data streams include the 100 blind serial-register pixels in addition to the 2048 active serial pixels (Table 3 and Sect. 3.1.3), so the number of channels per spectrum is 10740 for LIBS, 6444 for Raman and TRL spectroscopies, 8592 for VIS spectroscopy, and 256 for IR spectroscopy. The SEND_DATA spacecraft command is generic in that it requests any data that is in the generic data buffer. The instrument is thus flexible in the amount of data sent. Flight sequences are specific in the order that commands are sent, such that data are sent after each spectral observation at a given point. Image data are held and sent separately from the other data, which includes all the spectral data, the command history with their input arguments, the microphone data and the SOH. Binary data are sent to the ground (generally once to several times a day from the rover) encapsulated into data products (DP), which are parsed, restructured and complemented with ground information. The resulting products are the so-called experimental data records (EDRs). EDRs for SuperCam are in the form of flexible image transport system (FITS) files (Wells et al. 1981). This file type is particularly well suited for SuperCam due to the different types of data (images, spectra, acoustic data, SOH) that the instrument can collect. Depending on the content of the original DPs, EDRs contain a combination of science data (spectra, RMI, acoustic), SOH data, and auxiliary data (e.g. the position coordinates of the RSM) or the command history. EDRs containing science data will be processed and calibrated by a specific pipeline, and the resulting products are called clean data records (CDRs).

## Model Development and Environmental Testing

The overall plan of development of SuperCam emphasized early validation of new technologies, particularly the transmission spectrometer and its HVPS as applied to Raman spectroscopy, followed by an environmental qualification model (EQM) and flight model (FM). To facilitate the early validation, an engineering development unit (EDU) was developed and tested. Its MU consisted of a laser and associated electronics, telescope, RMI, and an infrared spectrometer that was fiber-coupled to the telescope. The EDU BU consisted of electronics, demultiplexer, reflection spectrometers, and a transmission spectrometer. TEC cooling of the EDU CCDs was done with commercial units via a non-flight copper bar from the CCDs. The EDU transmission spectrometer had only two spectral bands instead of the three in the EQM and FM, and so it had low resolution. Integrated testing of the EDU SuperCam instrument was done in the spring of 2016 at LANL, and was used to verify a number of details, including (a) overall coupling of the BU and MU, including the timing between the laser and spectrometers; (b) performance with the reflection spectrometers that was comparable to ChemCam; (c) basic Raman and TRL functionality and throughput; and (d) scientific studies of TRL spectra (Ollila et al. 2017). The latter was feasible because the luminescence peaks are often broad, and are thus relatively insensitive to the resolution of the spectrometer, which was not optimized at this early development stage. The EDU did not use flight-qualified electronic parts, and it was not built for environmental testing. Environmental testing was instead only carried out on new sub-assemblies, such as the transmission spectrometer, before integration into the BU.

After the EQM was built, the MU EDU was stripped of its laser and infrared spectrometer. A special BU simulator was reconstructed from spare parts, with no optics. The two units were delivered to JPL in 2017 as the SuperCam Test Unit (TU), which supported verification and validation (V&V) activities and operations training. This unit will be mounted on the vehicle system test bed (VSTB) at JPL and will be maintained by the team for the duration of the mission on Mars. The BU EDU is planned to be used as a calibration model in the laboratory. A new MU will be rebuilt for that.

The most significant change between the EDU and EQM BU was the transmission spectrometer (Sect. 3.1.4), which was redesigned with the dichroic beam splitter and compound grating to yield three wavelength bands instead of the two that the EDU had. This improved the resolution to meet the $12~\text{cm}^{-1}$ requirement. The EQM BU underwent environmental testing in LANL, while the EQM MU underwent testing in Toulouse before integrating the units. Testing of the BU identified three issues: slight motion of the reflection spectrometer optics during vibration and shock, under-performance of the TECs, and a component failure in the HVPS after its cold thermal cycle. The reflection spectrometers were only slightly redesigned from ChemCam. For both instruments the optics are held in tension against a three-point mount. The problem was never seen on ChemCam, and for SuperCam, the only occurrences were at the qualification levels, with extremely minor movement at the flight-acceptance instrument vibration, and no movement during rover shock or vibration. The HVPS component failure led to re-analysis of the mechanical stresses on the components, and some re-positioning of components. The TECs were not modified on the FM relative to the EQM, but a number of improvements were made for the flight TEC cooling system to insulate it further from the environment and to spread the heat to the RAMP better. During testing of the EQM BU, the wavelength dependence on temperature for all three spectrometers was shown to be much less on SuperCam (Sect. 7.1.3) than on ChemCam (Wiens et al. 2012).

The EQM is fully functional. The EQM BU and MU required some rework post-FM-delivery to match the FM performance. They will be used as a calibration model, particularly to complement the LIBS spectral database of known compositions, and to acquire a library of time-resolved Raman and luminescence signatures. Thus the EQM is critical to achieving SuperCam’s overall scientific mission.

The flight BU has very few changes relative to the original EQM other than those mentioned above. Thermal, Mars-pressure testing of the FM identified one new issue: The intensifier created flashes of light at random times when at Mars pressure. This was due to an imperfection in the multi-channel plate and surrounding potting. Potting of the tubes was most successful on the EQM tube, and so that tube is being used for flight. It was also found that the EQM intensifier tube had a small tendency to create flashes at Mars pressure in $\text{N}_{2}$ gas. Nitrogen is the test gas of choice, as it is easier and safer to use in a thermal chamber, and represents an over-test relative to the Martian atmosphere, for which breakdown does not occur as easily. The flashes were likely due to charge buildup on the output window of the intensifier due to electric field leakage through the unit. Extensive testing was carried out in Mars gas, showing that there were no flashes under Mars conditions using normal operating procedures.

As part of the requirements for delivery the FM BU underwent a series of environmental qualification tests including random vibration and thermal vacuum, which were conducted using Los Alamos facilities. Random vibration testing was performed in each of the three axes at protoflight levels for one minute per axis. After vibrating each axis a visual inspection was performed and the accelerometer data was reviewed to assess any mechanical change to the unit. A comprehensive functional test was also conducted before the test, between axes, and after the test to determine any performance change after exposure to the vibration environment.

The BU then underwent 204 hours of thermal vacuum testing. The chamber supported bulkhead feedthrough connectors that allowed power, command and telemetry signals, and an optical fiber to be coupled from support equipment outside the chamber to the unit in the chamber. When installed on the rover the SuperCam BU has its baseplate attached to the underside of the RAMP such that the thermal sink is above the CCDs. The orientation affects the performance of the heat pipes, so we needed to mimic the BU orientation in the rover for realistic thermal tests. For this, a secondary platen was mounted on legs inside the thermal vacuum chamber with its mounting surface facing down. The baseplate of the BU was then bolted to this surface. To complete the test setup a number of thermocouples were attached to the unit.

During thermal vacuum testing the BU completed three thermal cycles across its protoflight temperature range of $+60~\text{C}$ to $-50~\text{C}$. The first thermal cycle was conducted at high vacuum below $1\times10^{-5}~\text{Torr}$ to replicate cruise conditions. The remaining two cycles were conducted at Mars pressure ($\sim7~\text{Torr}$) using gaseous nitrogen. Each thermal cycle included long soaks at the maximum and minimum temperatures. During the soaks and at various intermediate temperatures, testing was conducted to verify the functionality of the electronics and the effectiveness of the TECs to cool the CCDs, and to assess the performance of the spectrometers. During the high-vacuum cycle the decontamination heaters were tested to verify their ability to maintain the optics at the desired temperature.

Earlier in the project, pyro-shock testing was performed on the EQM BU using Los Alamos facilities. Results from this test were used to approve the BU FM without testing, limiting potential mechanical stress on the flight model. Additionally, an electromagnetic compatibility and electromagnetic interference (EMC/EMI) test was performed using the EQM BU coupled with the EQM MU at a JPL facility. Results from the EMC/EMI testing showed that the BU exceeded maximum radiated signal levels in a couple critical frequency bands. This was addressed on the FM by adding some additional EMI shielding to specific areas of the unit. Final testing of the instrument on the rover verified that SuperCam meets requirements, and can be used during UHF communications, which is an improvement over ChemCam.

## Performance Testing

The test and validation campaign of SuperCam was constrained by the availability and capabilities of the units and models, as described below. Table 8 shows a summary of the testing of various models and configurations. The EDU was useful for testing of functionality, despite the fact that some of the EDU subsystems were not fully flight-like, and some were not present. For example, the microphone was not part of the EDU, and as mentioned, the transmission spectrometer only provided low resolution. The EQM was a better testbed, as it was flight-like in nearly every sense. It provided a stable platform for studies of Raman performance, although a few aspects of its performance (alignment, focus at cold temperatures) were not as good as that of the FM. Thus, Raman and TRL studies could be made at close distance (e.g., 2–2.5 m), but not at distances $>5~\text{m}$. Raman and TRL observations were also carried out with the FM units, often re-testing samples analyzed with earlier models.

**Table 8:** Summary of testing of the SuperCam models

BU	MU	Location of testing	# Data files	Study Raman, TRL	Validate functionality	Cross calibration	LIBS spectral library	Autofocus offsets	Realistic thermal environment	Flight fiber
EDU	EDU	LANL		TRL	X					
EQM	EQM	LANL	5534	Raman	X					
FM	EQM	LANL	3100		X	X	X			
EQM	FM	IRAP	743		X	X		X		
FM	FM	JPL	1119		X	X			X	X

For the flight model, a problem arose in the fall of 2018 that required a complete re-build of the MU (Maurice et al. 2020, this journal). Because of that, the flight MU was not ready until after the required delivery date of the BU to the rover. Validation of the BU in a realistic environment, development of a LIBS spectral library, and initial calibration of the instrument response function (sensitivity vs. wavelength, or IRF) of the system had to be done on a combination of FM BU + EQM MU. To validate critical MU functions such as the autofocus offsets (offsets as a function of temperature for different relative focus points for LIBS, RMI, and the CWL autofocus mode; Maurice et al. 2020), after delivery of the FM BU to the rover, the EQM BU was sent to Toulouse to support testing in the configuration of EQM BU + FM MU. Finally, as shown on the right side of Table 8, the last flight portion of the instrument was the FOC, the flight version of which never left JPL. So when the BU and later the MU were integrated onto the rover, all of the flight parts of the instrument were finally together. Because of the short time available in the final flight configuration, results (Sect. 7) will be presented from various configurations, as appropriate to represent the overall performance of the flight instrument. Because we could cross-check the FM units with the EQM units, and because the EQM units were generally very similar to the flight units, we believe the differences in characterization relative to the complete flight unit were relatively minor and limited to those aspects noted here.

### Flight-Model Body Unit + Engineering Qualification Model Mast Unit Testing at LANL

From 6 to 23 April 2019, the FM BU was tested in its final configuration with the EQM MU for performance and software validation, and to develop spectral libraries. Both the BU and MU were housed in a thermal chamber with dry nitrogen at ambient pressure, and at $-10~^{\circ}\text{C}$ to optimize the laser energy. For LIBS, a Mars sample chamber pressurized to $5.8\pm 0.2~\text{Torr}$ with $\text{CO}_{2}$ was used to simulate the Mars atmosphere, which affects the plasma properties. During observations of samples in the Mars chamber, the window of the instrument thermal chamber was removed, and a pipe was installed between the instrument thermal chamber and the window of the sample chamber. In this way, there was a single window between the instrument and the samples, as will be the case on Mars. Raman, TRL, RMI, and VISIR observations were made with the instrument-chamber window in place and the samples in open air.

The primary purpose of the LIBS observations was to establish a library of spectra of a diverse suite of geological materials, mostly at one distance, and then a smaller library at several distances. Experience with ChemCam has shown that a large number of standards are needed for quality calibration using multivariate methods for all of the major elements and a mixture of methods for minor and trace elements (Wiens et al. 2013; Clegg et al. 2017; Payré et al. 2017). Depth profiles were also produced using a large number of laser shots (Sect. 7.2.4). Table 9 gives a summary of the LIBS observations. Three observations were made of each standard, with each observation consisting of 30 laser pulses. Each laser observation has a corresponding dark spectrum of 30 collects. Standards were loaded in the sample chamber on a turret that holds approximately fifteen 30-mm diameter pressed powder pellets, which is the physical form of the LIBS standards. With each turret load, a small titanium plate was installed and observed. Cross comparison of the Ti observations from one turret load to another can be used as a check against problems with focus, clipping of the laser beam in the pipe, laser energy, or ambient pressure. All of the LIBS targets were observed at 2.85–3.00 m distance. The SCCTs were observed again at 1.55 m (the distance of the SCCTs on the rover) and at 4.25 m. A full list of the standards and the calibration parameters derived from the library will be published elsewhere.

**Table 9:** Numbers of unique samples of rocks, minerals, and mineral groups observed by the FM BU and EQM MU during the validation period

Rock/mineral type	LIBS	Mineral/type	Raman	VISIR
Andesite	14	Carbonate	17	5
Trachyte	4	Silicate	12	6
Anorthosite	6	Plagioclase-feldspar	9	1
Basalt	33	Sulfate	8	4
Pyroxene	7	Olivine	6	1
Plagioclase-feldspar	3	Phosphate	5	
Olivine	6	Phyllosilicate	4	9
Dolerite	3	Pyroxene	3	1
Gabbro	8	Serpentine	2	3
Norite	5	Mica	1	
Syenite + granite	5	Amphibole	1	
Carbonate	8	Diamond	1	
Sulfate	7	Fluorite	1	
Fe, Ti, oxides, banded iron	8	Metal oxide	1	9
Mn oxides	13	Sulfide	1	
Metamorphic, Archean sediments	49	Nitrate	1	
Clastic sediments including shale	91	Perchlorate	1	1
Phyllosilicate (kaolinite, smectite)	6			
Miscellaneous other	31	Organic, mineral mix	51	15
SCCT	25	SCCT	14	13
**TOTAL**	**332**	**TOTAL**	**138**	**68**

Raman and VISIR spectra were taken to validate the protocols and to obtain representative spectra from the instrument. Table 9 lists the different unique targets that were observed by these techniques that were carried out with the FM BU + EQM MU. A few TRL spectra were also recorded. In addition to pure minerals, some mineral mixtures were tested, as well as mixtures of organic species with inactive binder materials. As mentioned above, many more such observations were carried out with the complete EQM instrument, especially for TRL spectroscopy (Ollila et al. 2017). The results are presented in Sect. 7.

### Engineering Qualification Model Body Unit + Flight-Model Mast Unit Testing in Toulouse

The rover schedule required the BU to be installed first, leaving several more weeks to complete and deliver the MU. During this time the EQM BU was sent to France to facilitate final testing of the FM MU. The primary objectives were to verify the communication between the two units and to perform safety checks by the BU software that included monitoring of MU temperatures and setting the correct status flag if out of range, and verifying that the BU set the appropriate motor speed, number of steps, distance to limit switches, pumping current, and laser rest time (30 s) between consecutive bursts. We also checked that the BU reported the correct condition codes, and the sun-safe status in all configurations, even if communication with MU was lost.

The secondary objectives were to verify instrument functionality, and to characterize some key parameters that are unique to the FM MU. These include CWL-to-LIBS and CWL-to-RMI focus-offset algorithms (Table 8) that were subsequently coded into the FM BU software. The test also checked the quality of the LIBS focus and the number of spectrometer CCD rows to use to avoid saturation at short distance. Some microphone noise tests were also performed.

Finally, this configuration provided an opportunity to acquire some additional calibration data which could be used along with data from the EQM MU + FM BU tests at LANL (see Sect. 6.1). LIBS spectra were acquired at 1.56 m and 3 m, and compared to ChemCam spectra on Mars, confirming the comparative results to be presented in Sect. 7.1. A few tests at 7 m showed better SuperCam performance than ChemCam at that long distance, but the actual long-distance capabilities will have to be shown on Mars. Time-resolved Raman spectra were also obtained from gypsum. TRL spectra were obtained from apatite and showed REE and Mn2+ detections at $\sim100~\text{ppm}$ levels for a 0.5 ms integration window.

After the successful conclusion of these brief tests, the FM MU was delivered to the rover.

### Testing on the Rover

Extensive performance testing of the end-to-end FM SuperCam instrument on the rover took place in several campaigns. The general details are given in Table 10. Essentially all of the functions of SuperCam were tested at least once (except RMI z-stack). Alignment tests were done before and after dynamic and thermal environmental testing to verify that the FOV of the transmission spectrometer is well aligned (at the proper temperature) within the green laser beam, as needed for Raman spectroscopy (Maurice et al. 2020). These were done using a Spiricon camera and neutral density filters. Checks were also made of the CWL and LIBS laser beam positions relative to the RMI FOV. Observations were made to support modeling of the pointing of SuperCam relative to Mastcams and Navcams (parallax and offset). This camera model is critical to being able to command the correct position to hit a target with SuperCam based on the location of the target in the Navcam or Mastcam images, all from a given rover position. RMI images taken in support of the camera model and other testing revealed a small number of unexpected particles near the focal plane of the RMI imager. Investigation indicated that these particles reduce the light only slightly in small areas of the image, and they can be removed from the images as part of the flat-field correction.

**Table 10:** Testing of SuperCam on the rover in 2019 to early 2020; see text for explanation

Campaign	July	August	STT	EMI/EMC	December	January
Tests	CWL autofocus	RMI autofocus	Autofocus	Instrum. on	Code update	Dark collects
	RMI autofocus	IRF	LIBS	RMI	Alignment	RMI HDR
	Alignment	Thermal control	LIBS + MIC		IRF	IRS side B
	LIBS (3 m)	TRL sweep (5 m)	MIC alone			LIBS + MIC (pulsed mode)
	Raman (3 m)	Passive VIS	Raman			
	RMI (camera model 2, 3, 5 m)	98-point IR scan	VISIR			
			RMI			
			2D spectra			

Additional testing (survival only) included rover-level vibration and RSM-release shock

The system thermal test (STT; Table 10) covered $\sim2$ weeks in October 2019 with the rover in a large thermal/vacuum chamber at a range of temperatures to simulate Mars. Most of the testing was done at $\sim10~\text{Torr}$ of $\text{N}_{2}$ to simulate Mars thermal conditions, with some testing to simulate the cruise conditions (vacuum). Although the RTG power supply was not installed on the rover, the RAMP was heated as if it were present, to provide realistic temperatures for the instruments and other components in the rover. Two sets of plates with geological targets were mounted in the chamber. One was on the ground near the rover (2.6 m from SuperCam) and another plate was 4.6 m from the instrument, hanging on the wall. The SCCTs on the back of the rover constituted a third set of targets. In addition, SuperCam took one RMI image of the targets installed by the SHERLOC team. STT provided the first realistic test environment for the IR spectrometer, and also for the LIBS + microphone combination. All of the other main observation modes were carried out in STT: LIBS, Raman/TRL, VISIR, and RMI. Lastly, all end-to-end thermal control loops and associated hardware were confirmed in STT.

The rover-level electromagnetic interference and compatibility (EMI/EMC) tests complemented other EMI/EMC testing done earlier at the instrument level. The test validated that SuperCam does not cause interference with the ultra-high frequency (UHF) antenna, and so the instrument can be operated at the same time as UHF uplink and downlink.

December 2019 testing of SuperCam consisted of a minor software update and repeat observations of IRF and alignment. The latter two were important to verify that rover vibration, shock, and thermal testing had not adversely affected the optical system. The final IRF is given in Sect. 7.1.1. In January 2020, true “darks” were obtained by acquiring dark spectra with the lid on. Finally, we tested the backup IRS photo-diode, the RMI HDR mode and the microphone pulsed mode (Table 10).

## Results

This section describes results of the integrated SuperCam instrument. Some additional results in terms of IR spectra, RMI images, and acoustic results can be found in the companion paper (Maurice et al. 2020), as these techniques are complete at the MU level and did not require an integrated instrument.

### Overall Optical Properties

We present some of the overall optical properties here. Radiometric information on the RMI is presented in the companion paper (Maurice et al. 2020). The IRF of the IR spectrometer is given in (Royer et al. 2020).

#### Instrument Response Function (IRF), BU Spectrometers

Two different calibrated lamps were used to determine the IRF across the spectral range from 245–853 nm. For the violet, green, orange, and red spectral ranges of the transmission and VIO reflection spectrometers, a calibrated Labsphere integrating sphere and lamp assembly was used. This lamp provided uniform radiance generally at the 2% level or better across its aperture ($\sim100~\text{mm}$) and for reasonable angles of a few degrees. The same lamp was used to calibrate ChemCam (Wiens et al. 2012). As a second source, a calibrated Energetiq EQ-99 laser plasma discharge lamp was used across the entire spectral range. The advantages of this lamp include a significantly brighter output at all wavelengths compared to the Labsphere, and a sufficient signal in the UV to allow the calculation of IRF. Overall, it is a good broadband source, but its output contains a few emission peaks, such as in the red range, and also $\sim455~\text{nm}$ in the VIO range, that are not fully described in the manufacturer’s calibration data. Use of the Labsphere allowed an independent check of these regions. Other than the plasma emission peaks, cross checking of results obtained with the Labsphere and with the EQ-99 showed close agreement, within $\sim2.5\text{--}3\%$ across all but the UV spectral range, which is not covered by the Labsphere.

**Fig. 36:**
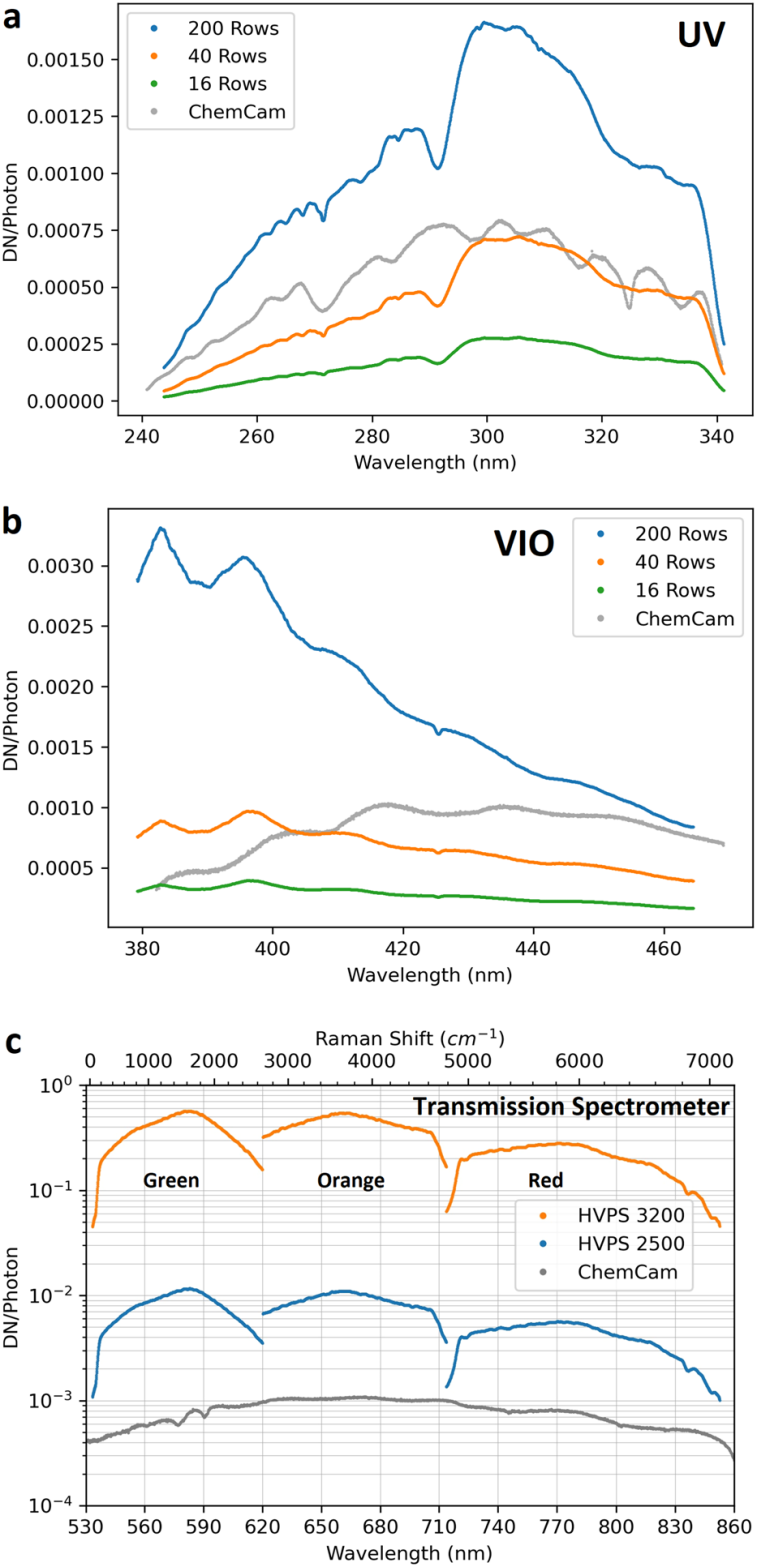
Preliminary instrument optical response functions for the SuperCam BU UV (**a**), VIO, (**b**), and transmission (**c**) spectrometers. The units are digital numbers (DN) per photon incident at the telescope aperture. Data were taken with the instrument mounted in the rover. For the reflection (UV, VIO) spectrometers, read-out of fewer CCD rows results in a lower optical response that can be used to avoid saturation for nearby targets. For the transmission spectrometer (**c**), a large range in response is needed to accommodate both bright LIBS signals and weak Raman signals. A log scale is used to present the gain settings planned for LIBS (2500) and Raman spectroscopy (3200). The response curves of the transmission spectrometer clearly show the three different (green, orange, red) optical windows, with dips in response at the transition regions. The exact position of the transition was selected to avoid any important LIBS emission peaks. ChemCam’s instrument response (Wiens et al. 2012) is shown for comparison. ChemCam is limited to 14-bit numbers, in contrast to SuperCam’s 16 bits

Figure 36 shows the preliminary IRF for the BU spectrometers. This measurement was made with SuperCam mounted in the rover, with the system at room temperature (detectors at $29\text{--}32~^{\circ}\text{C}$). IRF observations were made shortly after integration onto the rover, and again near the end of the rover test period. IRF measurements were also made on the instrument test set-up at LANL, for the EQM MU and the FM BU, and also for the all-EQM instrument. At LANL, a calibrated fiber-fed Ocean Insight DH-2000 lamp was also used to calibrate the FM BU + EQM MU UV range.

As seen in Fig. 36, multiple observations were made using different numbers of integration rows for the reflection spectrometers (UV, VIO; see Table 3 and Fig. 12). The reduction of integration rows from 200 to 40 and 16 allows the instrument to avoid saturation for nearby, bright LIBS targets, especially the rover calibration targets. The EQ-99 lamp was used for calibration of the UV range with the instrument on the rover. However, it was too bright for the 200-row integration, and so this curve was constructed from a scaling factor determined in the earlier IRF measurements that occurred shortly after integration. In this way, the 16- and 40-row curves were scaled up to 200 rows as shown in Fig. 36a. For the VIO and transmission spectrometers, the Labsphere lamp was used for rover calibration, as it avoided alignment challenges incumbent with the EQ-99 lamp. For the data in Fig. 36c, the red window of the transmission spectrometer ($\sim715\text{--}853~\text{nm}$) used a reduced set of 70 rows for integration. This trade accepts slightly lower IRF for better optical resolution in this region.

**Fig. 37:**
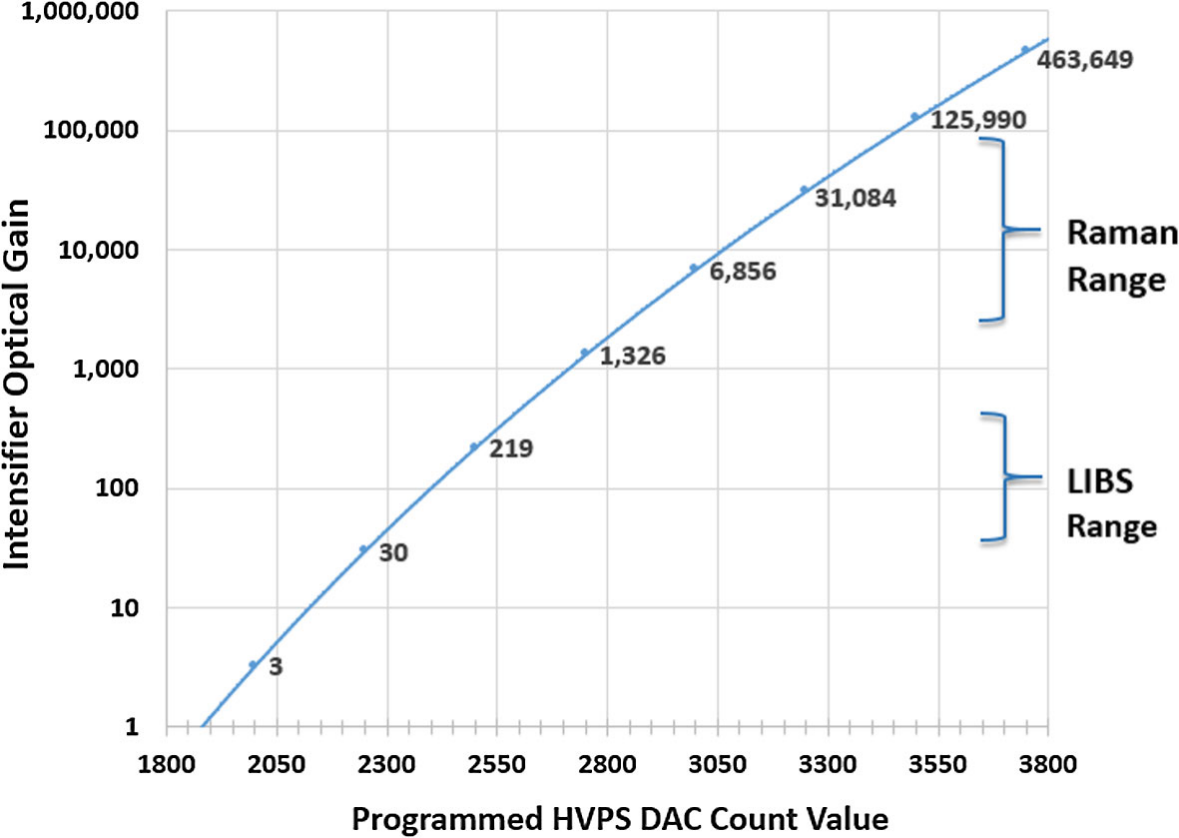
Gain produced across the SuperCam intensifier as a function of the digital-to-analog count value (0–4095). The typical DAC setting for LIBS is 2500; Raman spectroscopy uses a DAC setting of 3200, but these can be adjusted, depending on conditions, e.g., decreased to avoid saturation or increased to highlight a weak feature

The figure also shows that different intensifier gain DAC settings were used for Labsphere lamp observations with the transmission spectrometer (Fig. 36c). The two curves are for the gain settings planned to be used for LIBS (2500) and Raman and TRL spectra (3200). Observations were made at other gain settings, including 2100, 2300, 2600, 2900, 3200, and 3500, spanning a range of over three orders of magnitude in optical gain (see also Fig. 37). Other than scaling factors, these IRF curves at different gain settings appear identical, indicating that the IRF changes in magnitude only, and not in spectral distribution.

Given that the telescope, FOC, and reflection spectrometers are similar to ChemCam’s, a comparison with that instrument is in order. ChemCam’s IRF was measured using 200 rows in all three spectrometers. That instrument’s highest sensitivity in the UV range is $8\times10^{-4}~\text{DN/photon}$ between 280 and 300 nm (Fig. 36a; Wiens et al. 2012). SuperCam’s most sensitive region is at a little longer wavelength, between 295 and 310 nm, achieving $1.7\times10^{-3}~\text{DN/photon}$ using 200 rows of integration (Fig. 36a). The image pixels are effectively identical between the two units, but the digital representation of the pixel intensity is divided into 65536 steps on SuperCam instead of 16384 (see Sect. 3.3.1). At the low-wavelength end of the UV range, both ChemCam’s and SuperCam’s sensitivities drop similarly. In the VIO region, ChemCam achieves just over 1e-3 DN/photon in the 415–440 nm range, while SuperCam’s IRF reaches a maximum of $\sim3.3\text{e-3}$ DN/photon between 380 and 400 nm (Fig. 36b), the result of efforts to provide higher efficiency at shorter wavelengths. The maximum sensitivity in SuperCam’s UV and VIO spectrometers are short of the expected factor of four higher in the digitized signal. The SuperCam demultiplexer was redesigned to favor the Raman spectral range at the slight expense of all shorter wavelengths, so this is not surprising. A slight dip at $\sim425.5~\text{nm}$ indicates the presence of the VIO CCD blemish, giving a maximum drop in instrument response of $\sim4\%$. Because the signal is integrated over many vertical pixels for any given wavelength, after correction for instrument response, we do not expect to see any effect on the processed data.

While the SuperCam transmission spectrometer is very different from the ChemCam reflection spectrometer that covers the same spectral range, we can still compare the IRF at the planned LIBS HVPS setting of 2500 with the IRF of ChemCam in this range. The latter achieves $\sim1.1\times10^{-3}~\text{DN/photon}$, with a maximum at 670 nm. SuperCam’s IRF has two peaks around $1\times10^{-2}~\text{DN/photon}$, one around 580 nm and one near 660 nm (Fig. 36c). The ratio of these two instruments’ sensitivities is more than a factor of four in this case. SuperCam may need to use a slightly lower gain for some LIBS observations to avoid saturating the detector. The strongest LIBS peak in this range is usually oxygen (777 nm triplet), but the Na doublet (589 nm) can also be strong. SuperCam’s sensitivity (using 70 rows in the red) is lower at 777 nm, so oxygen may not saturate, but high-Na targets might saturate at this gain setting.

Considering the IRF from the Raman perspective, SuperCam is most sensitive at a Raman shift of $\sim1600~\text{cm}^{-1}$ relative to the 532 nm laser, and is about half as sensitive in the lower range ($200\text{--}400~\text{cm}^{-1}$). SuperCam’s sensitivity dips at the transition between the green and orange spectral regions, being lowest in the range somewhat below $2650~\text{cm}^{-1}$, but this region has almost no Raman peaks. SuperCam is once again at its most sensitive in the water stretching region ($3300\text{--}3600~\text{cm}^{-1}$).

#### BU Spectral Resolution

The spectral resolution (FWHM of smallest resolvable feature) for the BU spectrometers is driven by the needs of LIBS and Raman spectroscopy for different spectral regions. In the UV and VIO ranges, the density of LIBS emission lines is high, and so a resolution of $\leq0.20~\text{nm}$ FWHM is required. In the longer wavelength region covered by the transmission spectrometer, the emission lines are less dense, and a resolution of $\leq0.65~\text{nm}$ FWHM is desired. For Raman spectroscopy, a resolution of $\leq12~\text{cm}^{-1}$ is needed in the fingerprint region ($150\text{--}1500~\text{cm}^{-1}$). The Raman requirements drove the design of the green spectral range of the transmission spectrometer.

**Fig. 38:**
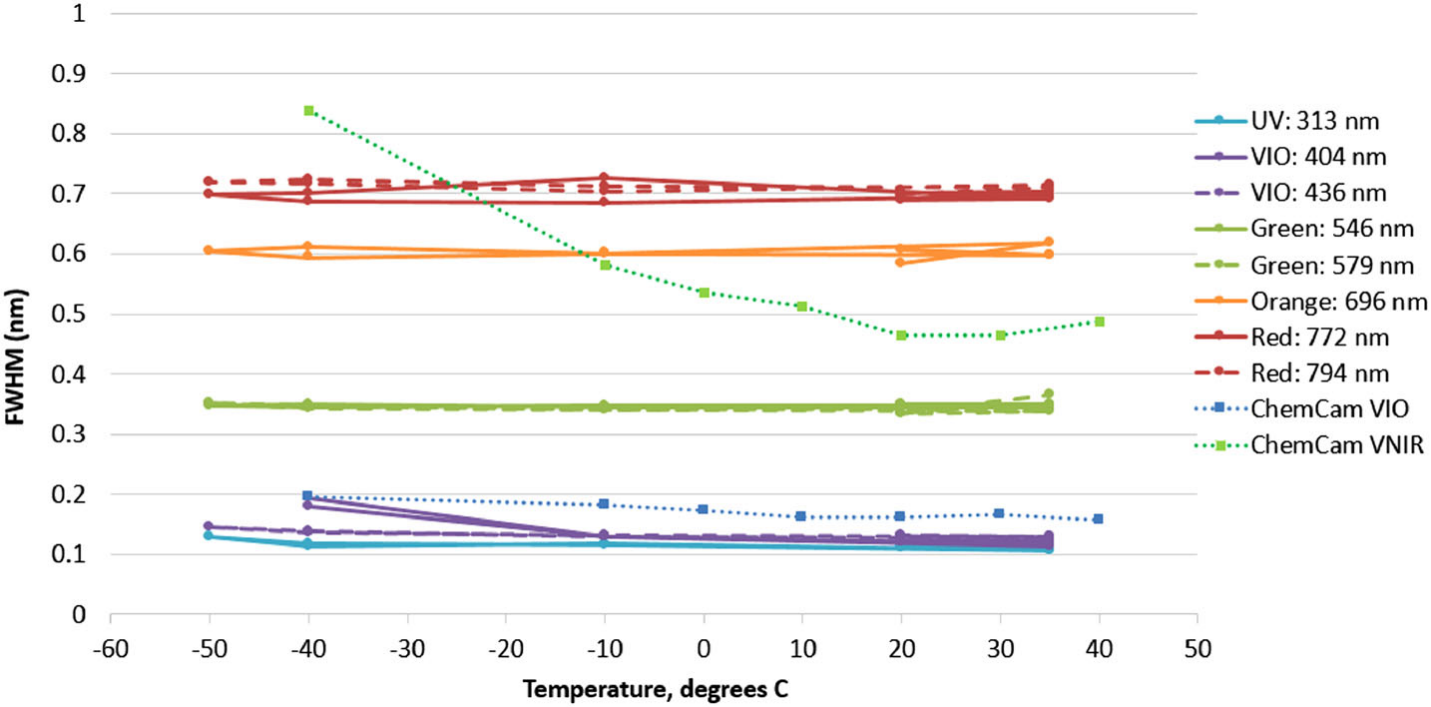
Resolution, indicated as FWHM in nm, at various wavelengths. Atomic emission lamps were used as sources. The observations were made with the SuperCam BU in a thermal chamber at Mars pressure. SuperCam’s reflection spectrometers meet their requirements of 0.2 nm FWHM across the entire temperature range. The Raman fingerprint region, indicated by the green curves at 546 and 579 nm, indicates a resolution in wavenumbers of 11.7 and $10.5~\text{cm}^{-1}$, respectively, meeting the requirement of $12~\text{cm}^{-1}$. The red region, indicated by the two red curves, used an integration over 70 rows, trading some signal for better resolution. ChemCam’s resolution is shown for comparison for VIO and visible and near infrared (VNIR) ranges, measured at 405 and 764 nm, respectively. ChemCam’s UV resolution (not shown) is similar to its VIO resolution

Figure 38 shows the spectral resolution observed from atomic emission lamps with the BU spectrometers in a thermal chamber under Mars atmospheric pressure. LIBS spectra are also shown in Sect. 7.2.1, showing the shapes of the peaks. The resolution of the UV and VIO spectrometers easily meet their requirement, in some cases by almost a factor of two, at $\sim0.12~\text{nm}$ FWHM. In this sense, the SuperCam reflection spectrometers have slightly better optical resolution than their ChemCam counterparts. Two emission lines are shown in the green spectral range, and these two emission lines show a resolution of 0.35 nm FWHM, which translates to 11.7 and $10.5~\text{cm}^{-1}$, respectively. Spectral resolution is not as good in the orange and red spectral regions, but these regions do not have the stringent requirement that the green region has.

This figure also shows the stability over temperature of the peaks. There is almost no change at all over temperature. One of the VIO peaks shows some degradation at $-40~^{\circ}\text{C}$, but it stays within its requirement, and we expect the spectrometers to be within a much smaller thermal range of $0\text{--}35~^{\circ}\text{C}$ on Mars.

#### Wavelength Stability

**Fig. 39:**
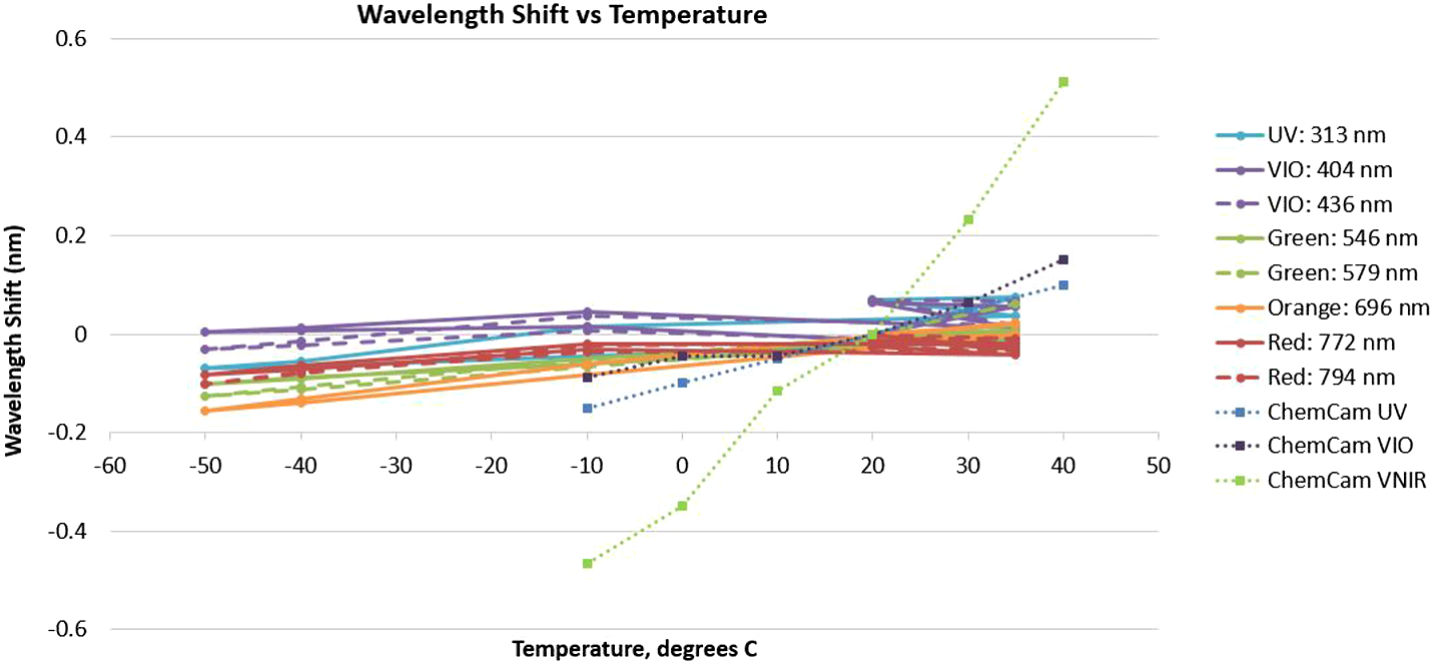
Shift of the position of the incident light on the CCDs as a function of temperature. Construction with titanium instead of beryllium (ChemCam) resulted in a large improvement in thermal stability

The same thermal test was used to determine the stability of the spectrometers with respect to spectral shifts with temperature. Unlike a laboratory spectrometer, where the temperature is kept constant, these spectrometers drift in temperature diurnally by up $\sim20~^{\circ}\text{C}$, and yet the wavelength needs to be known at any given time to within $\sim0.2$ pixels, as accurately as 0.008 nm in the UV, to satisfy the LIBS multivariate analysis algorithms that determine elemental abundances (Wiens et al. 2013). Raman spectroscopy will also benefit from a highly accurate wavelength calibration. Due to thermal expansion and contraction, the wavelengths incident on the CCDs in ChemCam’s reflection spectrometers move by $\sim4$ pixels over the thermal range in which they operate ($0\text{--}35~^{\circ}\text{C}$; Wiens et al. 2012). Fortunately, the mechanical-optical variations are extremely reproducible (over $>2500$ daily cycles on Mars), and so a simple correction is made to the data from each spectrometer. Figure 39 shows the wavelength variations of SuperCam’s BU spectrometers with temperature. The rate of change with temperature is about a quarter of that on ChemCam, thanks to replacing ChemCam’s beryllium spectrometers with titanium ones. The much lower thermal drift make the wavelength corrections much smaller and more accurate for SuperCam. Both Figs. 38 and 39 show some different values in the temperature range around $20~^{\circ}\text{C}$. We believe that is due to settling as the test started, and we expect the temperature-to-wavelength trend to be very stable once the instrument is on Mars.

Wavelength calibration on Mars is expected to be done using LIBS spectra, which contain hundreds of identifiable atomic emission lines. ChemCam uses a Ti plate as a target; SuperCam also has a Ti plate (Manrique et al. 2020) for this purpose. LIBS spectra of other SCCTs can be used almost as well. The algorithm uses a match filter, similar to that described for ChemCam (Wiens et al. 2013).

### LIBS

#### General Spectral Features

**Fig. 40:**
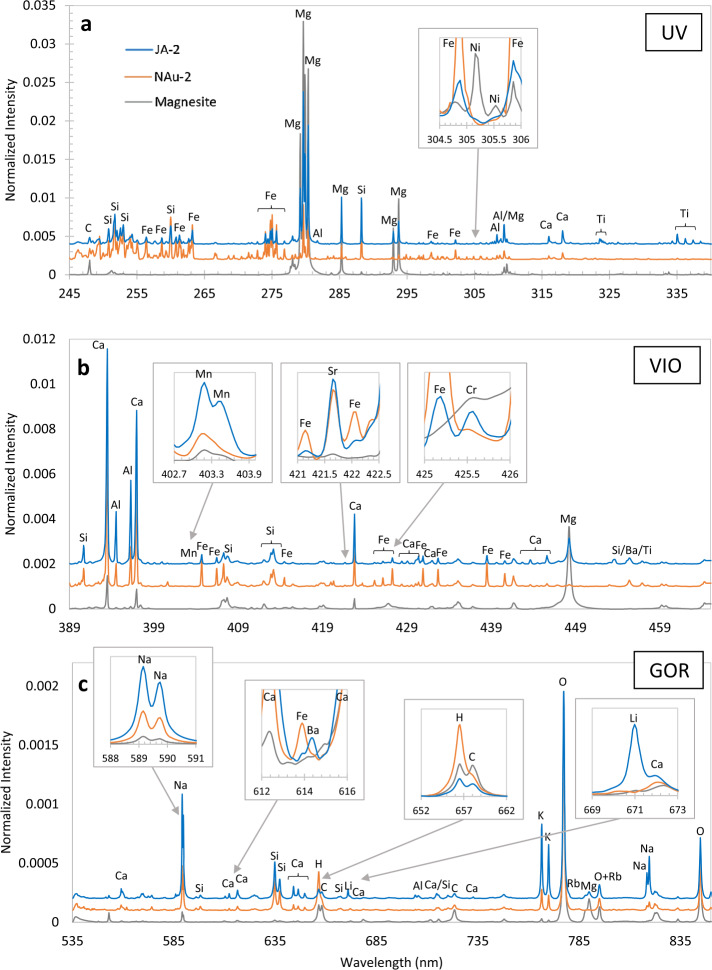
Examples of LIBS spectra taken with the FM BU and EQM MU at LANL. Spectra are normalized to the total emission. Examples were selected to be representative of the expected diversity of materials at Jezero crater. They include an igneous rock (blue, JA-2, andesite from Japan), a smectite clay (orange, NAu-2, nontronite from Australia), and an Mg-rich carbonate (gray, Ni-rich magnesite from Australia). (**a**) UV spectral range with an inset highlighting Ni detections in magnesite. (**b**) VIO spectral range with insets illustrating the detections of Mn, Sr, and Cr. (**c**) transmission spectrometer range (GOR = green, orange, red) with insets showing the Na doublet, Ba, H and C, and Li. This spectral range has improved resolution over ChemCam in the shorter wavelengths as illustrated by the separation of the peaks in the Na doublet, not seen in ChemCam spectra

**Fig. 41:**
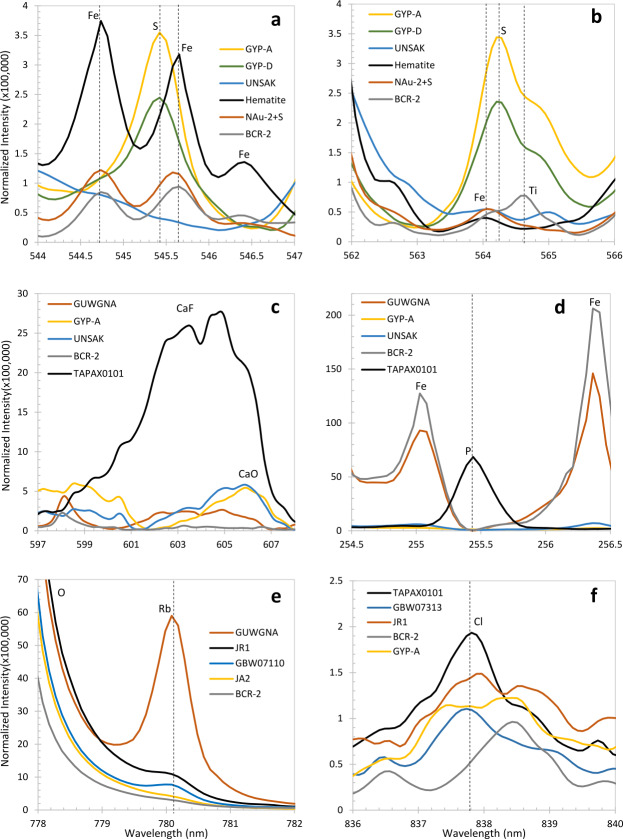
LIBS spectra highlighting various spectral features of interest. The S II 545.5 nm (**a**) and S II 564.2 nm (**b**) peaks are present in gypsum standards GYP-A (46.2 wt.% $\text{SO}_{3}$) and GYP-D (36.70 wt.% SO_3_). Fe peaks are also present in this region as demonstrated by an oolitic hematite (74.96 wt.% total iron as FeO), a sulfur doped Fe-rich nontronite clay (NAu2 plus added sulfur, 15.76 $\text{FeO}_{\mathrm{T}}$ and 21.07 $\text{SO}_{3}$), and a basalt (BCR-2, 12.42 wt.% $\text{FeO}_{\mathrm{T}}$ and 0.04 wt.% $\text{SO}_{3}$). Note the NAu2+S spectrum has an unresolved peak between S and Fe with the skew to the left indicating the presence of sulfur, as opposed to the peak in BCR-2, which is not skewed. UNSAK (aragonite) has very low $\text{FeO}_{\mathrm{T}}$ and $\text{SO}_{3}$, 0.11 and 0.12 wt.%, respectively. (**c**) CaF molecular features (appearing as a very broad peak) are present in apatite standard TAPAX0101 and slightly in GUWGNA (granite with 33,200 ppm F and 0.64 wt.% CaO). CaO molecular features are present in gypsum (GYP-A) and aragonite (UNSAK). No features are present in the basalt (BCR-2). (**d**) A P peak is present in the apatite (TAPAX0101) but not in the other shown spectra, which have low to no P. (**e**) Rb peak on the upper side of a major O peak. The spectra are arranged in descending order of Rb concentration: GUWGNA (Granite) = 2020 ppm, JR1 (rhyolite) = 257 ppm, GBW07110 = 183 ppm, JA2 = 71 ppm, BCR-2 = 47 ppm. (**f**) Cl peaks with the spectra arranged in descending order of Cl concentration: TAPAX0101 (apatite) = several wt.%, GBW07313 (marine sediment) = 40,700 ppm, JR-1 (rhyolite) = 920 ppm, BCR-2 = 98 ppm, and GYP-A = 12 ppm

Figure 40 shows three typical LIBS spectra taken with the FM BU and the EQM MU. The spectra were taken at a distance of 2.85–3.00 m with the samples in $5.8\pm 0.2~\text{Torr}$ of $\text{CO}_{2}$ simulating the Mars atmosphere. The instrument was maintained near $-10~^{\circ}\text{C}$ to maximize laser output. The TECs were not used, as the CCDs were cool without them. Two hundred rows were integrated on each of the reflection spectrometer CCDs. The transmission spectrometer used the usual number of integration rows expected for operation (Sect. 7.1 and Table 3). The targets of andesite, nontronite, and magnesite represent different compositional end-members, namely mafic igneous, phyllosilicate, and carbonate, that could be encountered in Jezero crater, based on orbital observations (e.g., Ehlmann et al. 2008; Goudge et al. 2015, 2017; Salvatore et al. 2018; Horgan et al. 2020). Basalt is expected; however, we used an andesite to provide a more extreme composition in terms of silica, alumina, and alkali elements. The independently-determined compositions of these standards are given in Table 11. The 16-bit data with improved analog-to-digital conversion offer somewhat better dynamic ranges than ChemCam. An in-depth study of detection limits will not be given here, but we note that Mn is observed in all three spectra, indicating a detection limit well below 300 ppm (Table 11). Strontium is easily observed at 134 ppm but probably not at 4 ppm; Cr is easily observed at 450 ppm, but marginally at 40 ppm; Ba is easily observed at 315 ppm but not at 5 ppm, and Li is very easily observed at 29 ppm. In the UV and VIO ranges the spectra look very similar to ChemCam spectra, covering nearly the same ranges with equivalent to slightly higher resolution. The range covered by the transmission spectrometer (green, orange, and red; Fig. 40) is not as large as the third spectrometer’s range on ChemCam (Wiens et al. 2012). SuperCam misses the region from 465 to 535 nm in order to accommodate the Raman spectroscopy. No important emission lines are in that spectral region. The spectral resolution is much better in the green (535–610 nm) range, as illustrated by the Na doublet at 589–590 nm; these two lines are not resolved at all by ChemCam (Wiens et al. 2012). Figure 41 shows more detail of a few emission lines with additional standards. Sulfur emission lines in the range of 540–565 nm benefit significantly from the improved resolution (Fig. 41a, b), as there are a number of interfering emission lines (e.g., Clegg et al. 2019; Rapin et al. 2019). Likewise, the CaF molecular band at 599–607 nm (Fig. 41c) shows additional features relative to the band as observed with ChemCam (Forni et al. 2015). Other minor peaks (Fig. 41d, e, f) such as Rb (780.1 nm), P (255.5 nm), and Cl (837.8 nm) are generally comparable to those observed with ChemCam.

**Table 11:** Compositions of standards for which spectra are shown in Figs. 40–41

	SiO_2_	TiO_2_	Al_2_O_3_	Fe_2_O_3T_	MnO	MgO	CaO	Na_2_O	K_2_O	LOI	Cr	Ni	Rb	Sr	Ba	Li
Ni-magnetite	1.07	0.001	0.08	0.04	0.03	46.21	0.17	0.02	<0.01	50.58	40	2.25%	<2	4	5	
JA-2	56.42	0.66	15.41	6.26	0.11	7.6	6.29	3.11	1.8	2.65	450	134	71	134	315	29.1
NAu-2	48.7	0.595	4.53	31.76	0.02	0.77	1.96	0.7	0.13	11.69						

Abundances are in wt.% for major elements, and in ppm for trace elements (Cr, Ni, Rb, Sr, Ba, Li) except where noted. LOI = loss on ignition

#### LIBS Performance with Distance

**Fig. 42:**
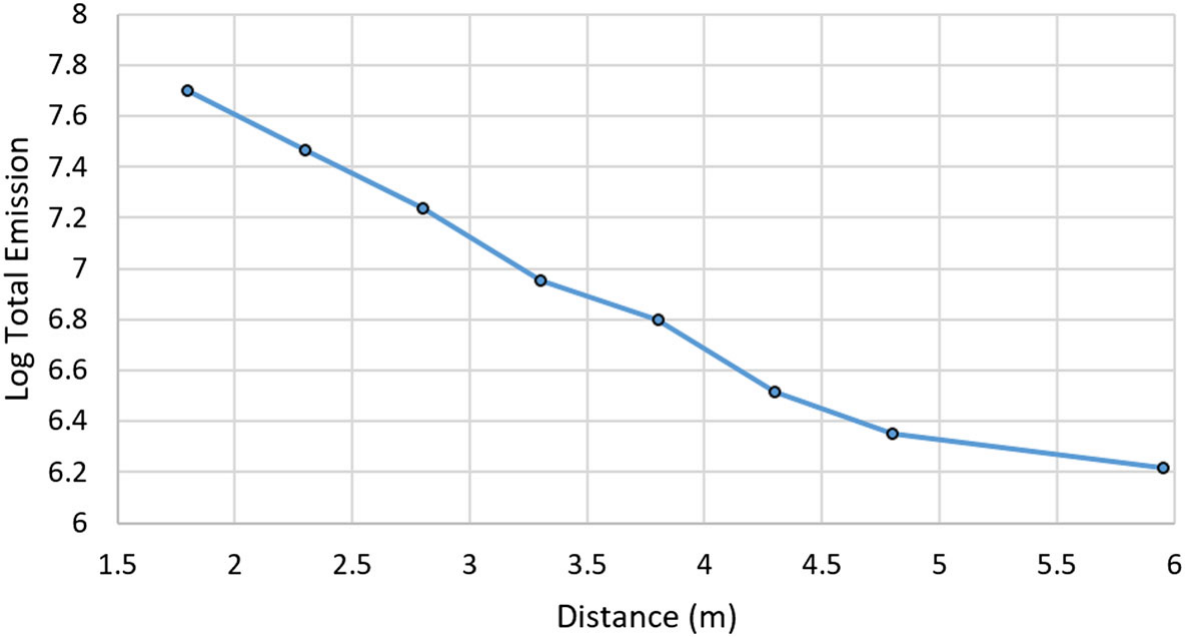
Total emission from target JA-1 as a function of distance

SuperCam’s required distance range extends to 7 m. The complete flight instrument could not be tested under realistic conditions to these distances, as the laser needs to be cooled for optimum performance. The flight instrument was only integrated at JPL, and the rover’s system thermal test—the only time the integrated instrument’s laser was cooled—did not facilitate a 7 m observation. A set of observations was made at increasing distances with the EQM instrument in a thermal chamber at $-10~^{\circ}\text{C}$ using a laser current of 140 A. The target, JA-1, was in air instead of in a Mars atmosphere. Focus was performed manually, as a z-stack. The results in terms of the change in total emission (sum of all channels) is shown up to 6 m (the longest distance performed, due to the size of the room) in Fig. 42. Some of the peaks saturated at distances closer than 2.8 m using all 200 rows of the reflection spectrometers and a nominal gain of 2500 on the intensifier, although the overall emission was not strongly affected, since much of the emission comes from the continuum with the target at ambient pressure. The overall trend with distance is close to $r^{-3}$, where $r$ is the distance. The trend is steeper than the $r^{-2}$ trend expected for passive observations due to the loss of the peak laser power density at longer distances (Maurice et al. 2020). Peaks were still relatively strong (some $> 3000~\text{DN}$) at 6 m, and the FM MU + EQM BU demonstrated LIBS at 7 m (Sect. 6.2), so that achieving the 7 m requirement on Mars is well assured. Unpublished experience with ChemCam indicates that the limiting feature for detection at long distances is the ability of the laser to focus sufficiently well to optically couple with the target and produce a spark. If a spark is produced, the system is always sensitive enough to acquire its associated emission spectrum. (On Mars, ChemCam has succeeded in obtaining weak spectra from some iron meteorite targets as distant as 9.2 m; Johnson et al. 2020). Additionally, on SuperCam, for the spectral region of the transmission spectrometer, the gain can be boosted, which may increase the ability to detect peaks in the green, orange, and red portion of the spectrum at long distances. In terms of quantification, correction for a distance effect in the calibration is needed to provide reasonably accurate observations to 7 m.

#### Time-Resolved LIBS

SuperCam offers the ability to use time resolution for LIBS spectra in the transmission spectrometer, though not in the shorter wavelength ranges. The largest time difference within LIBS spectra is between atomic and molecular spectra. Our studies indicated that essentially all atomic emission is finished within 1 μs, while molecular emission continues over a longer period of time. To check the timing of the CaF molecular peak (Fig. 41c), LIBS was observed from a basalt target doped with nearly 50% CaF_2_ at a distance of $\sim2.3~\text{m}$; this test used the EQM instrument. Two sets of data were taken. In the first set, the intensifier gate was set to 10 μs (long) and the delay was increased to see how much of the CaF peak remained after different times. The other data set used a constant delay of 800 ns (too long to capture all of the atomic emission) and different exposure durations.

**Fig. 43:**
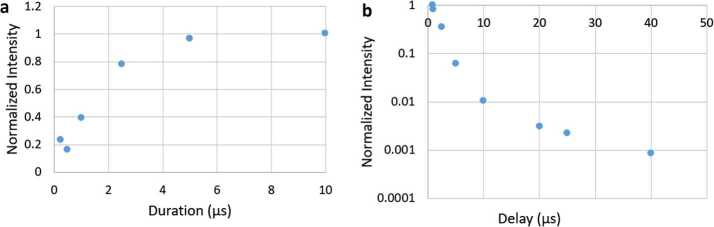
Example of the ability of SuperCam to use time resolution to study LIBS processes. Shown are trends in the intensity of the CaF molecular peak measured at 603 nm as a function of the exposure duration up to 10 μs (**a**), and with up to 40 μs delay, on a log scale (**b**). In (**a**), all exposures start at a delay of 800 ns. In (**b**), all exposures have a duration of 10 μs

The results are shown in Fig. 43. The molecular emission peak persists to $> 40~\upmu \text{s}$ (last data point in Fig. 43b) but most of the emission is gone much earlier; 6% of the emission remains at 5 μs and only 1% at 10 μs (fourth and fifth data points in Fig. 43b). Based on these data, it was decided for the mission to use a LIBS exposure duration of 10 μs with the transmission spectrometer. A relatively short LIBS exposure with the transmission spectrometer decreases the contribution from ambient light. On ChemCam, subtraction of ambient reflected light is a significant factor in this spectral range, and some solar features, especially the Balmer line subtraction for H at 656 nm, can cause problems if the surface albedo changes due to the excavation of a (dark) hole in soil (e.g., Schröder et al. 2015). Relative to ChemCam, which has an effective $\sim8~\text{ms}$ exposure, the ambient light obtained with the transmission spectrometer is reduced by nearly three orders of magnitude, so the no-laser (“dark”) spectra will have much lower signal to be subtracted from the active spectrum. (The LIBS exposure durations for the reflection spectrometers are 1 time tick = 34.133 μs, but the pixels are still active while being read, resulting in the same effective duration as ChemCam. However, ambient light intensity in the VIO and UV ranges are much lower than in the green, orange, and red ranges covered by the transmission spectrometer.)

Several other time-resolution experiments were carried out on LIBS spectra with the transmission spectrometer. One question was whether it is feasible to remove the continuum by starting the exposure slightly later. Most of the continuum is gone within $\sim100~\text{ns}$. However, some of the most rapidly-quenching peaks start to disappear within that time scale, including sulfur peaks. Some spectra could be obtained showing S peaks and almost no continuum. However, SuperCam has some uncertainties on the order of 20 ns due to the two FPGAs that respectively operate the laser and the intensifier (Fig. 29 and Sect. 3.2). Because of this, for SuperCam LIBS data it is best not to try to remove the continuum with the intensifier gate. Other experiments may be of interest, such as temporally separating the H emission line at 656 nm from an overlapping C line (Schröder et al. 2018) or studying molecular emission lines of CaCl (Vogt et al. 2018) or CaO. Finally, it should be possible to collect atomic emissions largely to the exclusion of molecular emissions. This is potentially useful for observing one of the stronger P emission lines which is normally hidden within the CaF structure for minerals like apatite.

#### LIBS Depth Profiles

LIBS has a special advantage of remotely profiling into rocks and soils, so that surface weathering or other surface coatings can be detected and characterized (e.g., Lanza et al. 2012, 2015, 2016). Several targets were profiled using variable numbers of shots per location with the EQM MU and FM BU; additionally, one depth profile was performed in ATLO with the flight configuration. The results may differ between the two because the EQM telescope’s primary mirror had a nickel layer between the structure and the reflective aluminum coating that was later discovered to cause deformation of the mirror at cold temperatures (Maurice et al. 2020). The EQM laser depth profile was performed with the instrument at −5 to $-10~^{\circ}\text{C}$, so the focus may have been somewhat poorer than on the FM, which does not have the Ni layer. Note that ChemCam has a Ni layer (Maurice et al. 2012) because the issue was not discovered until later. In any case, the EQM depth profile should represent a worst case in terms of the SuperCam flight depth-profile capabilities. We present the EQM results first.

**Fig. 44:**
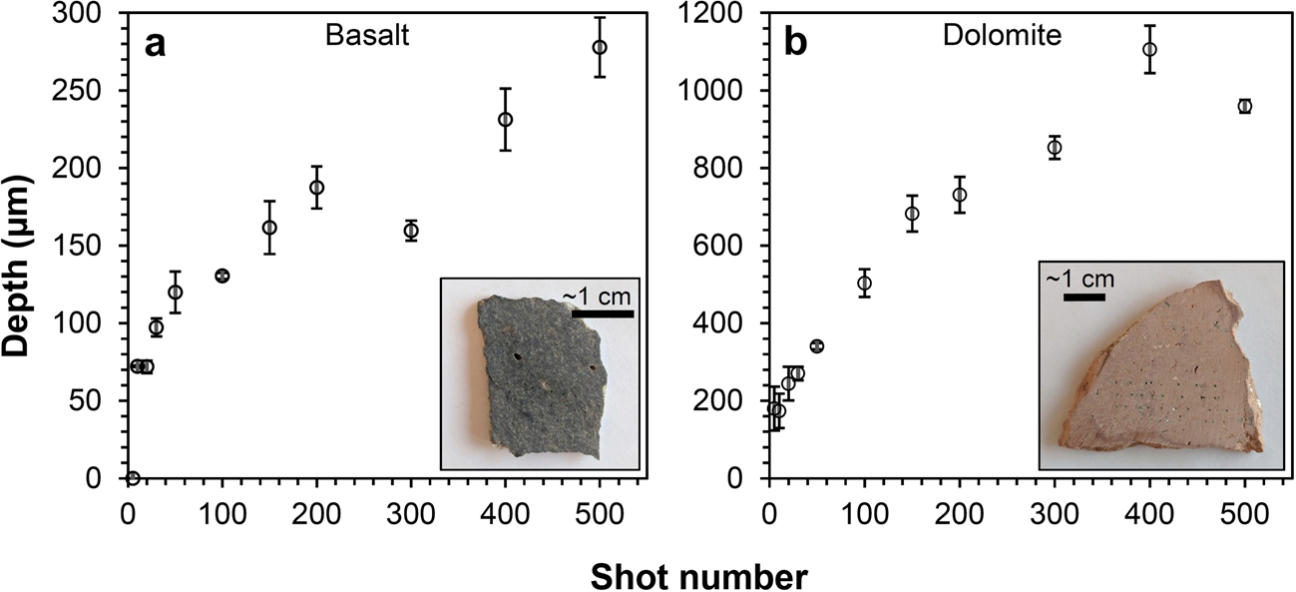
Measured pit depths as a function of the number of laser shots for basalt (**a**) and dolomite (**b**) made with the EQM laser at a distance of 2.86 m in a simulated Mars atmosphere. Error bars show the standard deviations of three pits at each number of shots. Insets show the samples. Rows of pits can be seen in the dolomite sample (**b**)

Two targets were used: a basalt and a sandy dolomite (Fig. 44, insets). The basalt sample is from the $\sim1.5~\text{Ma}$ Black Point Lava Flow, which is part of the San Francisco volcanic field $\sim40$ miles north of Flagstaff, AZ (Ulrich and Bailey 1987); this basalt was also used for the LIBS depth profile experiments described in Lanza et al. (2012, 2015). The dolomite sample is from the Moenkopi formation, Wupatki member, which composes the sedimentary bedrock that predates the lava flow in the same area (Stewart et al. 1972). Both samples were prepared as sawed billets that were not polished but were relatively flat. A series of LIBS shots were performed on each sample to produce pits with 5, 10, 20, 30, 50, 100, 150, 200, 300, 400, and 500 shots per location (3 pits each, e.g., Fig. 44b, inset). These were done at a standoff distance of 2.85 m under a simulated Mars atmosphere. Pit depths and volumes were then assessed using a Keyence VK-X100 3D Laser Scanning Confocal Microscope with a VK-X150 controller. Scanning heights were manually determined by focus at the surface of the sample through to the bottom of the crater. The Keyence MultiFile Analyzer was used to determine crater volume.

**Fig. 45:**
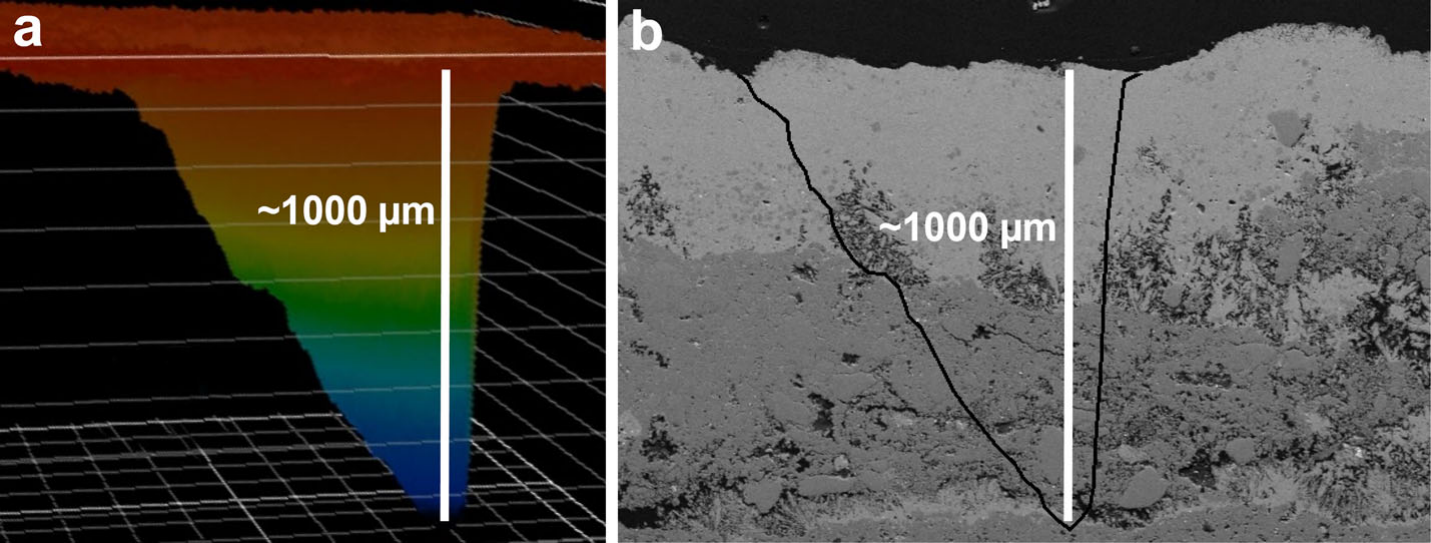
Profile of a laser pit made with 500 shots in dolomite, and a side view of a slice near the surface. (**a**) Profile of the laser pit. (**b**) Backscattered electron image of the same dolomite sample in thin section; the natural exterior of the rock is seen near the top of the image, with the outline of the LIBS pit from (**a**) in black (the pit was not made at this location, but the scale of features can be compared). The weathered surface of the terrestrial dolomite is a different composition than the interior rock, and LIBS depth profiles sample and differentiate between exterior and interior compositions. The LIBS pit is approximately cone shaped but shows some variation due to the properties of the beam

Both the pit depth and the volume of the pit increase rapidly at first, with the rate of ablation decreasing with increasing numbers of laser pulses (Fig. 44). This is expected because the surfaces in the pit are no longer normal to the laser beam as a generally cone-shaped pit develops (Fig. 45). The dolomite ablated more readily than the basalt, producing deeper pits. Pits made with 30 shots (typically used for ChemCam targets; Wiens and Maurice 2015; Maurice et al. 2016) ranged from 84–107 μm deep in basalt and 230–296 μm deep in dolomite. Pits made with 500 shots averaged 280 μm deep in basalt, while 500-shot pits in dolomite were nearly 1 mm deep (920–980 μm), with one outlier point at 400 shots that gave a greater depth of 1100 μm. These results confirm that the material properties of a target, in particular its hardness, play a large role in the total ablation depth that may be achieved with LIBS depth profile analyses. Conversely, the hardness of a target may be inferred by the relative pit size produced by LIBS analysis. The ChemCam FM achieved comparable results (Wiens et al. 2012). On the same samples as SuperCam reports here, the ChemCam laboratory unit achieved comparable results under similar analysis conditions, producing pits with depths $\sim120~\upmu \text{m}$ with 300 shots and $\sim350~\upmu \text{m}$ at 900 shots in the basalt, and $\sim370~\upmu \text{m}$ with 300 shots and $\sim1000~\upmu \text{m}$ at 900 shots in the dolomite (see images in Arvidson et al. 2014).

Depth profile results on Mars may be affected by the stability of the rover and the RSM. For ChemCam depth profiles that used $> 150$ spectra, a pause of over a minute was needed after 150 shots to transfer the spectral data to the rover. It was noted that in at least some cases in which depth profiles of $>150$ shots were made, the first few spectra after the pause showed stronger emission, thought to be due to a slight movement of the center of the beam on the target, possibly only by a few microns. Images of the resultant pits also suggested that there had been a very slight movement during the pause, causing a slight offset in the analysis location between 150 shot bursts. SuperCam will be able to complete 500 shots in a single burst, which will allow greater depth profiling capabilities than ChemCam regardless of mechanical stability.

One depth profile was tested while the FM SuperCam instrument was mounted on the rover, during the system thermal test, with a pressure of 8.1 Torr of N_2_ gas. The target (“ClinQzOrth”) consists of 49% clinoptilolite, 32% orthoclase, and 19% quartz, and was at a distance of 2.6 m. The laser was at $-15~^{\circ}\text{C}$ and used 140 A. A burst of 150 pulses was used, but it was at the same location as an observation using 30 pulses that occurred immediately before, so the pit was made from a total of 180 pulses. The maximum crater depth, measured with the Keyence microscope, is 194 μm, with a crater volume of $1.67\times10^{6}~\upmu \text{m}^{3}$. The bottom of the pit slants slightly to one side; it is not clear if that is due to RSM movement during the depth profile or to the properties of the target or the laser beam. Note that the EQM-laser depth profile shown in Fig. 45 appears similar.

#### Quantitative Elemental Abundances from LIBS

Calibration of SuperCam LIBS will be presented in another paper. Here a brief overview is provided. Pre-processing consists of subtracting a background (non-laser) spectrum, de-noising, wavelength calibration, continuum removal, stitching of the overlapping spectral regions from the transmission spectrometer, and distance correction to provide radiance in photons/second/cm^2^/sr/μm as a function of wavelength. Further processing is done by masking and normalizing the spectra. Quantification will be done using either multivariate or univariate regression. Based on ChemCam experience, it is likely that a multivariate approach will be used for major elements and a univariate approach will be used for minor and trace elements. Quantitative elemental compositions will be reported for eight major elements and at least four trace elements. A spectral library consisting of 332 standards was developed with the FM BU + EQM MU, with targets at 2.86 m distance in a simulated Mars atmosphere. After pre-processing (in progress), the team will optimize and compare multivariate regression models for each element, similar to the methods described in Wiens et al. (2013) and Clegg et al. (2017). For the trace elements, we expect to follow procedures outlined in Payré et al. (2017) and Cousin et al. (2020).

Maintaining calibration over a large range of distances is challenging (Melikechi et al. 2014; Mezzacappa et al. 2016). Unpublished studies with ChemCam data indicate the need for distance corrections for data taken beyond 3.5 m with that instrument. Work is ongoing to apply distance corrections for the SuperCam data.

The rover calibration targets play an important role. Once the rover is on Mars, cross comparison between spectra from Mars and equivalent spectra of replicate targets shot on Earth with a laboratory clone will allow channel-wise comparisons of the results. An “Earth-to-Mars” correction will be applied to the spectra collected on Earth, as it has been for ChemCam (Clegg et al. 2017), to remove any differences between the instruments that remain after correcting for the instrument response. Additionally, the rover calibration targets allow checks of the accuracy of the quantification.

### Raman Spectra

#### Overall Raman Results

The SuperCam Raman technique operates remotely via a telescope using a pulsed laser and a gated detector set to 100 ns exposures. These factors distinguish SuperCam from typical commercial laboratory instruments which use a microscope and thus operate at short distances (i.e. ∼ mm) with small spot sizes (i.e. $\sim\upmu \text{m}$) and use a continuous-wave (CW) laser. Due to the large spot size of 0.74 mrad (the FOV of the spectrometer through the telescope), SuperCam’s Raman mode will likely probe several mineral phases simultaneously, depending on grain size. The Raman signal (lifetime $\approx10^{-15}~\text{s}$) is effectively only produced during the excitation pulse, while other signals like luminescence (lifetimes from minerals are generally $>10^{-9}~\text{s}$) are excited by the pulse but continue to decay long after the laser pulse is finished. The 100 ns gate synchronized with the laser pulse allows efficient rejection of interfering signals like mineral luminescence or daylight entering the telescope, thereby optimizing the collection of the Raman signal. Compared to CW instruments, SuperCam Raman benefits from signal intensification and filtering in the time-domain but uses considerably less excitation, and receives a much smaller fraction of the signal because of the distance.

**Fig. 46:**
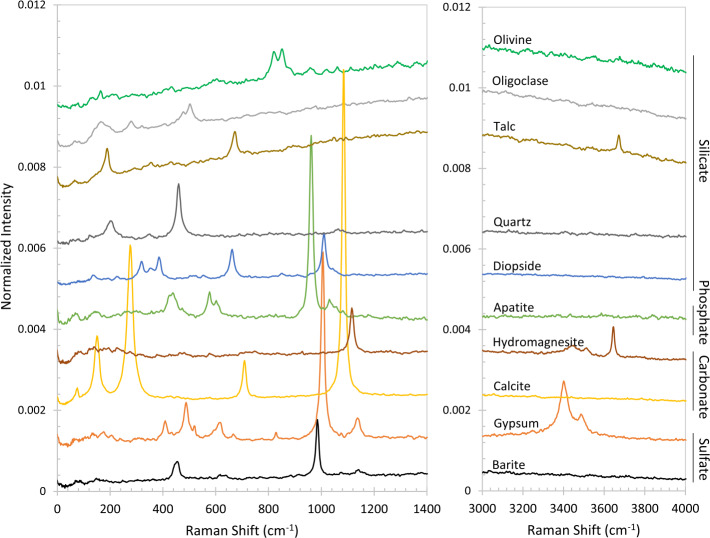
Raman spectra of pure minerals taken using the FM BU and EQM MU. Spectra are averages from 100 laser pulses taken with a delay of 650 ns, a gate width of 100 ns, and a gain of 3200. Laser energy was measured at $\sim8~\text{mJ}$. The distance was 2.77 m. The talc is from an unknown locality while the apatite is from Durango, Mexico, the diopside from Tanzania, the oligoclase from Ontario, Canada, the gypsum from Durango, Mexico, the quartz from Minas Gerais, Brazil, the calcite from Mato Grosso do Sul, Brazil, the barite from Cumbria, UK, the hydromagnesite from Iran, and the olivine from San Carlos, AZ, USA. Spectra of the oligoclase, olivine, and talc have been multiplied by 4 to enlarge the peaks

Representative Raman spectra obtained with the SuperCam EQM MU and FM BU are shown on Fig. 46. Various mineral targets were tested including silicates, phosphates, sulfates, carbonates and accessory phases (e.g., oxides). Most targets were used as raw single crystals but some were prepared as powder pellets. Several organic and natural rock targets were also tested during the campaigns. SuperCam’s spectra are generally high-quality for phosphates, sulfates, and carbonates with clear detection of internal molecular modes and lattice vibrations. Notably, (i) polymorphs can be unambiguously distinguished by studying lattice vibrations (e.g. calcite vs. aragonite), (ii) OH/H_2_O is clearly detected in relevant phases (e.g. gypsum, hydromagnesite, and talc) and (iii) spectral resolution is sufficient to detect differences in subtle Raman shifts of the main peaks due to compositional variations (e.g. Mg vs. Ca carbonates). Some silicates (e.g. olivine, quartz, diopside, and oligoclase) yield well-defined spectra as well, with similar information, while others are challenging to analyze due to low signal (e.g. some phyllosilicates). Accessory phases like opaque minerals are not detectable. Difficulty in detection is mostly due to the high value of a mineral’s optical absorption coefficient that prevents volumetric analysis and/or due to poor Raman efficiency (e.g., Fau et al. 2019); this is consistent with laboratory instruments.

The spectra shown in Fig. 46 were taken under somewhat more favorable conditions than on Mars. First, the minerals were in many cases gem quality or collector’s versions. Minerals of this size and quality will not likely often be found on Mars. However, to challenge any concerns about these crystals being of non-representative sizes, grain-size studies are reported below. Secondly, the instrument was maintained at $-10~^{\circ}\text{C}$. On Mars, the detectors will more likely be close to $0~^{\circ}\text{C}$. Additionally, the intensifier will likely be between 10 and $30~^{\circ}\text{C}$. It is not as sensitive to temperature as the CCDs, but we expect some increased electron background from the intensifier at these temperatures. Both of these factors will add to the noise floor of the instrument.

#### Grain Size Effects

The intensity of Raman spectra has long been known to be affected by the grain size of the target (e.g., Schrader et al. 1991) and the mixtures of grains that are in contact with each other. Remote Raman spectroscopy is more sensitive to this detail than in-situ Raman spectroscopy, where plenty of signal is usually obtained (e.g., Pasteris and Beyssac 2020; Torre-Fdez et al. 2020). Several grain-size studies were carried out with SuperCam prior to delivery; we present the results of two such studies here.

**Fig. 47:**
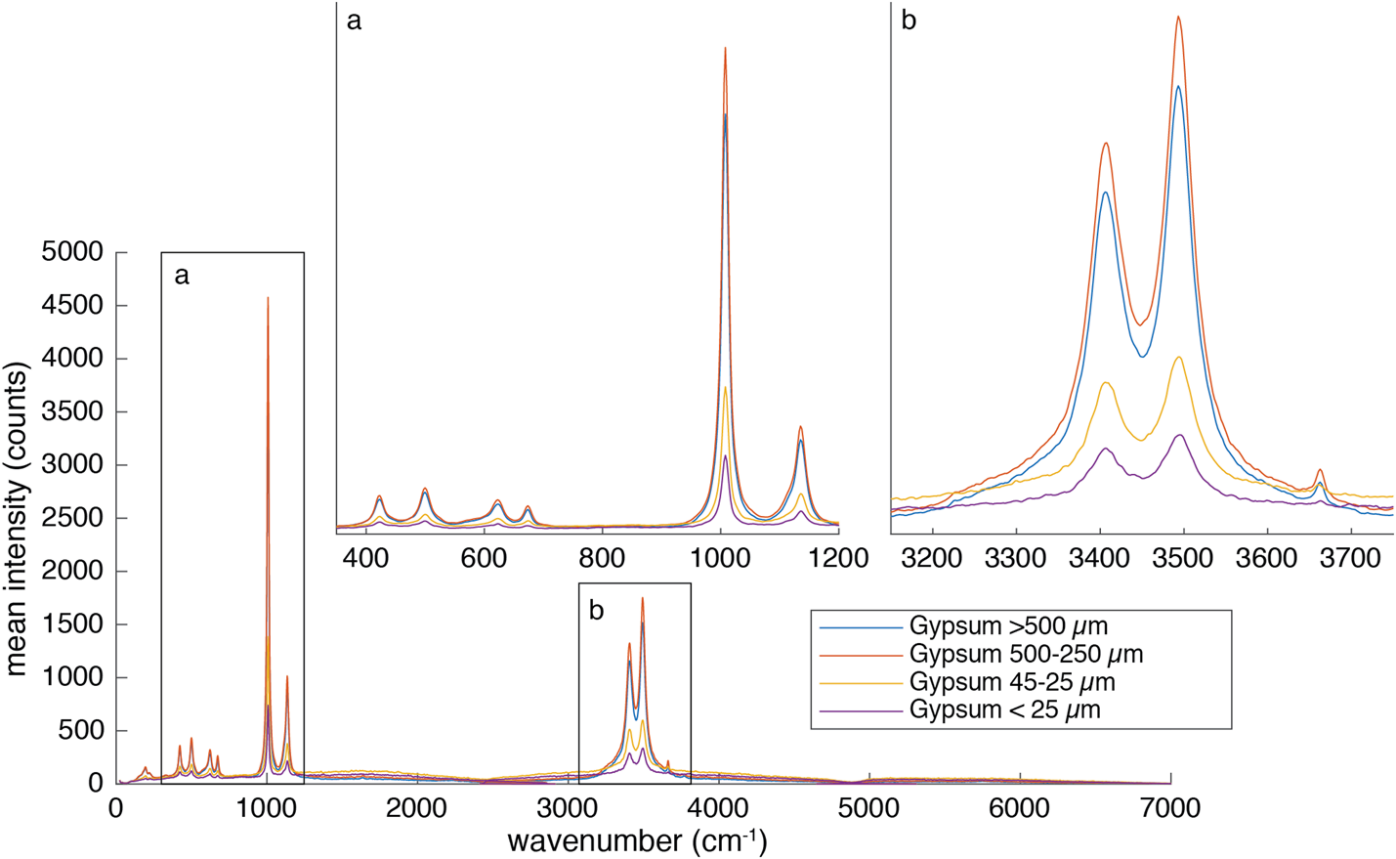
SuperCam Raman spectra of pressed-powder pellets of different grain sizes of selenite gypsum (Glitter Mine, Utah), observed with the EQM at 2.25 m distance integrating 100 laser pulses. Wavenumbers are approximate. Insets show closer detail of the fingerprint (**a**) and water regions (**b**) of the spectrum

**Fig. 48:**
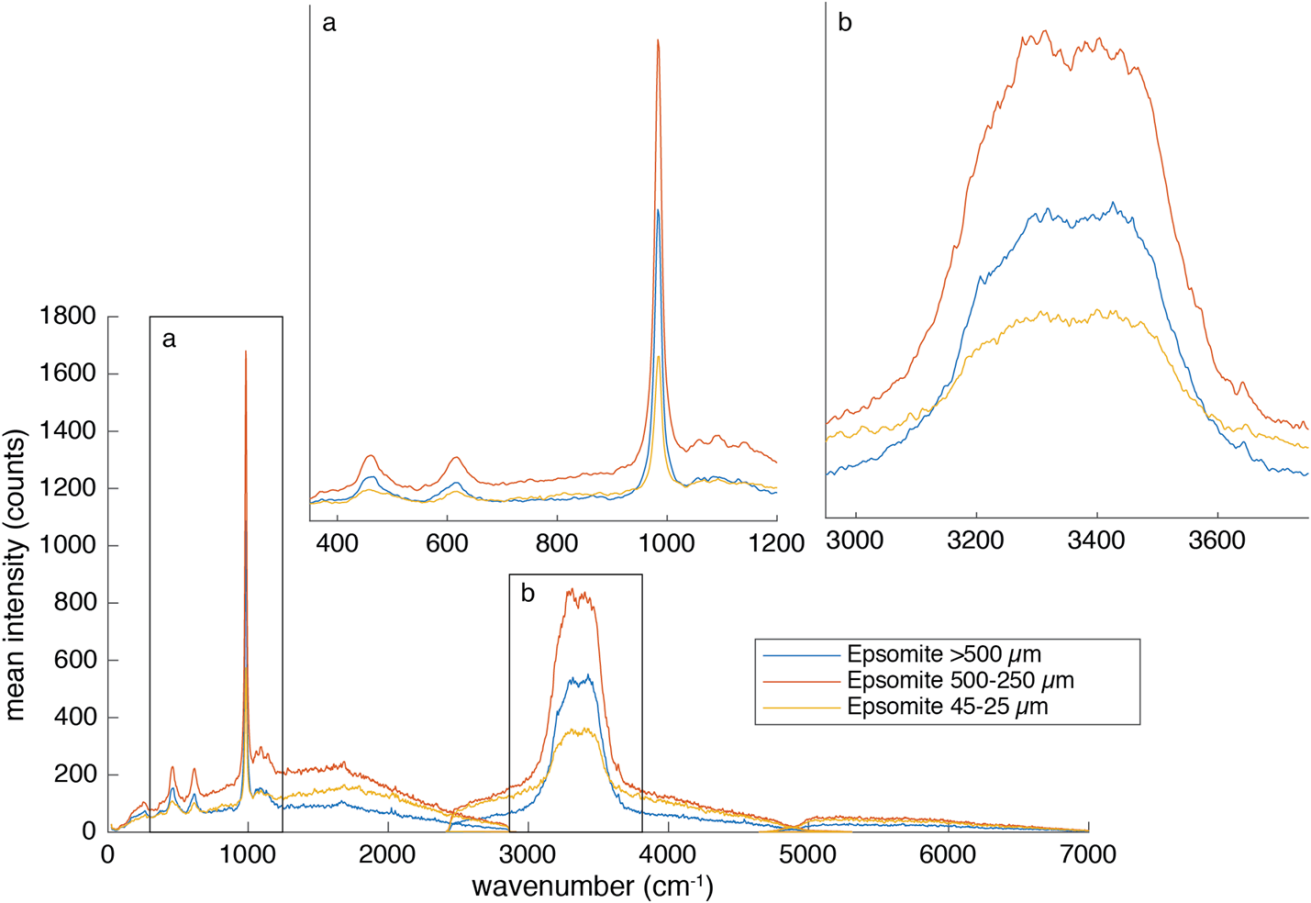
SuperCam Raman spectra of pressed-powder pellets of different grain sizes of epsomite, observed with the EQM at 2.25 m distance integrating 100 laser pulses. Wavenumbers are approximate. Insets show closer detail of the fingerprint (**a**) and water regions (**b**) of the spectrum

Selenite from the Glitter Mine, Utah USA, was cleaned with alcohol, partially crushed in a mortar and sieved through different mesh sizes. Reagent grade Mg sulfate was dissolved in water which was subsequently evaporated to produce large epsomite crystals. These were dried and partially crushed in a mortar and sieved through different mesh sizes. Grains were pressed into pellets and were observed at a distance of 2.25 m from SuperCam’s EQM with the instrument at $<-10~^{\circ}\text{C}$ for maximum laser energy. Figures 47 and 48 show the effects of grain size. In both cases, the fraction with sizes $>500~\upmu \text{m}$ yielded lower signal than the fraction between 250 and 500 μm. Grain sizes $<45~\upmu \text{m}$ showed clearly lower signal than the larger grain sizes, and in the case of selenite, a fraction with grain sizes $<25~\upmu \text{m}$ showed the lowest signal. If these relationships hold for other minerals, it suggests that the best Raman results will be obtained on sandstones with medium to fine grains, although coarse-grained sandstones may perform well too; siltstones and mudstones will give poorer Raman signals on Mars (e.g., Pasteris and Beyssac 2020).

#### Detection Limits and Distance Effects

An important detail in SuperCam’s Raman detection capabilities is the fact that the laser beam covers a significantly larger area than the 0.74 mrad FOV of the telescope and spectrometer for targets at close distance. As distance increases, losses due to the decreasing solid angle of the telescope from the target become significant and temperature-dependent co-alignment of the laser beam and spectrometer FOV affects the signal (Maurice et al. 2020). Here we discuss the number of Raman photons detected from a test made at different distances using the EQM instrument.

The rate at which Raman photons are generated by a given sample is governed by (i) the differential Raman scattering cross-section (i.e. cm^2^ sr^−1^ molecule^−1^) of a target molecule, (ii) the number of molecules of that species that are illuminated, and (iii) the transmitted energy of the excitation source. To gauge the number of Raman photons collected at SuperCam’s entrance aperture and at the photocathode of SuperCam’s intensifier, a cross-calibration study was performed at the University of Hawaii at Mānoa of the Raman scattering cross-section of cyclohexane and Raman efficiency of a pressed pellet sample of gypsum powder. A pressed pellet of powdered gypsum sample was measured by SuperCam and by UH’s Raman spectrometer. In the cross-calibration study, UH’s remote Raman spectrometer, described previously (Gasda et al. 2015), was radiometrically calibrated by a Labsphere calibrated lamp in a manner analogous to that utilized to calibrate SuperCam (Sect. 7.1.1). The radiometric calibration was verified by measuring the differential Raman-scattering cross section of cyclohexane’s $801.3~\text{cm}^{-1}$ Raman mode. Measurements were made with both 1 cm and 1 mm path-length Starna cells, which agreed well. The measured differential Raman scattering cross-section was found to be $4.58\times10^{-30}~\text{cm}^{2}\,\text{sr}^{-1}\,\text{molecule}^{-1}$, which was within 1% of the published values (Trulson and Mathies 1986; Acosta-Maeda et al. 2017) for that differential Raman cross-section. A field-stop, placed at the sample location 6.11 meters from the primary mirror of UH’s Raman spectrometer, circumscribed the projection of the spectrometer’s entrance slit at the sample location so that the laser energy within the FOV of the spectrometer at the sample location could be measured directly. After verifying the accuracy of the IRF, the Raman efficiency of gypsum’s $\nu _{1}$ emission at $1008~\text{cm}^{-1}$ was determined to be $2.04 \times 10^{-8}$, which is in general agreement with published values of gypsum samples (Stopar et al. 2004, 2005).

**Fig. 49:**
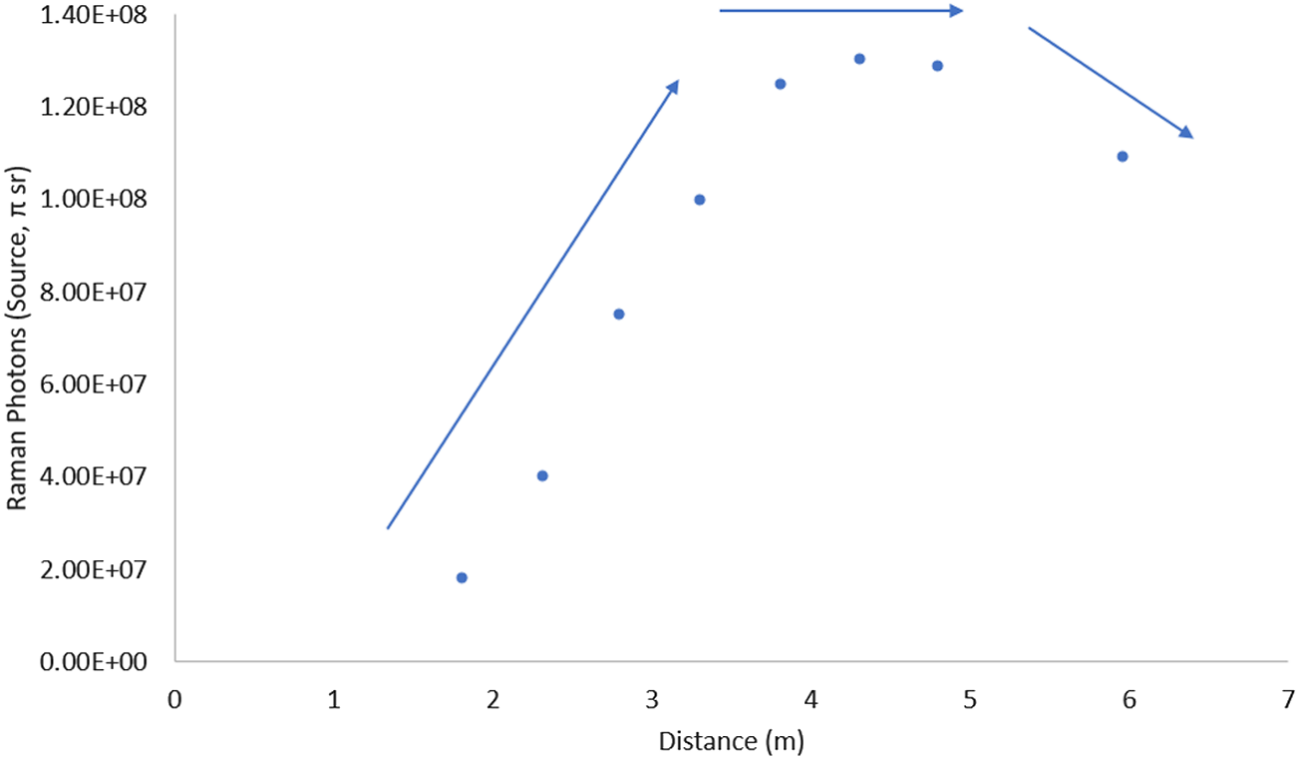
Number of photons generated at the target within the spectrometer field of view (FOV) as a function of distance, for the $\nu _{1}$ mode of gypsum powder using the EQM SuperCam instrument. The result is based on data taken at these distances and knowledge of the spectrometer FOV convolved with an independent determination of the Raman efficiency of the sample. For a given sample, the number of Raman photons produced within the FOV depends on the alignment of the laser beam and the spectrometer FOV, which can degrade beyond a certain distance. The number of photons collected and detected by the instrument is a convolution of this curve with the $1/r^{2}$ losses with distance, as presented in Table 12

The gypsum sample was observed by SuperCam as a function of distance using the EQM instrument. The Raman spectrum of gypsum was corrected from counts (DN) at the CCD to photons at the aperture by application of the IRF. Following the IRF correction, the spectra were corrected by geometrical factors to convert photons at the entrance aperture to Raman photons emitted at the source, assuming Lambertian scattering into $\pi $ sr. The results of this experiment are presented in Fig. 49. Three regions of the plot are readily distinguishable. First, the laser photons per pulse hitting the target (and also the Raman photons produced at the target) within the FOV of the transmission spectrometer increases with distance as the FOV encloses a greater portion of the laser beam (Maurice et al. 2020). Second, the number of laser photons hitting the target within the FOV plateaus as the portion of the laser beam with the highest energy density apparently starts to fall outside of the spectrometer FOV. Third, the laser energy declines as this effect outweighs the expanded spectrometer FOV. The system is sensitive to very slight (i.e. $<0.35~\text{mrad}$) laser-beam misalignments resulting from telescope temperature variations (Maurice et al. 2020), causing the laser spot to deviate relative to the spectrometer FOV, so more or less light is captured by the spectrometer FOV. As the distance increases, the coupling efficiency of the laser spot to the FOV decreases, depending on the amount of mis-alignment.

For highly amplified systems, as is the case with the transmission spectrometer when the intensifier gain is at a high setting for Raman spectroscopy, it is useful to estimate the number of photons being collected to understand the limitations of the technique. This number is not given by the IRF, which also includes the amplification of the photons between the intensifier cathode and the CCD. To determine shot noise and SNR, the number of photons counted at the cathode is the relevant parameter. To determine this from the number of photons in the FOV at the target (Fig. 49), the number of photons entering the instrument aperture was calculated from the solid angle subtended by the instrument, and the transmission from the aperture to the intensifier photocathode was estimated from the transmission of each optic. Depending on the wavelength bin of interest, one to six percent of light entering the telescope aperture reaches the photocathode, with near 6% efficiency at the wavenumber used in this experiment. Table 12 shows the number of photons within the spectrometer FOV at the source, the aperture, and the intensifier photocathode. A minimum of one to ten photons per channel is necessary to achieve detection. SuperCam’s detection limit is wavelength-dependent, requiring the collection of 50 to 150 photons at the aperture. Each peak is at least five channels wide, and Raman observations are expected to use at least fifty laser pulses, so statistics are built up. This calculation was performed with the EQM; the alignment and beam profile is believed to be better with the FM, although the angular size of the laser beam is the same. This exercise, which used a relatively strong sulfate scattering spectral feature, illustrates the challenges of observing Raman signals at long distance.

**Table 12:** Propagation of Raman photons from source to detector for gypsum $\nu_{1}$ emission

Distance (m)	At source	At aperture	At cathode
1.8	1.8e7	4820	310
2.3	4.0e7	4080	260
2.8	7.5e7	3480	220
3.3	1.0e8	2400	150
3.8	1.3e8	1710	110
4.3	1.3e8	1080	68
4.8	1.3e8	690	43
6.0	1.1e8	250	14

Units are photons between the FWHM of the Raman peak per laser pulse. For the source emission, only photons produced within the spectrometer FOV are counted (as in Fig. 49), resulting in an increase between 1.8 and 3.8 m. Numbers are based on measurements made with the EQM. The FM will have different results based on alignment at the time of measurement

### Time-Resolved Luminescence Spectroscopy

TRL is a technique in which molecules or defects in crystals are electronically excited by an incident monochromatic pulsed laser source, and the resulting optical emission is measured in both the time and wavelength domains. Luminescence spectroscopy enables the observation of luminescence centers associated with organic molecules and with trace elements in minerals. Examples of common mineral luminescence centers include rare-earth elements in minerals such as apatite and zircon (e.g. Barbarand and Pagel 2001; Gaft et al. 1998, 2000; Beyssac 2020), $\text{Mn}^{2+}$ in carbonates (e.g. El Ali et al. 1993; Gaft et al. 1998), and $\text{Fe}^{3+}$ in feldspars (e.g. Brokus et al. 2015; Gaft et al. 2005). Luminescence may also occur due to defects caused by radiation damage (e.g. Gaft et al. 2005; Kayama et al. 2014) or shock (e.g. Kayama et al. 2012). When a pulsed laser is synchronized with a gated intensified CCD, portions of the emission from the excited target can be resolved temporally (e.g., Beyssac 2020). In this manner, the decay of the emission may be observed and the luminescence lifetime may be calculated from

$$\begin{aligned} N_{\mathrm{e}}(t) = N_{\mathrm{e}}(0)\text{e}^{-t/\tau} \end{aligned}$$

where $N_{\mathrm{e}}$ is the number of luminescent ions in the excited state, $t$ is time, and $\tau $ is the lifetime (Gaft et al. 2005; Waychunas 2014). Lifetimes may be used to help identify organic compounds, which have a very short (tens to hundreds of ns) lifetime (e.g., Berezin and Achilefu 2010; Lakowicz 2006), and to aid in the identification of various mineral luminescence centers (e.g., Gaft et al. 2005). TRL spectroscopy may be complementary to mineralogical techniques like Raman spectroscopy, where minerals with weak Raman signals may have strong “fingerprint” luminescence emission (Lenz et al. 2013, 2015; Beyssac 2020). It is also complementary to LIBS because it has high sensitivity to rare-earth elements in certain minerals whereas the SuperCam LIBS is less sensitive to them.

SuperCam’s TRL spectroscopy uses the same experimental components as the Raman system (e.g. the green laser and the transmission spectrometer) and takes advantage of the adjustable delay and gate width. The number of different time delays that can be acquired in a single sequence is limited by instrument operational constraints and rover resources. A typical TRL analysis on Mars will likely consist of either a single time delay to determine if luminescence is present or up to five different time delays (Fig. 35) if luminescence is expected. For an analysis with a single delay, first, a luminescence spectrum will be recorded by opening a wide gate just after the laser pulse (luminescence only) or just before the laser pulse (luminescence and Raman). Such spectra will be key to detecting any possible luminescence signal and also to properly identifying the emission center(s) by carefully detecting the different bands corresponding to various electronic transitions. If luminescence is suspected or has previously been observed, additional time delays may be acquired within a single sequence. The maximum number of laser pulses is limited to 200 per TRL sequence. Spectra associated with these pulses may be collected at a single delay to increase the signal to noise or may be distributed among multiple time delays, e.g. five different time delays with 40 pulses per delay. The step size (from one delay to the next) must be equal within a sequence but the size is an adjustable parameter.

**Fig. 50:**
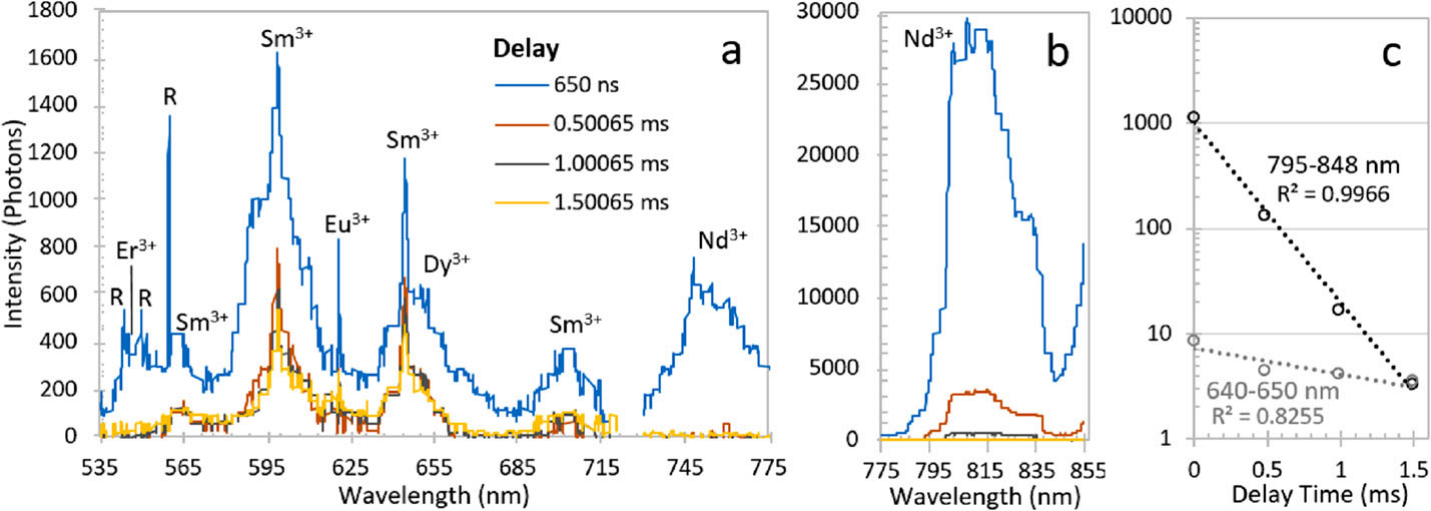
Luminescence spectra of an apatite sample from Durango, Mexico, from 535–775 nm (**c**) and 775–850 nm (**b**); note the difference in intensity between these two regions. A small portion of the spectra around 720 nm have been removed due to excess noise where two spectral windows are joined. Each spectrum is the average of 50 laser shots with a gate width of 0.5 ms. Four time delays starting in the Raman window (650 ns delay) were taken with a 0.5 ms step size. Raman features (marked with an “R”) are present in the first time delay. The other features are attributed to rare-earth element luminescence. (**c**) shows lifetime curves based on the sum of the intensities in the regions between 640–650 nm ($\text{Sm}^{3+}$) and 795–848 nm ($\text{Nd}^{3+}$); the sums have been divided by 10,000 and plotted on a semi-log scale for clarity

Figure 50 shows a luminescence time delay series for a sample of apatite from Durango, Mexico. These data were collected with the EQM BU at ambient temperature and FM MU at cold temperature under semi-dark room lighting during testing in Toulouse, France (Table 8). The distance was 2.98 m. The first delay was taken during the time interval where the Raman signal was active; thus the spectrum is a composite of Raman and luminescence emission. Each spectrum is composed of 50 averaged spectra. The gate width was 0.5 ms and the step size was 50 ms; four steps out to 1.5 ms were completed, similar to how such a series may be collected on Mars. Three Raman features (marked with an “R”) are observed in the first time delay. The other features are attributed to various rare earth elements, e.g. $\text{Sm}^{3+}$, $\text{Eu}^{3+}$, $\text{Dy}^{3+}$, $\text{Er}^{3+}$, and $\text{Nd}^{3+}$ (e.g., Gaft et al. 1998, 2005; Cantelar et al. 2001). While this particular sample was not analyzed for rare-earth abundances, apatite from this locality has been well-studied (e.g., Chew et al. 2016 and references therein) and it can be estimated that these species are present at tens to hundreds of ppm. Panel (a) of Fig. 50 shows the less intense emission from 535–775 nm, while Panel B shows intense emission from $\text{Nd}^{3+}$. Panel C shows two lifetime curves plotted on a semi-log scale over the regions 640–650 nm ($\text{Sm}^{3+}$) and 795–848 nm ($\text{Nd}^{3+}$). The lower $R^{2}$ value for the $\text{Sm}^{3+}$ peak is likely due to a convolution with nearby luminescence centers (e.g. $\text{Dy}^{3+}$) that may have a different lifetime. This may be resolved with careful peak fitting. Additionally, this wavelength range may have additional emission in the first time delay due to contributions associated with the laser pulse, e.g. Raman emission and prompt fluorescence from organic contamination or light diffusion from defects. Calculation of the luminescence lifetimes for these two regions yields $\tau \sim 4~\text{ms}$ for $\text{Sm}^{3+}$ and $\sim 250~\upmu \text{s}$ for $\text{Nd}^{3+}$. The latter corresponds well to the lifetime of 220 μs calculated for $\text{Nd}^{3+}$ emission at 900 nm (Cantelar et al. 2001).

### VISIR

VISIR spectra were obtained during the rover STT (Table 10). It was the only time that the infrared spectrometer was tested on the integrated flight instrument with realistic conditions due to the fact that the spectrometer must be cold and in a Mars-like atmosphere to operate properly. Here we focus on the infrared spectral results.

**Fig. 51:**
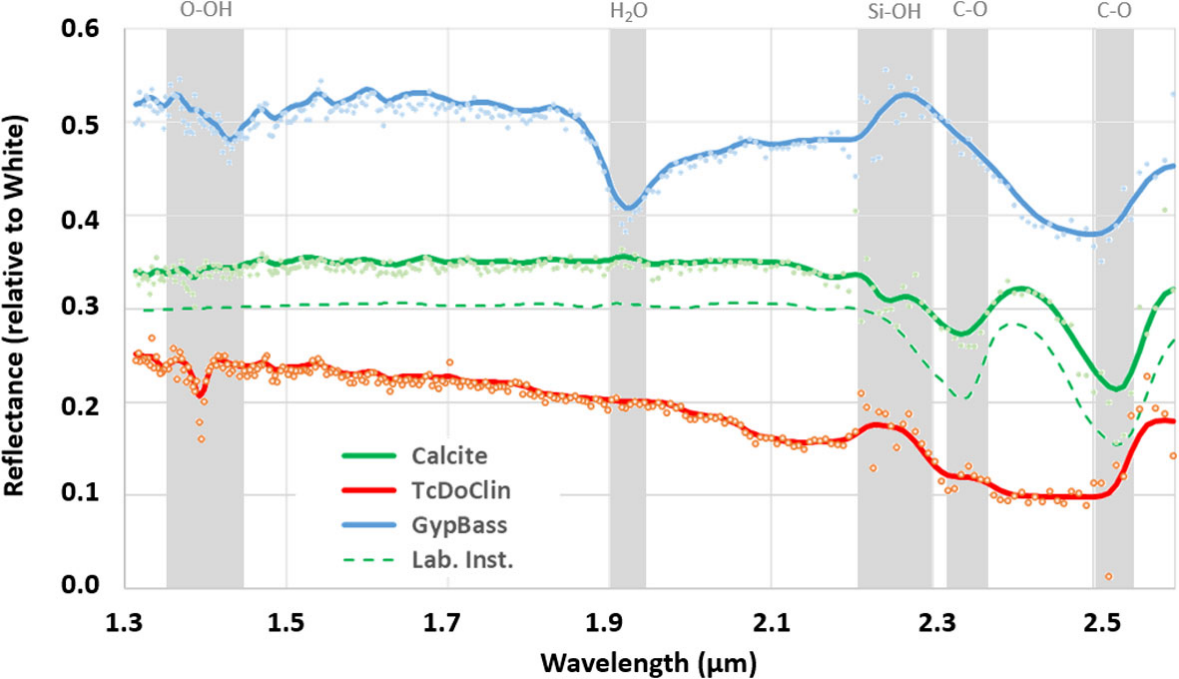
Infrared spectra of several targets observed during the rover system thermal test (STT), referenced to the white standard on the rover calibration target assembly (Manrique et al. 2020). Intensities of individual channels are shown as data points, and shown as a smoothed curve. For the SCCT calcite target, spectra are shown from both SuperCam and from a laboratory instrument described in the text

Figure 51 shows IR results of several observations made during this test. Illumination was provided by Superior Quartz xenon solar simulation lamps at the top of the chamber, set to $700~\text{W/m}^{2}$; these lamps were not spectrally calibrated. The lamps generally have a blackbody spectrum but with a number emission lines in the near infrared mostly below 1.3 μm.

The targets were in two different locations: Calcite and AluWhite (to which the other observations are ratioed) are part of the SCCT assembly on the back of the rover, at 1.56 m distance (Manrique et al. 2020); the GypBass and TcDoClin were located on the floor of the chamber near the rover, $\sim2.6~\text{m}$ from the instrument, and fully illuminated by the solar lamp. The calcite target is a pure mineral that was powdered and spark-plasma sintered (Montagnac et al. 2018) to ensure homogeneity at the 10s of μm scale (Manrique et al. 2020). Of the other targets, GypBass is a mixture of gypsum and bassanite, and TcDoClin is a hand sample containing talc, chlinochlore, and some dolomite. It contains 30 wt.% MgO, 5 wt.% Al_2_O_3_, 4.8 wt.%, CaO, and 2 wt.% C. The latter two samples were baked at $110~^{\circ}\text{C}$ for 48 hours before moving them into the chamber.

The spectrometer temperature ranged from −25.6 to $-30.2~^{\circ}\text{C}$ for observation of the calcite and white standard, and from −21.5 to $-24.6~^{\circ}\text{C}$ for the GypBass and TcDoClin. The IR TEC was set to $-90~^{\circ}\text{C}$ for the calcite and white targets, and $-70~^{\circ}\text{C}$ for the other two. The data were taken with all 256 channels using SuperCam’s IR look-up Table 4, using the auto-exposure feature (Maurice et al. 2020). Actual exposure times per channel ranged from 23 ms for the brightest target to 81 ms for a flat black target.

Data were smoothed using an undecimated wavelet transform algorithm (Starck and Murtagh 1994; Starck et al. 2007) that separates the signal into four frequency scales and reconstructs the signal by the inverse transform; the lowest frequency was used.

Figure 51 shows a comparison between the SuperCam calcite observation during STT and an observation of the same target made with a laboratory instrument. A reflectance spectrum was acquired at L’Institut de Planétologie et d’Astrophysique de Grenoble using the SHADOWS instrument (Potin et al. 2018). The spectrum was obtained in standard mode using a spot size of 5.2 mm in diameter and a spectral resolution of 20 nm (38 nm in the 2.5–2.6 μm range); it was obtained at nadir incidence ($0^{\circ}$) and an emergence angle of $30^{\circ}$. The SuperCam IR results clearly show the carbonate absorptions. The band depth of the C-O adsorption at $\sim2.35~\upmu \text{m}$ is slightly deeper with the commercial instrument than with SuperCam; otherwise the spectra look very similar.

In the case of GypBass, the absorption features at 1.4 and 1.9 μm are slightly farther to the left in the STT observations compared to the laboratory spectra (not shown), and a prominent absorption at 2.25 μm in lab data is completely missing in the SuperCam spectrum (Fig. 51), indicating (e.g., Cloutis et al. 2002, 2006, 2007) that a transition to lower hydration (bassanite, anhydrite) occurred during either or both of the bake and/or the residence time in the test chamber with the rover. Pairs of observations were taken for most STT targets. Comparison between the pairs (not shown) indicates excellent agreement in all cases.

### Microphone

On the integrated SuperCam instrument, the only relevant microphone tests were those that were carried out at Mars-pressure in STT (Table 10). The atmosphere consisted of 7 mbar N_2_ at temperatures of $-55~^{\circ}\text{C}$ and $-80~^{\circ}\text{C}$ for LIBS observations of Ti and ferrosilite SCCTs (Manrique et al. 2020), 1.49 m from the microphone. The test and results are given in Chide et al. (2020). Briefly, the pressure wave recorded by the microphone was $\pm0.5~\text{Pa}$, which is about an order of magnitude above the threshold for detection. The requirement is to detect LIBS plasma sounds to a distance of 4 m. The distance is limited by the poor propagation of sound in the Mars atmosphere. This requirement was not verified directly, but by analysis, the system should meet the requirement. Chide et al. (2020) used the data to investigate the speed of sound at Mars pressure.

### RMI

**Fig. 52:**
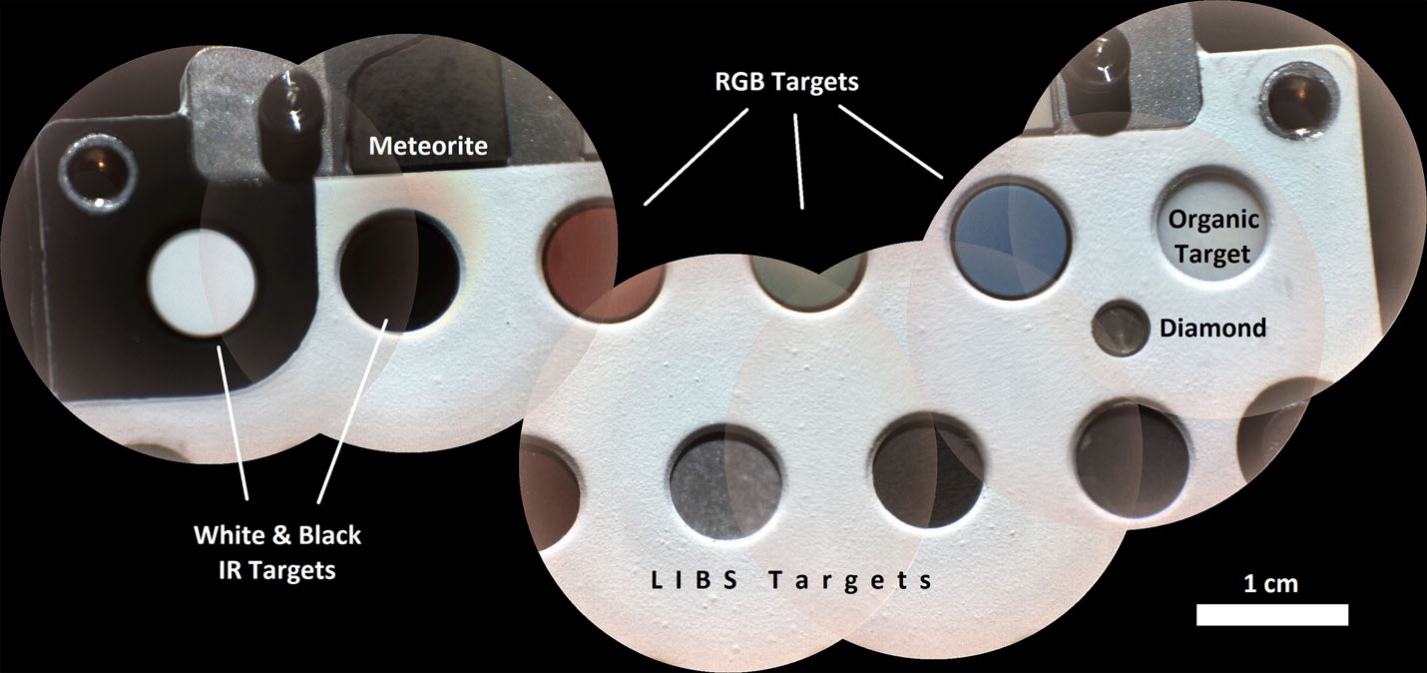
RMI mosaic of part of the SuperCam Calibration Target (SCCT) assembly made from images taken at different times during STT. Some targets are identified. The image on the far right was taken in different lighting; the second from the left is slightly saturated in the red. “Meteorite” is a Mars meteorite sample; “RGB” are red, green, and blue targets (Manrique et al., this volume). Shadows below the fasteners in the upper part of the mosaic indicates the direction of the lighting. Shadows can also be seen below the upper edges of some of the calibration targets

The RMI was tested thoroughly after SuperCam was integrated on the rover. Initial testing was for basic functionality of image acquisition and RMI-based autofocus. The RMI was used for inputs to a camera parallax model that also involves the Mastcam and Navcam imagers. Eighty-nine RMI images were taken during STT, and 4 to 5 times more were used to perform 27 RMI-based autofocus activities. These were conducted at different temperatures, and under different lighting conditions (halogen and Sun simulator) with images of the SCCT (at $\sim1.6~\text{m}$; Fig. 52), on rocks at two different distances (2.6 and $\sim4.4~\text{m}$), and on a boresight test target (dark panel with white disks at $\sim1.9~\text{m}$) to derive and check the camera model.

The RMI autofocus was validated during STT. As expected, the completely uniform targets are more challenging, especially the bright ones, because of the risk of saturation. Focused images were obtained along with good spectra in the same sequences, demonstrating that the focus offsets between imaging and spectral collection are well characterized (see Maurice et al. 2020). Apart from the caveats below, the auto-exposure function worked with both halogen and Sun-simulator illumination. The associated parameters (see Maurice et al. 2020) are currently more tolerant of darker images rather than saturated images, and they may need to be adjusted on Mars. Integrations from 2 to 600 ms were obtained with the Sun simulator, and up to $\sim34~\text{sec}$ with halogen illumination. With the Sun simulator, exposure times of $<10~\text{ms}$ were selected by the auto-exposure algorithm for the bright paint of the SCCT under a phase angle $>62$ degrees and the brightest rocks at short distance on the floor under of phase angle of $\sim20$ degrees. In these cases the auto-exposure is subject to rounding error (to the lower integer in ms) which becomes significant at these low values. However, typical integration times on Mars should be closer to 20 ms, which was indeed the case in STT with lower albedo rocks or higher phase angles. The dark offset measured in the non-illuminated corners of the images uses less than 5% of the full dynamic range of the sensor, as expected.

**Fig. 53:**
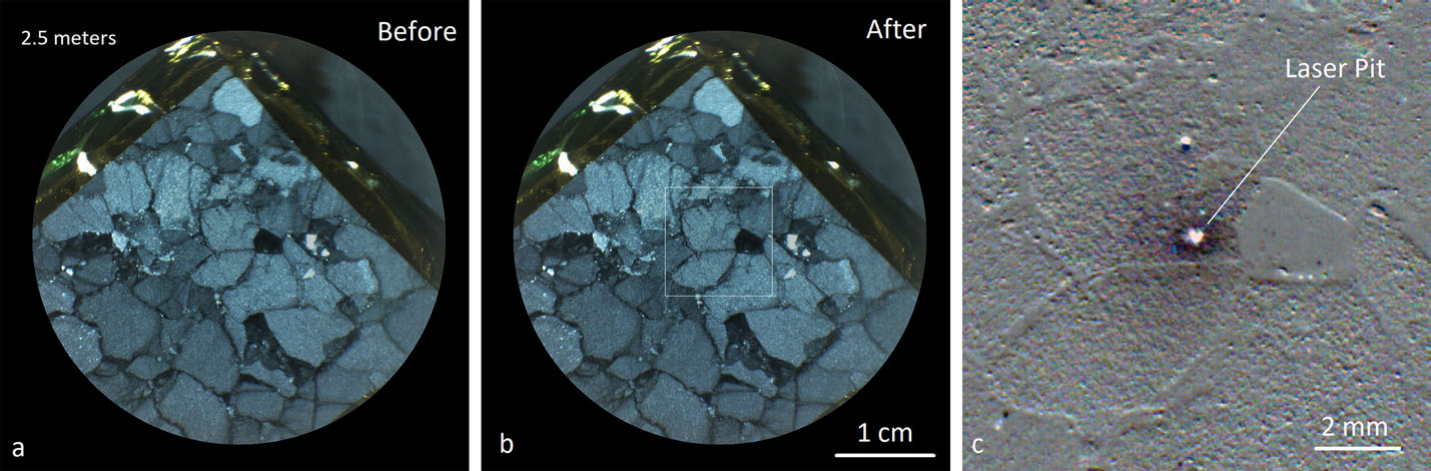
RMI images taken during STT of a composite target (62% Ilmenite, 38% Hematite) at 2.6 m, using 19 ms integration time. (**a**) Before LIBS, 30 shots. (**b**) After LIBS. (**c**) Difference of the two images to highlight the laser pit. This level of detail is expected of most RMI images taken in the rover’s arm work zone and slightly beyond

A great level of detail is visible in the images, which will be very valuable for documentation of the SuperCam targets. The laser pits are generally visible depending on the target hardness and properties (Fig. 53). The pit is located at the expected RMI coordinates, proving that the LIBS-RMI alignment has not changed.

## Conclusions and Prospects

The SuperCam instrument responds to the Mars 2020 Science Definition Team’s (SDT) request to “combine mineralogy, texture, and ideally, chemistry observations at a scale comparable to that of the grains within rocks” (Mustard et al. 2013). By placing these capabilities within a single instrument, co-boresighted for remote sensing, these observations can go beyond the reach of the arm, covering $>100~\text{m}^{2}$ with all techniques from a single rover position. SuperCam also responds well to the SDT request for analyses of the borehole created by the drill (Mustard et al. 2013), a capability that is highly important to the dossier of information to be collected on the cached samples.

SuperCam is particularly well suited for Jezero crater, with both IR and time-resolved Raman spectroscopies well capable of identifying and characterizing carbonates (e.g., Ehlmann et al. 2008). Other expected compositions (summarized in Mangold et al. 2020), such as serpentines and the precursor primary silicates (e.g., olivine) should all be easily identifiable with SuperCam. The choice of rover calibration targets (Manrique et al. 2020) was tuned toward the compositions expected at Jezero.

In this paper, we have documented the details of the SuperCam BU, including the designs of the optical spectrometers that enable LIBS, VIS, time-resolved Raman, and TRL spectroscopies. The laser stimulus to create these signals, as well as details of the IR, RMI, and MIC investigations, are described in the companion paper by Maurice et al. (2020). The results of the integrated instrument, given in Sect. 7, show that SuperCam meets all of its requirements and will provide well-balanced scientific results covering elemental chemistry, mineralogy, and physical properties of the targets, allowing the best possible geological interpretations at a range of distances and size scales.

The development of SuperCam, including the novel remote time-resolved Raman and luminescence spectroscopies, opens the door to exploration of other planetary bodies with these techniques, especially in places where organic materials are known to exist or are anticipated, such as Ceres, Europa, Enceladus, and other outer bodies including comets (e.g., Fray et al. 2016; De Sanctis et al. 2017; Postberg et al. 2018). Incorporation of these techniques in a remote-sensing mode facilitates the type of reconnaissance needed to direct sampling strategies for instruments that use corers or scoops to provide a much more limited number of (perhaps more diagnostic) analyses with in-situ instruments (e.g., Hand et al. 2017). A prototype variant of SuperCam already includes an imager that uses a nanosecond-level time-resolved luminescence mode to uniquely capture locations of organic signatures within a scene and characterize them (e.g., Wiens et al. 2020). On future missions, the Raman and TRL spectroscopies presented here could be used not only as standoff techniques, but also in situ.

SuperCam also provides the first true near-infrared capability operating at ground level. Following the numerous successes of near IR spectroscopy from orbit (e.g., Bibring et al. 2004; Murchie et al. 2007), the community has waited a long time to have this capability at grain-size scales. While SuperCam provides this as a point spectrometer and not an imager, this apparent reduction in capability is compensated by the removal of dust using the laser, overcoming a significant limitation to IR analysis on the ground. The complementarity with time-resolved Raman spectroscopy is highly important for the identification of the full range of minerals present in any given outcrop. SuperCam’s point analyses are also complementary to the multispectral imaging analyses that will be done by Mastcam-Z (Bell et al. 2020, this journal).

Another original aspect is the microphone, not only for public outreach, but more importantly for its contribution to Mars science. For atmospheric processes, the coupling with MEDA (Manfredi et al. 2020, this journal) is noteworthy for the determination of wind characteristics and turbulence of the atmosphere over an unparalleled range of frequencies. For LIBS, the microphone implements an added diagnostic technique that has been used in the laboratory for a number of years. SuperCam’s capability to couple the microphone and LIBS is unique (e.g., Chide et al. 2019), and will give access to physical properties (such a rock hardness), providing an analysis that is very similar to a “hammer test,” one of the first things a geologist does on a field trip.

SuperCam’s imager (RMI) has a small field of view compared to the other cameras on board, but its contribution is paramount to the success of the investigation. As requested by the SDT, it is essential to document the context (morphology, texture) of each chemistry and mineralogy observation, and to place them into the broader perspective given by the Navcam (Maki et al. 2020, this journal) and Mastcam (Bell et al. 2020, this journal) imagers.

One aspect of SuperCam is largely yet to be realized within this Mars 2020 mission, and that is the synergy that comes from fusion of the results from the different techniques. This data fusion can be applied within the SuperCam investigations, or at the level of all techniques on the Mars 2020 rover (Farley et al. 2020). Studies to date include one early field study by a small team representing various instruments (Martin et al. 2020), an exercise on a “mystery rock” within the SuperCam team (Ollila et al. 2019), and several remote operations and science team training (ROASTT) events organized within the Mars 2020 project (Lawson et al., in preparation). All of these were much more limited in scope than is expected to be experienced with the Perseverance rover covering multiple outcrops over a large field area (Sun and Stack 2020). We expect the synergy experienced at both the human level, and with tools such as machine learning, will provide surprising discoveries that would not be possible without combining the results of the individual techniques.

## Data Availability

Data presented in the Results section of this paper are being made available to the Planetary Data System Geosciences Node under Mars 2020/SuperCam.

## Appendix of Abbreviations, Acronyms, and Short Definitions

ADC Analog-to-digital converter
AEGIS Autonomous exploration for gathering increased science: a software package to analyze Navcam images onboard and select SuperCam targets
AFT Allowable flight temperature
AOTF Acousto-optic tuning filter, performs wavelength selection for the IR spectrometer
APG Annealed pyrolytic graphite
Be Beryllium
BU Body unit
C&DH Control and data handling (electrical board)
CCD Charge coupled device
CDR Calibrated data record
CMOS Complementary metal oxide sensor
CNES Centre National d’Etudes Spatiales
CTE Coefficient of thermal expansion
CWL Continuous-wave laser
DAC Digital-to-analog converter
DC Direct current
Decon Decontamination
DN Digital number
EDR Experimental data record
EDU Engineering development unit
EMI/EMC Electromagnetic interference and electromagnetic compatibility
EQM Engineering qualification model
FITS Flexible image transport system (file format)
FPGA Field programmable gate array
FOC Fiber optic cable
FOV Field of view
FM Flight model
FWHM Full width at half maximum
GOR Green, orange, and red spectral regions covered by the transmission spectrometer
HGA High-gain antenna
HSS High-speed serial
HVPS High-voltage power supply
Hz Hertz
ICER Lossy progressive wavelet image compression
IR Infrared
IRAP Institut de Recherche en Astrophysique et Planetologie, the lead SuperCam institution in France
IRS Infrared spectrometer
J Joules
JPL Jet Propulsion Laboratory
kHz Kilohertz
LANL Los Alamos National Laboratory
LIBS Laser-induced breakdown spectroscopy
Lpmm Lines per millimeter
LVDS Low-voltage differential signal
LVPS Low-voltage power supply
Mbps Million bits per second
MHz Megahertz
Mrad Milliradians
MRAM Magnetoresistive random-access memory
MSL Mars Science Laboratory
MU Mast unit
mV Millivolt
NA Numerical aperture
Navcam Navigation camera for the Mars 2020 rover
Nd:YAG Neodymium-doped yttrium-aluminum garnet
P43 Phosphor screen used in the image intensifier
QE Quantum efficiency
PCB Printed circuit board
PROM Programmable read-only memory
RAMP Rover accessory mounting plate, the main mounting surface for instruments and electronics in the interior of the rover body
RCE Rover compute element (computer)
RMI Remote micro-imager
RSM Remote-sensing mast
RTG Radiothermal isotope generator
RTV Room-temperature vulcanizing
SAGE Venus Surface and Atmosphere Geochemical Explorer
SCCT SuperCam calibration targets
SDRAM Synchronous dynamic random-access memory
SE Spectrometer electronics (board)
SHERLOC Scanning Habitable Environments with Raman and Luminescence for Organics and Chemicals, a UV Raman spectrometer mounted on the Perseverance rover’s arm
SOH State of health
Sr Steradian
STT System thermal test, rover-level
SUROM Start-up read-only memory
TEC Thermal-electric cooler
Ti Titanium
TRL Time-resolved luminescence
TTL Transistor-transistor logic
UART Universal asynchronous receiver-transmitter
UV Ultraviolet spectral range ($\sim245\text{--}340~\text{nm}$) covered by one of SuperCam’s two reflection spectrometers
V Volts
VIO Violet spectral range ($\sim385\text{--}465~\text{nm}$) covered by one of SuperCam’s two spectrometers
VIS Visible spectral range encompassing the violet, green, orange, and red spectral ranges, used for passive reflectance spectroscopy
VISIR Visible and infrared passive reflectance spectroscopy
W Watts
XDR Extracted data record
